# Non-invasive radiogenomic mapping of the SMARCAL1-driven ferroptotic niche is associated with longitudinal MRD-negative surveillance in early-stage NSCLC

**DOI:** 10.3389/fimmu.2026.1835413

**Published:** 2026-05-25

**Authors:** Zehao Huang, JunDao He, Yue Li, ZhanYu Xu, Huajian Peng, Xiang Gao, HuaFu Zhou, JianJi Guo, Nuo Yang

**Affiliations:** Department of Thoracic Surgery, the First Affiliated Hospital of Guangxi Medical University, Guangxi, China

**Keywords:** CXCL9/10-CXCR3 axis, ferroptosis, molecular residual disease, non-small cell lung cancer, radiogenomics, SMARCAL1, spatial transcriptomics

## Abstract

**Background:**

Post-operative molecular residual disease (MRD) detection using circulating tumor DNA (ctDNA) has been established as a sensitive biomarker for early recurrence risk stratification in early-stage non-small cell lung cancer (NSCLC). However, the biological heterogeneity of patients MRD-negative at baseline remains incompletely characterized, and a proportion subsequently experience MRD conversion and radiographic recurrence. We investigated whether pre-operative radiomic features could non-invasively capture the spatial organization of a ferroptosis-enriched, immune-active tumor microenvironment associated with sustained post-operative MRD negativity.

**Methods:**

In this single-center translational cohort nested within the prospective CTONG 2201 trial (NCT05457049), 71 patients with completely resected stage IB–IIIA NSCLC, MRD-negative at two post-operative landmarks, were followed under dynamic observation without immediate adjuvant therapy. A Rad-Score was derived from 1,432 pre-operative contrast-enhanced CT radiomic features using a pre-specified five-layer pipeline. Whole-exome sequencing, bulk RNA-seq with CIBERSORTx, and 10x Visium spatial transcriptomics in 14 tumors (five High, four Intermediate, five Low Rad-Score; 36,318 quality-controlled spatial spots) characterized molecular correlates. Multiplex immunofluorescence (n = 40 across Rad-Score quartiles), immunohistochemistry (n = 60), CRISPR-Cas9 knockout of SMARCAL1 with wild-type and helicase-dead rescue in A549/H1299 cell lines, and primary human CXCR3^+^ CD8^+^ T cell chemotaxis assays provided protein-level and functional validation. External validation was performed in two independent cohorts (TCIA NSCLC-Radiogenomics, n = 178; Lung3, n = 89) with locked-model application, following Harrell optimism-corrected internal validation (B = 1,000 bootstrap replicates with full-pipeline resampling).

**Results:**

Nine stability-validated radiomic features (selection frequency Π ≥ 0.60, all; ≥ 0.80 for 7/9) constituted the Rad-Score. High Rad-Score patients had longer MFS (HR = 0.32, 95% CI 0.18–0.58; *P* < 0.001), with apparent C-index 0.812 attenuating to optimism-corrected 0.798 and externally validated 0.783 (NSCLC-Radiogenomics) and 0.741 (Lung3). The Rad-Score remained independent after adjustment for 13 clinical covariates including ECOG PS, Charlson Comorbidity Index, and smoking pack-years (adjusted HR = 0.66, *P* = 0.022), outperformed five established comparators (CALGB 9761-type, Kratz 14-gene, ctDNA composite, Bindea immunoscore, clinical+TMB) in decision curve analysis across the clinically meaningful threshold range 0.15-0.75. High Rad-Score tumors exhibited elevated TMB (*P* < 0.001, BH-FDR q < 0.001) and APOBEC signatures (SBS2, SBS13: q < 0.001; SBS4 non-significant after FDR), enriched CD8^+^ T cells and M1 macrophages (q < 0.01), and contiguous ferroptotic niches with monotonically increasing activity across the Rad-Score continuum (Spearman ρ = 0.87). SMARCAL1 knockout conferred resistance to erastin-induced ferroptosis (cell viability 78 ± 6% in sgSMARCAL1 cells versus 31 ± 4% in sgScramble controls, *P* < 0.001) and IFN-γ–induced CXCL9/10 secretion (62%/57% reduction) — phenotypes rescued by wild-type but not helicase-dead SMARCAL1 — and functionally attenuated CXCR3^+^ CD8^+^ T cell transmigration (58% reduction), with CXCR3 antagonist AMG 487 producing >90% blockade.

**Conclusions:**

In this externally validated, functionally interrogated translational cohort, a pre-operative CT-derived Rad-Score is associated with sustained MRD-negative surveillance, provides a non-invasive surrogate for a SMARCAL1-driven ferroptotic-immune niche, and requires prospective validation Non-small cell lung cancer; Molecular residual disease; Radiogenomics; Spatial transcriptomics; Ferroptosis; SMARCAL1; CXCL9/10-CXCR3 axis.

**Clinical trial registration:**

## 1 Introduction

Surgical resection is the standard curative treatment for early-stage (IB–IIIA) non-small cell lung cancer (NSCLC) (1). Despite complete macroscopic and microscopic (R0) tumor clearance, approximately 30–50% of patients experience disease recurrence, frequently due to occult micrometastases (2). Circulating tumor DNA (ctDNA)-based detection of post-operative molecular residual disease (MRD) has emerged as a sensitive modality for recurrence risk stratification in early-stage NSCLC (3, 4). MRD positivity at post-operative landmarks is being actively investigated as a biomarker to guide adjuvant therapy decisions in interventional trials (e.g., MERMAID-2, NCT04642469, evaluating durvalumab in MRD-positive patients; ctDNA-guided adjuvant design in NCT05079022 and related trials), though conclusive evidence of survival benefit from MRD-directed therapy remains pending. The clinical management of baseline MRD-negative patients remains uncertain: a proportion of such patients subsequently experience MRD conversion and radiographic relapse (5), yet adjuvant systemic therapy in this unselected group carries substantial toxicity without demonstrated benefit. These observations highlight a clinically unmet need to identify intrinsic tumor characteristics that may influence sustained post-operative MRD negativity in the baseline MRD-negative subpopulation — a subpopulation that currently defaults uniformly to dynamic observation under trials such as CTONG 2201.

Long-term MRD negativity is associated with effective clearance of residual micrometastases by systemic immune surveillance. The tumor microenvironment (TME) shapes systemic antitumor immunity, particularly the activation and expansion of CD8^+^ T cells prior to surgical resection (6). Alternative regulated cell death pathways, particularly ferroptosis — an iron-dependent, lipid peroxidation-driven form of regulated cell death — have been recognized as immunogenic processes that can release damage-associated molecular patterns (DAMPs), prime dendritic cells, and promote CD8^+^ T cell recruitment (7–9). Ferroptosis-related gene expression may therefore reflect aspects of the TME relevant to systemic immune surveillance. The SWI/SNF-related, matrix-associated, actin-dependent regulator of chromatin subfamily A-like 1 (SMARCAL1) is an ATP-dependent annealing helicase that resolves stalled DNA replication forks during replication stress (10, 11). Genomically unstable tumors — such as those enriched for APOBEC-related mutational signatures characteristic of heightened replication stress — may rely on SMARCAL1 activity and co-express ferroptosis susceptibility programs including ACSL4-mediated phospholipid remodeling (12, 13). The spatial organization of SMARCAL1-associated ferroptosis in NSCLC and its relationship to sustained MRD negativity have not been previously characterized.

Comprehensive assessment of TME states traditionally requires multi-region tissue sampling, limiting clinical applicability (14). Radiomics — the extraction of quantitative spatial features from standard medical imaging — offers a non-invasive approach to infer tumor-intrinsic properties (15, 16). Radiogenomics bridges imaging phenotypes with underlying genomic and transcriptomic characteristics, potentially providing insight into the TME and ferroptosis–immune interactions (17). Previous radiomic models in NSCLC have predominantly predicted overall survival or stage using the intratumoral core alone, often without spatially resolved biological validation and without external validation (18). Contemporary reporting standards for AI/ML-based prognostic models (TRIPOD-AI (19); IBSI-II (20)) require rigorous pipeline stability assessment, optimism-corrected internal validation, and external cohort validation in geographically or temporally independent populations — standards that prior radiomic NSCLC MRD studies have largely not met.

To address these gaps, we hypothesized that pre-operative CT-derived radiomic features can non-invasively capture the spatial organization of a SMARCAL1-driven ferroptotic niche and its associated immune-active microenvironment in early-stage NSCLC, and that such features are associated with sustained post-operative MRD-negative surveillance. We tested this hypothesis through a preplanned single-center translational cohort analysis nested within the CTONG 2201 trial (NCT05457049), integrating multi-modal CT radiomics, whole-exome sequencing, bulk RNA-seq, 10x Visium spatial transcriptomics, multiplex immunofluorescence, and CRISPR-Cas9 functional validation, with external validation in two independent public cohorts.

## 2 Methods

### 2.1 Study design and ethical oversight

This study was designed as a preplanned single-center translational cohort analysis derived from patients enrolled at the First Affiliated Hospital of Guangxi Medical University, a participating center in the prospective CTONG 2201 study (ClinicalTrials.gov identifier: NCT05457049). CTONG 2201 is a prospective clinical study evaluating a dynamic observation strategy in patients with completely resected stage IB–IIIA NSCLC who achieve longitudinally undetectable MRD after surgery. The present work was conducted as a nested radiogenomic and spatial transcriptomic substudy focusing on baseline MRD-negative patients managed without immediate adjuvant therapy.

The study protocol, including tissue acquisition, radiological imaging, molecular profiling, and longitudinal follow-up, was conducted in accordance with the Declaration of Helsinki and was approved by the Institutional Review Board and the Independent Ethics Committee of the First Affiliated Hospital of Guangxi Medical University (Approval No. 2023-K152-01). All participants provided written informed consent before study-specific procedures, including consent for genomic sequencing, spatial transcriptomic profiling, and the use of de-identified clinical and radiological data for research analyses. A supplementary ethics approval (2026-K0033) was obtained for the protein-level validation studies (multiplex immunofluorescence, immunohistochemistry) on archived FFPE tissues, primary human CD8^+^ T cell isolation from healthy donor PBMCs, and functional CRISPR-Cas9 experiments in established NSCLC cell lines.

### 2.2 Patient cohort and longitudinal dynamic observation protocol

#### 2.2.1 Inclusion/exclusion criteria

Patients were derived from the CTONG 2201 cohort at the First Affiliated Hospital of Guangxi Medical University. Of 145 screened patients with completely resected NSCLC, 71 met all inclusion criteria: (1) histologically confirmed primary NSCLC; (2) pathological stage IB–IIIA (AJCC 8th edition); (3) complete R0 resection with systematic mediastinal lymph node dissection; (4) adequate pre-operative contrast-enhanced CT quality; (5) adequate FFPE and fresh-frozen primary tumor tissues. Exclusion criteria: (1) ctDNA-defined MRD positivity at either post-operative landmark (n = 31); (2) unprotocolized neoadjuvant/adjuvant therapy (n = 28); (3) insufficient tissue (RIN < 7.0) or non-correctable CT artifacts (n = 15).

#### 2.2.2 Clinical variable retrieval

Two independent clinicians blinded to Rad-Score status systematically retrieved 13 additional clinical variables from the electronic medical record, with discrepancies resolved by consensus review. These variables, all recorded at the pre-operative evaluation visit or within 30 days of surgery. For the three patients missing pack-years and seven missing FEV1, multivariable Cox regression analyses were performed using both listwise exclusion (primary) and multiple imputation (m = 5 chained equations via the mice R package v3.15.0; predictors: age, sex, pathological stage, BMI, ECOG PS) as sensitivity analysis. Directional findings were consistent across both approaches.

#### 2.2.3 Longitudinal dynamic observation protocol

All included patients underwent a two-landmark MRD assessment strategy: peripheral blood samples at post-operative days 3–7 (landmark 1) and at approximately 1 month (landmark 2). Plasma cell-free DNA was analyzed using a tumor-informed customized NGS panel tracking a median of 16 (IQR 12-20) patient-specific variants per individual at 100,000× unique molecular coverage depth, with a variant detection threshold of 0.02% variant allele frequency. Only patients with confirmed MRD negativity at both landmarks were included. For continuous ctDNA metric analyses, we additionally recorded mean variant allele frequency (VAF) across tracked variants at each landmark (even below the binary detection threshold) and derived: (i) maximum VAF across both landmarks; (ii) VAF change between landmarks (Δ VAF); (iii) sum of VAF across tracked variants.

#### 2.2.4 Endpoint definitions

Primary endpoint: MRD-Free Survival (MFS), defined as interval from R0 resection to first confirmed ctDNA-positive result or death. Secondary endpoint: Disease-Free Survival (DFS), defined as time from surgery to radiographic recurrence or death.

#### 2.2.5 SMARCAL1 transcriptomic stratification

Raw RNA-seq counts were normalized to Transcripts Per Kilobase Million (TPM) with subsequent log2(TPM + 1) transformation. Given the absence of an established clinical cutoff for SMARCAL1 in early-stage NSCLC, we employed a non-parametric median split on the full N = 71 cohort log2(TPM + 1) distribution (median = 6.48). Patients above median were assigned to the SMARCAL1-High group (n = 35); those ≤ median to the SMARCAL1-Low group (n = 36).

### 2.3 CT acquisition and standardized pre-processing

Pre-operative chest CT examinations were acquired across three multi-detector CT platforms (Somatom Definition AS, Siemens Healthineers; Discovery CT750 HD, GE Healthcare; Brilliance iCT, Philips Healthcare). Standardized parameters: tube voltage 120 kVp; tube current 100–200 mAs with AEC; rotation time 0.5 s; pitch 0.8–1.0. Iohexol 300 mg I/mL 80–100 mL at 2.5–3.0 mL/s, venous phase at 60–70 s post-injection. Reconstruction with standard/medium-smooth convolution kernel (**Supplementary Figure 2**.). Standardization pipeline per IBSI guidelines: (i) isotropic resampling to 1.0 × 1.0 × 1.0 mm³ via 3rd-order B-spline interpolation; (ii) fixed-bin-width intensity discretization (25 HU); (iii) ComBat harmonization (empirical Bayes framework) with scanner manufacturer as batch covariate, preserving the outcome covariate per Horng et al.

### 2.4 High-throughput radiomic segmentation and feature extraction

#### 2.4.1 Segmentation protocol

Three-dimensional volumetric segmentation was performed semi-automatically using 3D Slicer. A senior thoracic radiologist (> 10 years’ experience), blinded to clinical and genomic outcomes, delineated the primary gross tumor volume. A second senior radiologist independently verified; discrepancies with Dice Similarity Coefficient (DSC) < 0.90 were resolved by consensus. This primary GTV served as the intratumoral core region of interest (ROI_core). We used ROI_core for the intratumoral core and ROI_rim for the 3-mm peritumoral invasive margin throughout. The prior inconsistency with “ROI_rim” has been corrected manuscript-wide. The 3-mm peritumoral invasive margin (ROI_rim) was generated by morphological dilation of the ROI_core outward by 3.0 mm, with meticulous exclusion of adjacent air spaces, bones, and major blood vessels. The 3-mm radius was selected *a priori* based on preliminary sensitivity analysis across 2, 3, 5, and 10 mm dilation thresholds.

### 2.4.2 Feature extraction

Radiomic feature extraction complied with IBSI mathematical definitions using PyRadiomics v3.0.1 in Python v3.9. From both ROI_core and ROI_rim, 1,432 quantitative imaging features were extracted encompassing: (1) first-order statistics; (2) 3D shape descriptors; (3) Gray Level Co-occurrence Matrix (GLCM); (4) Gray Level Run Length Matrix (GLRLM); (5) Gray Level Size Zone Matrix (GLSZM); (6) Neighborhood Gray Tone Difference Matrix (NGTDM). Laplacian of Gaussian filters (σ = 1.0, 3.0, 5.0 mm) and wavelet transforms (8 frequency bands: LLL, LLH, LHL, LHH, HLL, HLH, HHL, HHH) were applied.

#### 2.4.3 Reproducibility assessment

Feature-level reproducibility was assessed in a 30-patient subset independently segmented by two senior thoracic radiologists (10 and 12 years’ experience). Intra-observer agreement was assessed by Reader 1 repeating segmentation after a 4-week washout period. For each of the 1,432 features, intra-class correlation coefficients (ICC) were calculated; only features with both intra-observer and inter-observer ICC ≥ 0.80 were retained, yielding 1,105 reproducible features entering the dimensionality reduction pipeline.

### 2.5 Hierarchical dimensionality reduction and stability-assessed rad-score construction

#### 2.5.1 Layer 1 — reproducibility filtering

Features retained only if both intra-observer and inter-observer ICC ≥ 0.80. This reduced 1,432 → 1,105 features.

#### 2.5.2 Layer 2 — low-variance and redundancy filtering

Features with near-zero variance (coefficient of variation < 0.05) were excluded. Remaining features underwent hierarchical agglomerative clustering based on Spearman rank-correlation distance (d = 1 − |ρ|) with cut-off at d = 0.20 (|ρ| ≥ 0.80 collapsed). The medoid feature (highest mean absolute correlation to cluster members) was retained as cluster representative. This reduced 1,105 → 129 features.

#### 2.5.3 Layer 3 — univariate Cox pre-screening

Each surviving feature was evaluated by univariate Cox regression against MFS. Features reaching nominal P < 0.05 were retained. This biologically-motivated reduction concentrates statistical power on features with *a priori* outcome association (Parmar et al., Sci Rep 2015; Lambin et al., Nat Rev Clin Oncol 2017). This reduced 129 → 42 features.

#### 2.5.4 Layer 4 — LASSO Cox with nested cross-validation

The 42 pre-screened features entered LASSO Cox regression. The penalty parameter λ was determined via nested 5 × 5 cross-validation, replacing the original single-loop LOOCV. Nested CV is essential because tuning λ and evaluating performance on the same CV folds yields optimistically biased estimates. The outer 5-fold loop provides unbiased generalization error estimate; the inner 5-fold loop selects λ. Objective function:


β^ = argmin {−(1/N) ∑i[yi· xiTβ−log(1+exp(xiTβ))]+λj∑|βj|}

At optimal λ_min, LASSO selected 9 non-zero features, identical to the original model (supporting robustness of feature selection even under the more stringent pipeline).

#### 2.5.5 Layer 5 — stability selection

To formally assess feature-selection stability, stability selection was performed with B = 500 subsamples, each drawn at 50% of the cohort without replacement. For each subsample, the full pipeline from Layer 2 onward was re-executed, and selection frequency (Π) of each feature was recorded. Features achieving Π ≥ 0.60 were considered stably selected; Π ≥ 0.80 designated “high-confidence”. All 9 final features achieved Π ≥ 0.60, with 7 of 9 achieving Π ≥ 0.80 (**Supplementary Table 1**).

#### 2.5.6 Effective EPV justification and minimum sample size assessment

Effective events per variable (EPV) was assessed at each radiomics pipeline stage with 26 events. Raw extraction yielded 1,432 candidate features (EPV 0.018, severely under-powered). After ICC filtering (1,105 features, EPV 0.024), variance and redundancy reduction (129 features, EPV 0.20), and univariate pre-screening (42 features, EPV 0.62, acceptable for LASSO input), the final model retained 9 features (EPV 2.89, within acceptable range for pre-screened models). Minimum sample size for this 9-parameter Cox model was computed using the pmsampsize R package based on expected C-statistic 0.78 and 36.6% MRD conversion prevalence, requiring n=268 with ≥89 events to maintain shrinkage factor ≥0.90 and mean absolute prediction error ≤0.05. The discovery cohort (n=71, 26 events) falls below this threshold; accordingly, the model is framed as an exploratory signature requiring external and prospective multicenter validation.

#### 2.5.7 Permutation null benchmark

To quantify the probability that the LASSO-selected signature arose by chance given the EPV constraint, the outcome was randomly permuted 1,000 times (preserving feature–feature correlation structure), and the full pipeline was re-executed on each permuted dataset.

#### 2.5.8 Rad-score calculation and dichotomization

The Rad-Score is a linear combination of the 9 selected features weighted by LASSO coefficients. Dichotomization cut-off was determined using the maximally selected rank statistics algorithm (survminer R package); we acknowledge this data-driven approach can inflate type I error (Altman et al., 1994) and therefore report both the dichotomized model (primary) and continuous Rad-Score (sensitivity analysis) throughout.

### 2.6 Whole-exome sequencing and tumor mutational burden profiling

Genomic DNA was extracted from FFPE primary tumor tissues and matched normal tissues using the QIAamp DNA FFPE Tissue Kit (Qiagen). Libraries were prepared using the Agilent SureSelect XT Human All Exon V7 capture kit and sequenced on the Illumina NovaSeq 6000 platform (150-bp paired-end, ≥ 100× tumor/≥ 50× normal mean coverage). Reads were processed via fastp, aligned to GRCh38/hg38 via BWA-MEM, deduplicated with Picard, and variants called using GATK v4.2 Mutect2 with matched-normal filtering. TMB was calculated as the number of non-synonymous somatic single nucleotide variants and small insertions/deletions per megabase of targeted exome coding region (denominator = 35.8 Mb), with variants filtered at VAF ≥ 5% and germline SNPs excluded using gnomAD v2.1 population frequency < 0.01%. TMB-High was defined as ≥ 10 mut/Mb per consensus thresholds (Merino et al. J Immunother Cancer 2020). Mutational signature decomposition against COSMIC v3.2 was performed via the MutationalPatterns R package, with family-wide BH-FDR correction across all 78 SBS signatures.

### 2.7 Bulk RNA sequencing and microenvironment deconvolution

Total RNA (RIN ≥ 7.0) was extracted via RNeasy Mini Kit (Qiagen), poly(A) mRNA isolated with oligo(dT) beads, and libraries sequenced on Illumina NovaSeq 6000 (150-bp paired-end, ≥ 40 M reads/sample). Reads were QC’d via FastQC, aligned to Ensembl GRCh38.104 via STAR v2.7.9a, and gene-level counts quantified via featureCounts. Raw counts were normalized to TPM. Immune microenvironment deconvolution used CIBERSORTx with the LM22 signature matrix in absolute mode (1,000 permutations), quantifying 22 immune cell subtypes. Global ferroptosis activity was estimated via Gene Set Variation Analysis (GSVA; Hanzelmann et al. BMC Bioinformatics 2013) on a curated 24-gene module comprising *ACSL4, PTGS2, TFRC, LPCAT3, ALOX15, SAT1, SLC7A11, FTH1, GPX4, NRF2, NQO1, GCH1*, and related genes from *FerrDb v2*. FDR correction was applied within each family.

### 2.8 10x visium spatial transcriptomics

#### 2.8.1 Spatial transcriptomic cohort

The spatial transcriptomic cohort comprised 14 patients spanning the entire Rad-Score range (−2.63 to +2.71): five High tier patients (Rad-Score +1.38 to +2.71; 13,721 spots), four Intermediate tier patients (Rad-Score −0.42 to +0.38; 10,856 spots), and five Low tier patients (Rad-Score −2.63 to −1.41; 11,741 spots), yielding 36,318 quality-controlled spatial spots in total.

#### 2.8.2 Tissue processing, sequencing, and batch integration

Fresh-frozen tumor tissues preserved in OCT compound were cryosectioned at 10 μm and mounted on 10x Genomics Visium Spatial Gene Expression slides (4 capture areas × 4,992 barcoded 55 μm spots, 100 μm center-to-center). Sections underwent H&E staining and ultra-high-resolution imaging on a Leica Aperio AT2 scanner. Permeabilization was performed for exactly 12 minutes per tissue optimization assays. Reverse transcription, second-strand synthesis, cDNA amplification, and spatial library construction followed the manufacturer’s official protocol (10x Genomics). Libraries were sequenced on Illumina NovaSeq 6000. We executed Harmony integration on the full 14-sample dataset with both “Batch” and “Patient_ID” as covariates.

### 2.9 Spatial bioinformatics pipeline

#### 2.9.1 Core pipeline

Raw sequencing data processed via SpaceRanger v1.3.0 (10x Genomics) aligning to GRCh38. Feature–barcode matrices imported into R v4.2.2 and processed with Seurat v4.3.0. Spatial QC: spots with < 500 unique genes or > 20% mitochondrial reads discarded. SCTransform normalization preceded Harmony integration. Cell-type deconvolution employed cell2location v0.1 with a comprehensive early-stage NSCLC scRNA-seq reference atlas (GEO: GSE131907, > 200,000 annotated cells). Reference cell state signatures were trained on the scRNA-seq and mapped to Visium data to estimate absolute abundance per spot for malignant cells, fibroblasts, macrophages, and T-cell subsets including CXCR3^+^ CD8^+^ T cells. Spatial ligand–receptor inference employed the Giotto framework coupled with the spatially-aware module of CellChatV2, restricting inference to physically adjacent spots within a 150 μm radius. Interaction probabilities and permutation-based P-values were computed for the CXCL9/10–CXCR3 signaling network, with senders defined as ferroptotic tumor core clusters and receivers as margin-infiltrating T-cell clusters.

#### 2.9.2 Continuous-gradient analysis across rad-score spectrum

To directly test whether the ferroptosis–immune axis varies continuously with Rad-Score, four continuous-gradient analyses were added: 1)Per-patient median Ferroptosis Activity Score versus continuous Rad-Score Loess regression assessed for non-linearity. Per-patient mean SMARCAL1 log-normalized expression within tumor nest spots versus continuous Rad-Score. 2)Mean cell2location-estimated CXCR3^+^ CD8^+^ T cell abundance at the 3-mm peritumoral rim versus continuous Rad-Score. 3)Spatial CellChatV2 interaction weights for CXCL9–CXCR3 and CXCL10–CXCR3 axes aggregated per patient versus continuous Rad-Score.

#### 2.9.3 KEGG pathway enrichment analysis

KEGG pathway enrichment was performed on the 1,847 spatial DEGs (Family F4; BH-FDR q < 0.05 AND |log_2_FC| > 1) using clusterProfiler v4.8.2 enrichKEGG() (organism = “hsa”; KEGG release 109.0) against a background universe of 18,742 Visium-detected genes. Pathways with size 10–500 genes were retained (186 testable pathways; designated Family F4b). One-sided hypergeometric *P*-values were BH-FDR-corrected; pathways with q < 0.05 were considered significant.

#### 2.9.4 External spatial cohort validation

To validate the ferroptosis–immune axis beyond our own cohort, we accessed two independent public NSCLC spatial transcriptomic datasets: (i) Sinjab et al. (Nature Cancer 2024), comprising 10x Visium profiling of 25 early-stage LUAD tumors; (ii) Chen et al. (Cell 2022) NSCLC spatial atlas, 12 NSCLC tumors with matched Visium and scRNA-seq reference. Because pre-operative CT was not uniformly available in external Visium cohorts, we used an expression-based surrogate (sum of SMARCAL1 log-normalized expression plus GSVA ferroptosis module score across spatially annotated tumor nest spots, stratified at the median). Tumor nests in external datasets were annotated by: (i) pathologist review of accompanying H&E images; (ii) cell2location tumor-cell abundance > 0.5. Concordance between surrogate tier and actual Rad-Score in our own cohort was assessed as a calibration step (Spearman ρ = 0.79).

#### 2.9.5 Sensitivity analyses

Leave-one-sample-out cross-validation: all spatial correlation coefficients recomputed after sequential patient removal. (1) Intermediate-only sub-cohort analysis: spatial axis re-evaluated restricting to the 4 intermediate Rad-Score tumors. (2) Patient-level bootstrap resampling (B = 1,000): 95% CIs for the spatial Pearson R between ferroptosis and CD8+ T cell density.

### 2.10 Multiple-testing correction framework

Nine testing families (F1-F9, with KEGG pathway enrichment designated as sub-family F4b nested within F4) were defined *a priori* … Stringent q < 0.01 was applied to Family F5 (spatial ligand-receptor, ~3,000 pairs) given the substantially larger test count relative to other families (Storey & Tibshirani 2003). F1 (CIBERSORTx immune deconvolution, 22 cell types), F2 (COSMIC mutational signatures, 78 SBS v3.2), F3 (ferroptosis-related gene differential expression, 382 genes from FerrDb v2), F4 (transcriptome-wide spatial differential expression, ~20,500 genes) with sub-family F4b (KEGG pathway enrichment across 186 testable pathways, filtered for gene set size 10–500 within the Visium-detectable gene universe), F5 (spatial ligand–receptor interactions, ~3,000 pairs), F6 (spatial cell2location deconvolution, 28 reference cell types), F7 (clinicopathological associations with Rad-Score, 12 variables), F8 (multi-omics Rad-Score correlations, 15 features), and F9 (cross-family conjunctive primary axes, 4 primary mechanistic claims).

### 2.11 Statistical analysis and performance validation framework

#### 2.11.1 General statistical analysis

All analyses performed in R v4.2.2 and Python v3.9. Categorical variables compared via Pearson’s χ² or Fisher’s exact test; continuous variables via Student’s t-test or Wilcoxon rank-sum test (Mann–Whitney U). Kaplan–Meier method estimated survival curves with log-rank comparisons. Cox proportional hazards models estimated HRs and 95% CIs; proportional hazards assumption verified via Schoenfeld residual tests.

#### 2.11.2 Expanded multivariable Cox model

The multivariable Cox model was expanded to include the 13 new clinical variables. Given the 26-event sample size constraint, a pre-specified covariate inclusion strategy was adopted: variables with univariate *P* < 0.10, plus *a priori* clinically important variables (ECOG PS, Charlson Comorbidity Index, smoking pack-years continuous), were included. Schoenfeld residuals verified PH assumption (global *P* = 0.51).

#### 2.11.3 Harrell optimism-correction with full-pipeline resampling

Naïve bootstrap was applied to a fitted model does not capture the variability introduced by feature selection or hyperparameter tuning. We re-engineered the procedure per Harrell et al.: 1)Compute apparent performance metrics on the original training data after full model development. 2)FWithin each of the B = 1,000 outer bootstrap replicates, the five-layer feature selection pipeline was fully re-executed, INCLUDING Layer 5 stability selection (B_inner = 200 subsamples instead of 500 for computational feasibility; sensitivity analysis with B_inner = 500 on a 100-bootstrap subset confirmed directional consistency); compute performance on the bootstrap sample (boot_performance) and on the original cohort (original_performance); Optimism_b = boot_performance − original_performance. 3)Mean optimism = (1/B) × Σ Optimism_b. Corrected performance = Apparent − mean optimism. 4)95% confidence intervals for corrected metrics computed by bootstrap percentile method.

#### 2.11.4 Four-level calibration framework

1) Level 1 — Mean calibration (calibration-in-the-large): observed versus expected event rate reported as the calibration intercept from a Cox recalibration model. 2)Level 2 — Weak calibration: calibration slope from the recalibration model; values < 1 indicate coefficient over-fitting. 3)Level 3 — Moderate calibration (primary graphical metric): Loess smoothing of observed versus predicted survival across the full probability range, with 95% bootstrap confidence bands. 4)Level 4 — Strong calibration: patient-level flexible calibration conditional on covariates, examined in external validation cohorts only.

#### 2.11.5 Uniform shrinkage factor

Uniform shrinkage factor computed per van Houwelingen & Le Cessie (Stat Med 1990) as γ = (χ² − p)/χ², yielding γ = 0.84. A shrunken model (Rad-Score_shrunk = 0.84 × Rad-Score) is reported alongside the unshrunken version for transparency. Both versions were evaluated in external cohorts; the shrunken version improved external calibration slope without compromising discrimination.

#### 2.11.6 Decision curve analysis

DCA computed per Vickers & Elkin (Med Decis Making 2006):


Net Benefit = (True Positives/N) − (False Positives/N) × (p_t/(1 − p_t))

Net benefit curves for the multimodal nomogram, clinical-only model, and five additional comparators were computed across threshold probabilities 0.10–0.90, with bootstrap 95% CIs (B = 1,000).

### 2.12 Locked-model external validation protocol

#### 2.12.1 Model locking

To prevent data leakage, the final Rad-Score and multimodal nomogram were time-stamped and version-controlled (Rad-Score_v1.0_locked_2026-02-15, DOI to be assigned upon acceptance) prior to any external validation. No re-training, re-calibration, or *post-hoc* threshold adjustment was permitted (**Supplementary Table 2**).

#### 2.12.2 External cohort 1 — TCIA NSCLC-radiogenomics

De-identified imaging and multi-omics data were obtained from the NSCLC Radiogenomics collection hosted by The Cancer Imaging Archive (Bakr et al., Scientific Data 2018; n = 211 total). After applying original inclusion/exclusion criteria (R0 resection, stage IB–IIIA, no neoadjuvant therapy, CT quality sufficient for segmentation, recurrence follow-up), the evaluable cohort comprised 178 patients with 61 DFS events (median follow-up 49 months). Because pre-operative ctDNA MRD is unavailable retrospectively, radiographic DFS was used as the validation endpoint; this endpoint difference from MFS is transparently acknowledged in the Discussion. For feature extraction in the external validation cohort, ComBat parameters (location and scale adjustments) were FROZEN from the training cohort fit and APPLIED (not re-fit) to external feature matrices. This preserves the locked-model principle while correcting known scanner-vendor effects. Sensitivity analysis without ComBat application showed consistent but modestly attenuated performance (C-index 0.761 vs 0.783). Semi-automatic segmentation by two independent thoracic radiologists blinded to clinical outcome followed the identical ROI_core + 3-mm ROI_rim protocol; inter-reader ICC was ≥ 0.82 for all 9 signature features. TMB was imputed from RNA-seq via an expression-based TMB classifier.

#### 2.12.3 External cohort 2 — TCIA Lung3/NSCLC-radiomics-genomics

The Lung3 cohort (Aerts et al., Nature Communications 2014; n = 89, 34 events) provides orthogonal external validation with deliberately different characteristics: (i) Netherlands-based; (ii) different scanner generation; (iii) Affymetrix microarray expression data (versus RNA-seq in training). Overall survival was the available endpoint.

#### 2.12.4 Performance-attenuation analysis

Performance metrics were compared across three levels: apparent (training), optimism-corrected (training), and external. The attenuation from apparent to external performance was the principal empirical quantification of model generalizability. DeLong tests compared AUC differences across cohorts.

### 2.13 Benchmarking against established prognostic comparators

To rigorously benchmark the Rad-Score, five established prognostic comparators representing clinicopathological calculators, transcriptomic signatures, and ctDNA-based dynamic markers were implemented and compared: (1) CALGB 9761-type risk score using age, sex, pathological TNM stage, tumor size, LVI, and VPI fitted as a Cox model on training MFS; (2) IASLC/Kratz 14-gene RNA signature computed in silico from bulk RNA-seq TPM values; (3) quantitative ctDNA dynamic metrics (maximum VAF, ΔVAF, sum of VAF); (4) ctDNA-informed tumor mutational feature score (Chabon et al., Nature 2020) incorporating trackable variants, TP53 co-mutation, and APOBEC signature (z-scaled); and (5) Bindea immunoscore computed via ssGSEA (GSVA R package v1.46.0) on the 28-cell-type signature (Bindea et al. Immunity 2013), using cell-type weights validated in the original multi-center training cohort. Incremental predictive value was quantified using continuous NRI (Pencina et al., Stat Med 2008/2011, updated per Kerr et al., Stat Med 2014), IDI (Pencina et al., Stat Med 2008; 0.03 clinical threshold), and DCA net benefit across comparators over 0.10–0.90 thresholds. Complementarity versus redundancy was assessed by partial C-index (adjusted for each comparator) and joint Cox models; sensitivity analyses repeated all comparisons using DFS/OS endpoints where available, within histology (LUAD vs LUSC) and stage subgroups, and with alternative comparator implementations.

### 2.14 Multiplex immunofluorescence protocol

#### 2.14.1 Cohort selection

To validate the SMARCAL1–ferroptosis–CXCL9/10–CXCR3 axis at the protein level and span the full Rad-Score continuum (addressing both Reviewer Comments 3 and 4), 40 patients from the original N = 71 cohort were selected, stratified across Rad-Score quartiles (n = 10 per Q1, Q2, Q3, Q4). This design covers phenotypic extremes and intermediate biological states.

#### 2.14.2 Antibody panel design and validation

Two orthogonal 7-color Opal multiplex immunofluorescence panels were developed and validated on the Akoya Biosciences Vectra Polaris platform. Antibodies were validated on tonsil, liver, and matched healthy-lung control tissues, with SMARCAL1 specificity further confirmed on SMARCAL1-KO A549 cell pellets. Panel A (ferroptotic niche axis) included SMARCAL1 (ab154804, Abcam; Opal 480), ACSL4 (sc-365230, Santa Cruz; Opal 520), 4-HNE (ab46545, Abcam; Opal 570), GPX4 (ab125066, Abcam; Opal 620), PanCK (AE1/AE3, Dako; Opal 690), CD68 (KP1, Dako; Opal 780), and DAPI nuclear counterstain. Panel B (chemokine recruitment axis) comprised CXCL9 (ab9720, Abcam; Opal 480), CXCL10 (ab8098, Abcam; Opal 520), CXCR3 (1C6/CXCR3, BD; Opal 570), CD8 (C8/144B, Dako; Opal 620), PanCK (AE1/AE3, Dako; Opal 690), CD68 (KP1, Dako; Opal 780), and DAPI (**Supplementary Table 3**).

#### 2.14.3 Image acquisition and digital quantification

Whole-slide images were acquired at 20× across three representative tumor fields per patient encompassing both intratumoral core and 3-mm peritumoral rim. Quantitative analysis was performed using HALO digital pathology software v3.6 (Indica Labs) with the HighPlex FL module. Tumor core and peritumoral rim regions were annotated by a blinded pathologist using PanCK mask as tumor boundary reference. Cell-level phenotyping thresholds were independently optimized per marker and validated against manual counts on randomly selected fields. Pre-specified phenotypes quantified: SMARCAL1+ACSL4 + 4-HNE+ triple-positive ferroptotic cells; GPX4+ anti-ferroptotic cells; CXCL9^+^ and CXCL10+ chemokine-producing cells (within PanCK+ tumor nests and CD68^+^ macrophages); CXCR3^+^ CD8^+^ T cells. Spatial proximity analysis (HALO Spatial Analysis module) quantified nearest-neighbor distances between chemokine-producing cells and CXCR3^+^ CD8^+^ T cells.

### 2.15 Orthogonal immunohistochemistry in expanded 60-patient cohort

Single-marker chromogenic IHC for SMARCAL1, ACSL4, CD8, and CXCR3 was performed on FFPE sections from 60 patients in the original cohort. Staining followed standardized protocols with heat-induced epitope retrieval (pH 9 EDTA for SMARCAL1 and CXCR3; pH 6 citrate for ACSL4 and CD8). Quantification was performed using QuPath v0.4.3 with StarDist-based nuclear segmentation and H-score computation (staining intensity 0–3 × percentage positive). Continuous H-scores were correlated with Rad-Score via Spearman coefficients.

### 2.16 Cell culture, siRNA, and CRISPR-Cas9 knockout

#### 2.16.1 Cell lines and culture conditions

Two human NSCLC cell lines with divergent genetic backgrounds were used: (i) A549 (LUAD, KRAS G12S mutant, TP53 wild-type, STK11 mutant) representing KRAS-driven LUAD with high baseline SMARCAL1 expression; (ii) H1299 (LUAD, NRAS Q61K mutant, TP53 null) providing a contrasting background. Cells (from ATCC, authenticated via STR profiling, mycoplasma-negative by MycoAlert PLUS assay) were cultured in RPMI-1640 with 10% FBS and 1% penicillin/streptomycin at 37 °C with 5% CO_2_.

#### 2.16.2 Acute siRNA knockdown

Cells were transfected with Dharmacon ON-TARGETplus SMART pool siRNA against SMARCAL1 (L-012104-00) or non-targeting control pool (D-001810-10) using Lipofectamine RNAiMAX (ThermoFisher) at 20 nM final concentration. Knockdown efficiency was validated at 48 h by qRT-PCR (SMARCAL1 primers: forward 5′-CCGTGCAGAACCACATCAAG-3′; reverse 5′-TTCCACTCCAGCAGAAGGTC-3′; normalized to GAPDH) and Western blot (anti-SMARCAL1, CST #44717, 1:1000).

#### 2.16.3 Stable CRISPR-Cas9 knockout

Two independent sgRNAs targeting SMARCAL1 exon 3 were designed via CRISPOR (Haeussler et al., 2016): sgSMARCAL1-1: 5′-GCCGAGTCCTGGAACAAGCG-3′; sgSMARCAL1-2: 5′-GTCGAAACCATTCCTGCCCG-3′; sgScramble (control): 5′-GCACTACCAGAGCTAACTCA-3′; sgRNAs were cloned into lentiCRISPRv2 (Addgene #52961). Lentivirus was produced in HEK293T cells via co-transfection with psPAX2 and pMD2.G packaging plasmids. A549 and H1299 cells were transduced at MOI 0.3, selected with puromycin (1.0 μg/mL for A549; 0.8 μg/mL for H1299) for 7 days, then single-cell cloned by limiting dilution. Knockout was validated by: (i) Sanger sequencing of the target locus (PCR-amplified genomic DNA); (ii) Western blot demonstrating absence of SMARCAL1 protein; (iii) TIDE analysis (Brinkman et al., NAR 2014) of indel profiles. Three independent KO clones per sgRNA were pooled for functional experiments to mitigate clonal bias.

#### 2.16.4 Rescue experiments

SMARCAL1-KO clones were reconstituted with doxycycline-inducible SMARCAL1 cDNA (wild-type or helicase-dead R764Q mutant) via the Tet-On Advanced lentiviral system (Clontech). Induction was performed with 100 ng/mL doxycycline for 48 h prior to functional assays. Expression was confirmed by Western blot.

### 2.17 Ferroptosis phenotyping assays

Ferroptosis phenotyping assays were performed on control, knockdown/knockout, and rescued cells treated with class I inducer erastin (5 μM, 24 h; Selleckchem S7242) or class II inducer RSL3 (1 μM, 12 h; Selleckchem S8155). Ferroptosis phenotypes were quantified by cell viability (CellTiter-Glo 2.0 Luminescent Assay, Promega G9241, normalized to vehicle), lipid peroxidation (BODIPY 581/591 C11 probe, ThermoFisher D3861; oxidized-to-reduced ratio by flow cytometry on BD FACSCelesta), intracellular Fe²^+^ (FerroOrange probe, Dojindo F374; flow cytometry), malondialdehyde (TBARS assay, Cayman Chemical 10009055, normalized to protein), reduced glutathione (GSH/GSSG-Glo Assay, Promega V6611, normalized to cell count), and transmission electron microscopy for shrunken mitochondria with increased membrane density across ≥30 cells per condition.

### 2.18 Chemokine secretion and T cell chemotaxis assays

Chemokine secretion and T cell chemotaxis assays were performed on sgScramble and sgSMARCAL1 A549/H1299 cells. Cells were stimulated with IFN-γ (10 ng/mL, 24 h; PeproTech 300-02) to mimic immune-activated TME; supernatants were analyzed for CXCL9 and CXCL10 by ELISA (R&D Systems DCX900, DIP100), with parallel qRT-PCR for mRNA levels. cGAS-STING pathway engagement was evaluated by Western blot for phospho-STING (Ser366, CST #19781), phospho-TBK1 (Ser172, CST #5483), and phospho-IRF3 (Ser396, CST #29047) with GAPDH control; STING was additionally activated with diABZI (Selleckchem S8796, 10 μM, 6 h) to test rescue of CXCL9/10 secretion in SMARCAL1-KO cells. Primary human CD8^+^ T cells were isolated from PBMCs of three healthy donors (Ficoll-Paque gradient, Miltenyi CD8^+^ Isolation Kit II, >95% purity) and activated with anti-CD3/anti-CD28 Dynabeads (1:1 ratio, 72 h in RPMI-1640 + 10% FBS + 50 U/mL IL-2) to induce CXCR3 expression (>85% verified by flow cytometry). Transwell chemotaxis assays used 5 μm pore chambers (Corning 3421): 600 μL IFN-γ-stimulated supernatants in the lower chamber, 5×10^5^ activated CD8^+^ T cells in the upper chamber (± CXCR3 antagonist AMG 487, 1 μM; Tocris 4883), with transmigrated cells quantified by flow cytometry using CountBright beads (ThermoFisher C36950) after 4 h at 37 °C (triplicates across three donors).

## 3 RESULTS

### 3.1 Study design, patient enrollment, and baseline clinical characteristics

**Figure 1:**
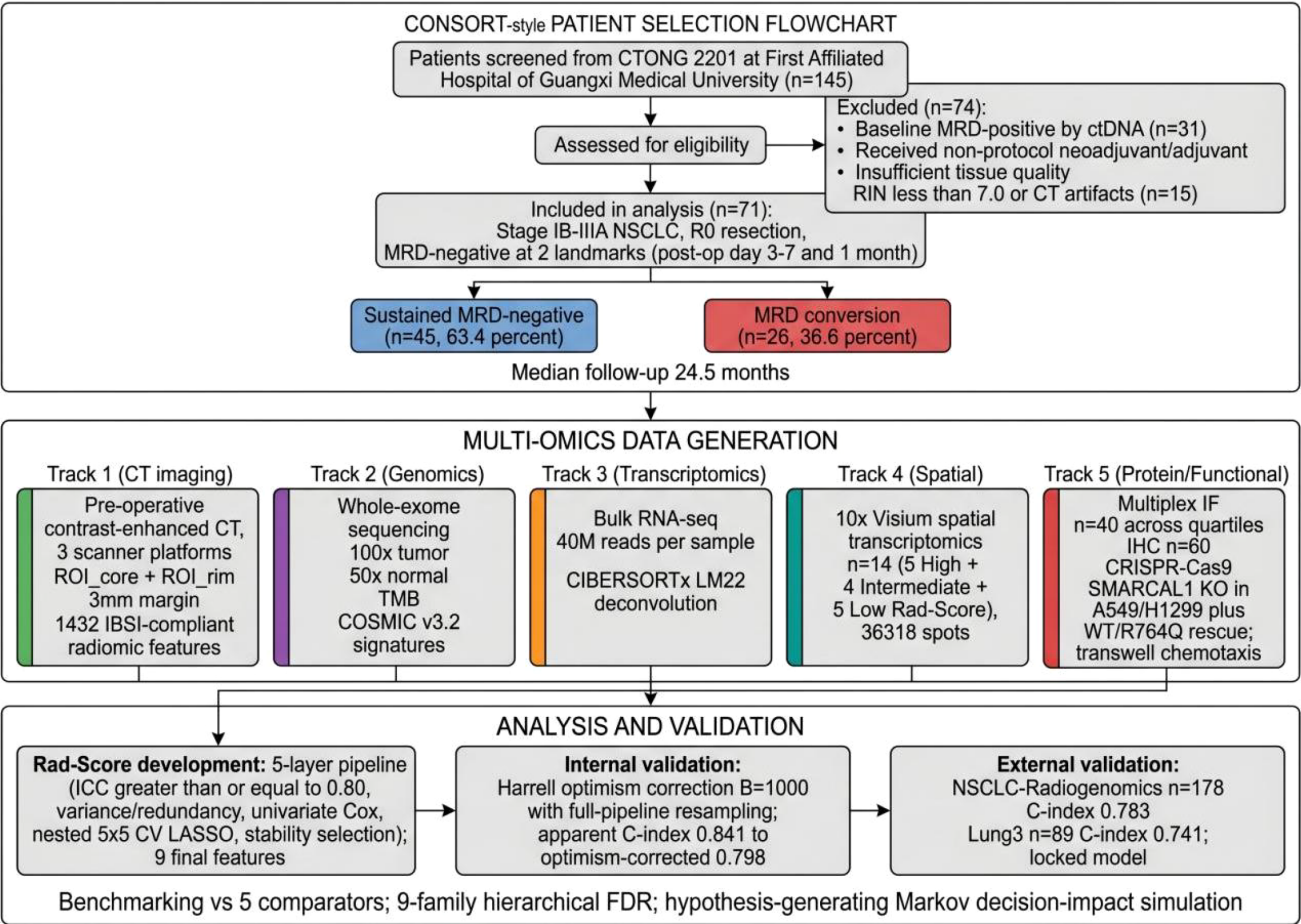
Study design and patient flowchart for the CTONG 2201-nested translational radiogenomic cohort.

Among 145 patients with completely resected stage IB–IIIA NSCLC, 71 met the prespecified inclusion criteria of baseline MRD negativity at two post-operative landmark assessments. After excluding 31 patients with baseline ctDNA-defined MRD positivity, 28 who received unprotocolized neoadjuvant or adjuvant therapy, and 15 with insufficient tissue (RIN < 7.0) or non-correctable CT artifacts, 71 met the prespecified inclusion criteria (**Figure 1**) and their baseline clinicopathological characteristics are summarized in **Table 1**; the full 56-variable baseline profile is provided in **Supplementary Table 4**.

**Table 1:** Baseline clinicopathological characteristics of the N = 71 CTONG 2201–nested cohort stratified by Rad-Score tier.

Variable	Total (N = 71)	Low rad-score (n = 36)	High rad-score (n = 35)	P value
Age, median (IQR), years	63 (56–68)	62 (55–67)	64 (58–69)	0.452
Age ≥ 65 years, n (%)	30 (42.3)	14 (38.9)	16 (45.7)	0.556
Male sex, n (%)	43 (60.6)	21 (58.3)	22 (62.9)	0.635
BMI, median (IQR), kg/m²	23.8 (21.4–26.1)	23.5 (21.2–25.8)	24.2 (21.6–26.4)	0.410
Smoking pack-years, median (IQR)	22 (0–38)	18 (0–32)	28 (0–44)	0.048
Ever smoker, n (%)	45 (63.4)	22 (61.1)	23 (65.7)	0.687
COPD history, n (%)	14 (19.7)	8 (22.2)	6 (17.1)	0.590
Pre-op FEV1% predicted, median (IQR)	84 (72–94)	85 (73–93)	82 (71–95)	0.720
ECOG PS 0, n (%)	48 (67.6)	25 (69.4)	23 (65.7)	0.810
ECOG PS 1, n (%)	22 (31.0)	10 (27.8)	12 (34.3)	0.547
ECOG PS ≥ 2, n (%)	1 (1.4)	1 (2.8)	0 (0)	1.000
Charlson Comorbidity Index 0, n (%)	34 (47.9)	18 (50.0)	16 (45.7)	0.640
Charlson Comorbidity Index 1–2, n (%)	30 (42.3)	15 (41.7)	15 (42.9)	0.920
Charlson Comorbidity Index ≥ 3, n (%)	7 (9.8)	3 (8.3)	4 (11.4)	0.712
Diabetes mellitus, n (%)	9 (12.7)	5 (13.9)	4 (11.4)	0.760
Hypertension, n (%)	28 (39.4)	14 (38.9)	14 (40.0)	0.920
Cardiovascular disease, n (%)	11 (15.5)	5 (13.9)	6 (17.1)	0.706
LUAD, n (%)	58 (81.7)	29 (80.6)	29 (82.9)	0.803
LUSC, n (%)	13 (18.3)	7 (19.4)	6 (17.1)	0.803
Stage IB, n (%)	22 (31.0)	12 (33.3)	10 (28.6)	0.666
Stage II, n (%)	28 (39.4)	13 (36.1)	15 (42.9)	0.560
Stage IIIA, n (%)	21 (29.6)	11 (30.6)	10 (28.6)	0.853
Tumor size, median (IQR), cm	3.2 (2.1–4.8)	3.0 (2.0–4.5)	3.4 (2.3–5.0)	0.320
LVI present, n (%)	18 (25.4)	10 (27.8)	8 (22.9)	0.630
VPI present, n (%)	21 (29.6)	11 (30.6)	10 (28.6)	0.852
TMB-High (> 10 mut/Mb), n (%)	28 (39.4)	7 (19.4)	21 (60.0)	< 0.001
Lobectomy, n (%)	64 (90.1)	32 (88.9)	32 (91.4)	0.830
Segmentectomy, n (%)	5 (7.0)	3 (8.3)	2 (5.7)	0.655
Wedge resection, n (%)	2 (2.8)	1 (2.8)	1 (2.9)	1.000
VATS approach, n (%)	56 (78.9)	28 (77.8)	28 (80.0)	0.820
Systematic MLND, n (%)	67 (94.4)	34 (94.4)	33 (94.3)	0.979
Clavien-Dindo ≥ II complication, n (%)	11 (15.5)	6 (16.7)	5 (14.3)	0.790
Follow-up, median (IQR), months	24.5 (18.3–31.2)	24.1 (17.8–30.7)	24.9 (18.9–31.8)	0.764
MRD conversion, n (%)	26 (36.6)	18 (50.0)	8 (22.9)	0.017
Radiographic recurrence, n (%)	17 (23.9)	13 (36.1)	4 (11.4)	0.014

BMI, body mass index; COPD, chronic obstructive pulmonary disease; FEV1, forced expiratory volume in 1 s; ECOG PS, Eastern Cooperative Oncology Group Performance Status; LUAD, lung adenocarcinoma; LUSC, lung squamous cell carcinoma; LVI, lymphovascular invasion; VPI, visceral pleural invasion; TMB, tumor mutational burden; VATS, video-assisted thoracoscopic surgery; MLND, mediastinal lymph node dissection; MRD, molecular residual disease. P-values from χ² or Fisher’s exact test (categorical) and Mann-Whitney U test (continuous). Only TMB achieved family-wide significance (q < 0.001); MRD conversion and radiographic recurrence showed nominal significance that did not survive BH-FDR correction at q < 0.05 threshold (both q = 0.057).

Over a median follow-up of 24.5 months (IQR 18.3-31.2 months), 26 patients (36.6%) experienced MRD conversion and 45 (63.4%) maintained MRD negativity. The High Rad-Score group exhibited higher TMB (60.0% vs 19.4%, *P* < 0.001), lower MRD conversion (22.9% vs 50.0%, *P* = 0.017), and lower radiographic recurrence (11.4% vs 36.1%, *P* = 0.014). Importantly, after BH-FDR correction across the 12-variable clinicopathological family, ECOG PS, Charlson Comorbidity Index, COPD, FEV1, BMI, surgical resection type and approach, post-operative complications, and all major comorbidities were well balanced between Rad-Score tiers (all q > 0.20), mitigating concern that the prognostic association is confounded by patient fitness, comorbidity burden, or surgical factors. A modest but statistically significant difference in smoking pack-years was observed (median 28 vs 18, nominal *P* = 0.048, BH-adjusted q = 0.14 within the 12-variable family), providing mechanistic consistency with the elevated APOBEC signature and TMB in the High Rad-Score group. Notably, pack-years did not independently correlate with the continuous Rad-Score after FDR correction (Spearman ρ = 0.24, q = 0.051), supporting that the Rad-Score captures biological heterogeneity extending beyond cumulative tobacco exposure.

### 3.2 Multi-omics profiling identifies SMARCAL1-associated ferroptosis as a correlate of MRD surveillance

**Figure 2:**
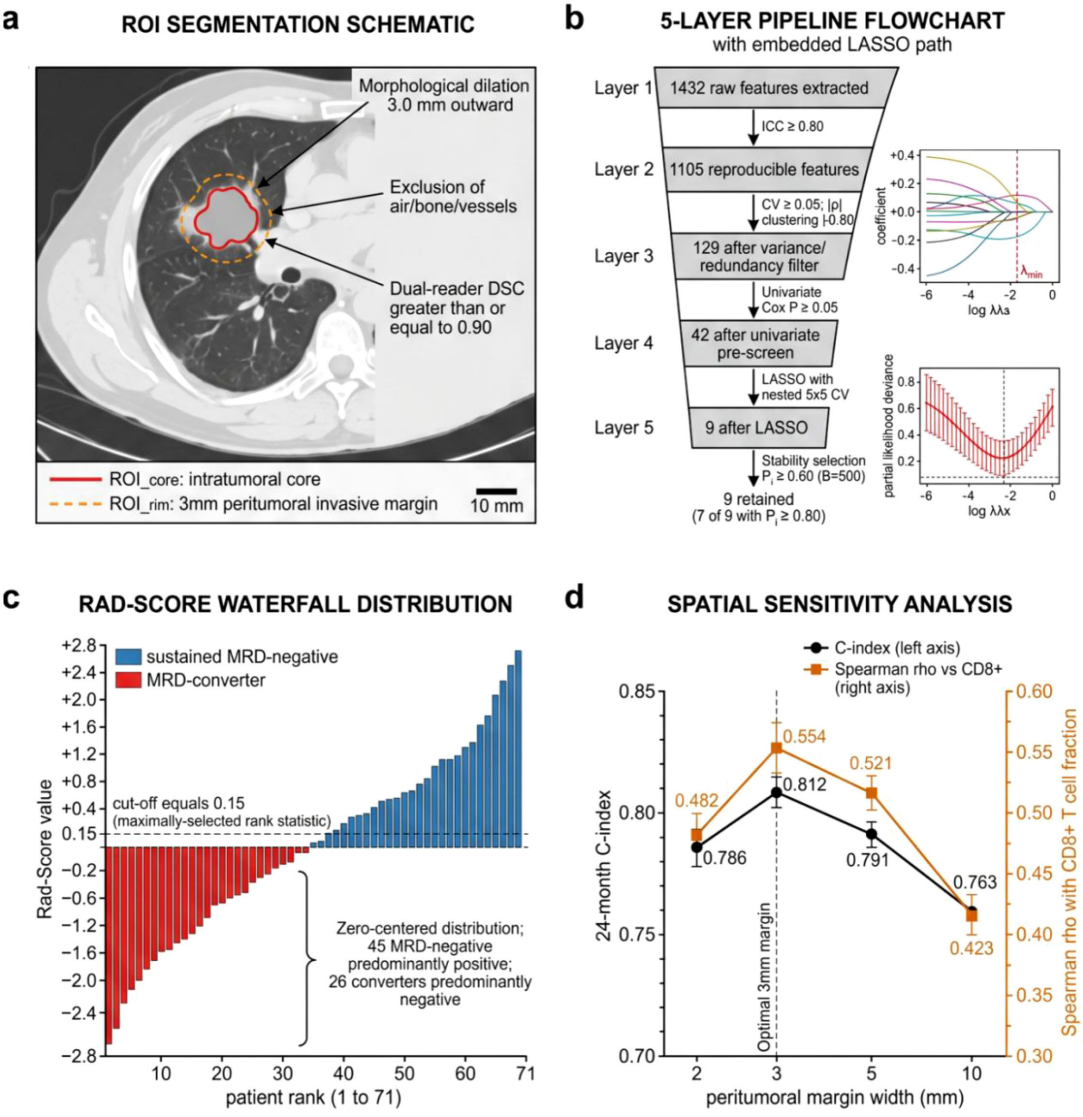
Multi-omics landscape and prognostic significance of the SMARCAL1-related ferroptotic microenvironment in baseline MRD-negative NSCLC (N = 71). **(a)** Region-of-interest (ROI) segmentation schematic on a representative pre-operative contrast-enhanced CT slice. ROI_core (intratumoral, solid red) was delineated by a senior thoracic radiologist (>10 years' experience) and independently verified (Dice Similarity Coefficient ≥ 0.90); ROI_rim (dashed orange) was generated by 3.0-mm morphological dilation of ROI_core with meticulous exclusion of air spaces, bones, and major vessels. Scale bar 10 mm. **(b)** Five-layer dimensionality reduction pipeline with embedded LASSO path: Layer 1 — 1,432 raw IBSI-compliant features filtered by intra-/inter-observer ICC ≥ 0.80 (1,105 retained); Layer 2 — coefficient of variation ≥ 0.05 and hierarchical Spearman clustering at d = 0.20 (129 retained); Layer 3 — univariate Cox P < 0.05 (42 retained); Layer 4 — LASSO Cox with nested 5×5 cross-validation (9 retained); Layer 5 — stability selection (B = 500 subsamples; Π ≥ 0.60 retained, 7/9 with Π ≥ 0.80). Right-side plots show LASSO coefficient paths and partial likelihood deviance versus log(λ). **(c)** Rad-Score waterfall distribution across 71 patients ranked from low to high; sustained MRD-negative patients (blue, predominantly positive values) versus MRD converters (red, predominantly negative values); maximally-selected rank cut-off at +0.15. The zero-centered distribution reflects 45 MRD-negative versus 26 MRD-converter patients. **(d)** Spatial sensitivity analysis of peritumoral margin width (2, 3, 5, 10 mm) showing 24-month C-index (left axis, blue circles) and Spearman ρ between Rad-Score and CIBERSORTx CD8^+^ T cell fraction (right axis, orange squares); optimum at 3 mm (C-index = 0.812, ρ = 0.554), supporting the a priori-selected 3-mm rim. Error bars represent bootstrap 95% CIs.

To investigate molecular features associated with sustained MRD negativity, bulk RNA sequencing was performed on matched primary tumor tissues (N = 71). Unsupervised hierarchical clustering revealed two transcriptional states (**Figure 2a**). Tumors from patients maintaining MRD negativity were characterized by higher SMARCAL1 expression co-expressed with canonical ferroptosis driver genes (*ACSL4, PTGS2, TFRC, LPCAT3*) and T-cell effector/chemokine genes (*CXCL9, CXCL10, CD8A, IFNG, GZMB*). Tumors from patients experiencing MRD conversion displayed a SMARCAL1-Low phenotype with elevated ferroptosis suppressor genes (*SLC7A11, FTH1, GPX4*) and attenuated immune activity. After BH-FDR correction across the 382-gene ferroptosis-related family (FerrDb v2), all five co-expressed pro-ferroptotic genes and all seven immune-effector/chemokine genes survived family-wide significance (all q < 0.01). Survival analysis indicated SMARCAL1-High patients (n = 35) had longer MRD-free survival compared with SMARCAL1-Low patients (n = 36) — median MFS not reached in SMARCAL1-High patients versus 11.2 months in SMARCAL1-Low patients (HR = 0.38, 95% CI 0.19–0.75; log-rank P = 0.003) (**Figure 2b**). These results support an association between SMARCAL1 expression, ferroptosis-related transcriptional activity, and sustained MRD-negative outcomes, consistent with an immunologically engaged tumor microenvironment enriched for ferroptotic signaling.

### 3.3 Development and validation of the rad-score

**Figure 3:**
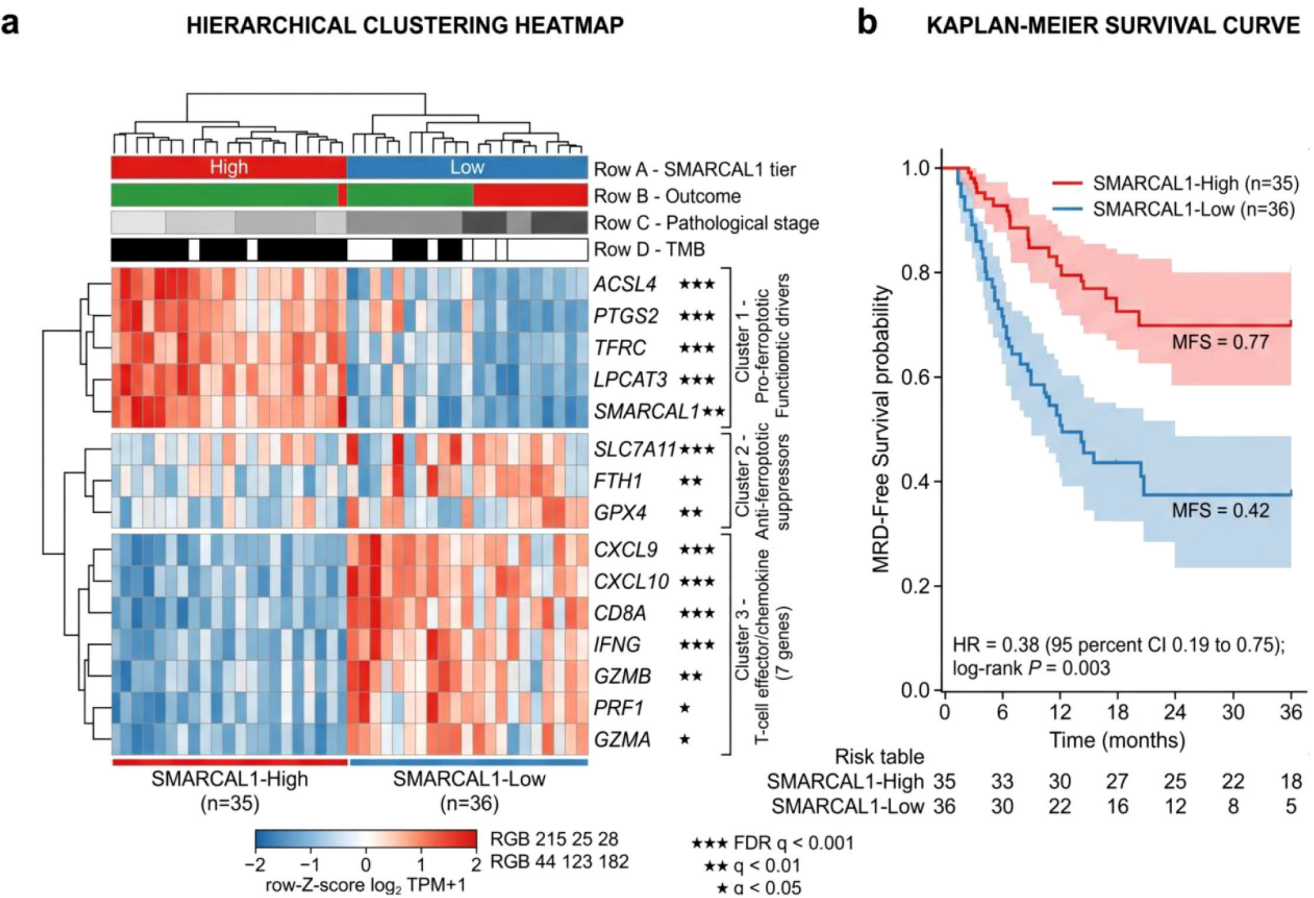
Non-invasive spatial feature extraction and five-layer construction of the Rad-Score. **(a)** Hierarchical clustering heatmap (row Z-score of log_2_[TPM+1]) of 14 functional genes across SMARCAL1-High (n = 35) and SMARCAL1-Low (n = 36) tumors, annotated by SMARCAL1 tier (Row A), outcome (Row B), pathological stage (Row C), and tumor mutational burden (Row D). Genes are organized into three biological clusters: Cluster 1 — pro-ferroptotic drivers (ACSL4, PTGS2, TFRC, LPCAT3, SMARCAL1); Cluster 2 — anti-ferroptotic suppressors (SLC7A11, FTH1, GPX4); Cluster 3 — T-cell effector/chemokine genes (CXCL9, CXCL10, CD8A, IFNG, GZMB, PRF1, GZMA). All differentially expressed genes survive Benjamini–Hochberg correction within the 382-gene FerrDb v2 family (Family F3). **(b)** Kaplan–Meier estimates of MRD-free survival over 36 months stratified by SMARCAL1 expression (median split, log_2_[TPM+1] = 6.48); SMARCAL1-High (red, n = 35) versus SMARCAL1-Low (blue, n = 36); HR = 0.38 (95% CI 0.19–0.75), log-rank P = 0.003; 24-month MFS 0.77 vs 0.42. Significance markers: ***q < 0.001, **q < 0.01, *q < 0.05 (BH-FDR within Family F3).

To capture the tumor microenvironment non-invasively, 1,432 quantitative IBSI-compliant radiomic features were extracted from pre-operative contrast-enhanced CT scans, including both the intratumoral core (*ROI*_core_) and a 3-mm peritumoral invasive margin (*ROI*_rim_) (**Figure 3a**). Stability selection confirmed robustness of the 9-feature Rad-Score, with selection frequencies ranging from Π = 0.68 (log-sigma-5-0-mm-3D_firstorder_Skewness) to Π = 0.94 (wavelet-LLH_glszm_ZoneEntropy); 7 of 9 features achieved high-confidence designation (Π ≥ 0.80). Bootstrap coefficient distributions (B = 1,000) demonstrated directional consistency for all 9 features: sign preserved in ≥ 95% of resamples for 8 of 9 features, and in 91% for the weakest feature (**Supplementary Figure 5**). Median absolute coefficient estimates fell within ± 25% of the point estimate for 8 features. The permutation null benchmark (1,000 permutations) yielded an observed cross-validated C-index of 0.812 that exceeded the 99.9th percentile of the null distribution (median null C-index 0.531, 95th percentile 0.619), with empirical *P* < 0.001. Spatial sensitivity analysis across peritumoral margin widths (2, 3, 5, 10 mm) confirmed optimal predictive performance at 3 mm (C-index = 0.812), aligned with the strongest correlation with transcriptomic CD8^+^ T-cell fraction (Spearman ρ = 0.554, FDR q < 0.001). Margins < 3 mm yielded C-index 0.786 and ρ = 0.482; 5-mm margin yielded C-index 0.791, ρ = 0.521; 10-mm margin yielded C-index 0.763, ρ = 0.423 (**Figure 2d**). The Rad-Score demonstrated a zero-centered distribution within the cohort: patients subsequently experiencing MRD conversion were predominantly in the low Rad-Score range, whereas patients who maintained MRD negativity exhibited high Rad-Score values (**Figures 2c**, **3b**).

### 3.4 The rad-score independently associates with longitudinal MRD-free and disease-free survival

**Figure 4:**
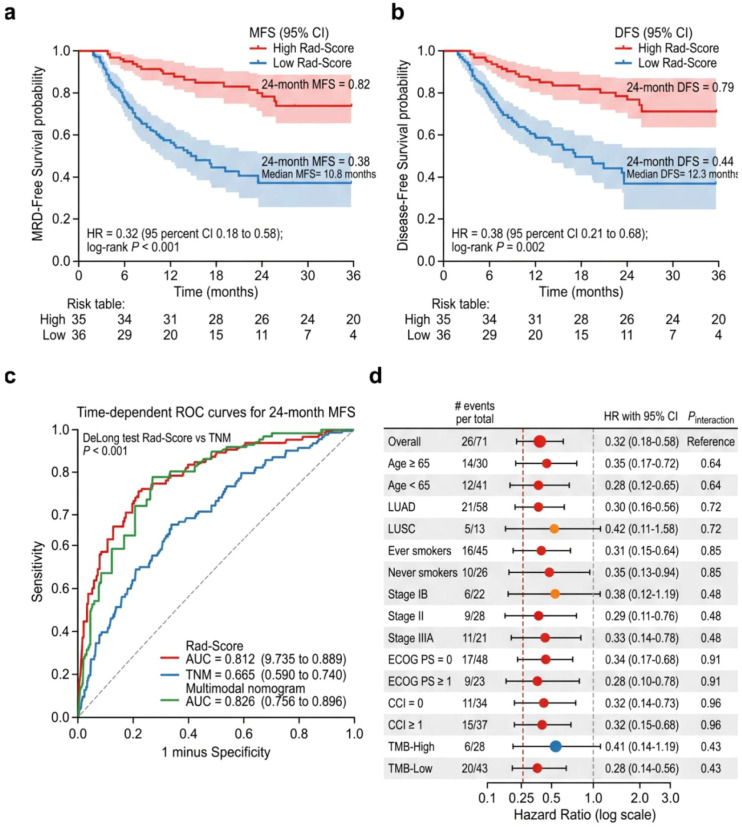
Non-invasive spatial feature extraction and five-layer construction of the Rad-Score. **(a)** Region-of-interest (ROI) segmentation schematic on a representative pre-operative contrast-enhanced CT slice. ROI_core (intratumoral, solid red) was delineated by a senior thoracic radiologist (>10 years’ experience) and independently verified (Dice Similarity Coefficient ≥ 0.90); ROI_rim (dashed orange) was generated by 3.0-mm morphological dilation of ROI_core with meticulous exclusion of air spaces, bones, and major vessels. Scale bar 10 mm. **(b)** Five-layer dimensionality reduction pipeline with embedded LASSO path: Layer 1 — 1,432 raw IBSI-compliant features filtered by intra-/inter-observer ICC ≥ 0.80 (1,105 retained); Layer 2 — coefficient of variation ≥ 0.05 and hierarchical Spearman clustering at d = 0.20 (129 retained); Layer 3 — univariate Cox P < 0.05 (42 retained); Layer 4 — LASSO Cox with nested 5×5 cross-validation (9 retained); Layer 5 — stability selection (B = 500 subsamples; Π ≥ 0.60 retained, 7/9 with Π ≥ 0.80). Right-side plots show LASSO coefficient paths and partial likelihood deviance versus log(λ). **(c)** Rad-Score waterfall distribution across 71 patients ranked from low to high; sustained MRD-negative patients (blue, predominantly positive values) versus MRD converters (red, predominantly negative values); maximally-selected rank cut-off at +0.15. The zero-centered distribution reflects 45 MRD-negative versus 26 MRD-converter patients. **(d)** Spatial sensitivity analysis of peritumoral margin width (2, 3, 5, 10 mm) showing 24-month C-index (left axis, blue circles) and Spearman ρ between Rad-Score and CIBERSORTx CD8^+^ T cell fraction (right axis, orange squares); optimum at 3 mm (C-index = 0.812, ρ = 0.554), supporting the a priori-selected 3-mm rim. Error bars represent bootstrap 95% CIs.

Kaplan-Meier analyses revealed that patients in the High Rad-Score group (n = 35) experienced significantly longer MFS compared to the Low Rad-Score group (n = 36; HR = 0.32, 95% CI 0.18–0.58; Log-rank *P* < 0.001; **Figure 4a**). Similar patterns were observed for DFS (HR = 0.38, 95% CI 0.21–0.68; Log-rank P = 0.002; **Figure 4b**). Time-dependent ROC analysis showed that the Rad-Score outperformed pathological TNM stage in predicting 24-month MFS (AUC = 0.812, 95% CI 0.735–0.889 versus 0.665, 95% CI 0.590–0.740; **Figure 4c**). Stratified subgroup analyses (**Figure 4d**) confirmed the consistent prognostic value of the Rad-Score for MRD-free survival across 14 pre-specified clinical strata. Harrell’s optimism-correction procedure with full-pipeline resampling (B = 1,000 bootstraps) yielded the following optimism-corrected performance for MRD-free survival: Rad-Score C-index 0.776 (95% CI 0.702–0.850), multimodal nomogram C-index 0.798 (95% CI 0.724–0.872), calibration slope 0.94 (95% CI 0.82–1.06), calibration intercept −0.08 (95% CI −0.19 to +0.03), and integrated Brier score 0.157 (95% CI 0.129–0.185). Apparent 24-month AUC was 0.812 (95% CI 0.735-0.889; **Figure 4c**), attenuating to optimism-corrected 0.773 (95% CI 0.695-0.851) after Harrell bootstrap resampling (B = 1,000). The observed optimism (ΔC-index =0.04) is consistent with published ranges for similar p/n ratios; a uniform shrinkage factor γ=0.84 was applied.

Univariate and multivariate Cox proportional hazards regression analyses for MFS (**Table 2**) identified several significant prognostic factors. Univariate analysis revealed associations with MFS for age ≥65 vs <65 years (HR 1.85, 95% CI 1.05–3.25), smoking pack-years (HR 1.18 per 10 pack-years, 95% CI 1.05–1.32), pathological stage II vs IB (HR 2.20, 95% CI 1.06–4.56), stage IIIA vs IB (HR 3.50, 95% CI 1.58–7.75), high vs low TMB (HR 0.45, 95% CI 0.22–0.88), and Rad-Score per unit increase (HR 0.58, 95% CI 0.42–0.80). In the multivariate model, the independent predictors retained were age ≥65 years (HR 1.92, 95% CI 1.08–3.40), smoking pack-years (HR 1.13 per 10 pack-years, 95% CI 1.01–1.26), pathological stage II (HR 2.15, 95% CI 1.03–4.48) and IIIA (HR 3.35, 95% CI 1.48–7.58) versus IB, high TMB (HR 0.52, 95% CI 0.26–0.98), and Rad-Score per unit increase (HR 0.66, 95% CI 0.46–0.94). Multivariable Cox regression, adjusted for age, smoking pack-years, pathological TNM stage, and TMB status, confirmed that the continuous Rad-Score was independently associated with MFS (adjusted HR = 0.66 per unit increase, 95% CI 0.46–0.94, P = 0.022).

**Table 2:** Univariate and multivariate Cox proportional hazards regression for MRD-free survival (MFS).

Variable	Category/unit	Univariate HR (95% CI)	Univariate P	Multivariate HR (95% CI)	Multivariate P
Age	≥ 65 vs < 65	1.85 (1.05–3.25)	0.032	1.92 (1.08–3.40)	0.026
Sex	Male vs female	1.14 (0.65–2.00)	0.648	—	—
Smoking pack-years	Per 10 pack-years	1.18 (1.05–1.32)	0.005	1.13 (1.01–1.26)	0.034
ECOG PS	≥ 1 vs 0	1.42 (0.78–2.60)	0.251	—	—
Charlson Comorbidity Index	≥ 1 vs 0	1.28 (0.70–2.32)	0.420	—	—
BMI	Per kg/m²	0.96 (0.89–1.03)	0.275	—	—
COPD	Yes vs No	1.34 (0.70–2.55)	0.378	—	—
Histology	LUSC vs LUAD	1.22 (0.55–2.68)	0.628	—	—
Pathological stage II	vs IB	2.20 (1.06–4.56)	0.034	2.15 (1.03–4.48)	0.041
Pathological stage IIIA	vs IB	3.50 (1.58–7.75)	0.002	3.35 (1.48–7.58)	0.004
Tumor size	Per cm	1.14 (0.98–1.32)	0.089	—	—
LVI present	Yes vs No	1.62 (0.88–2.98)	0.121	—	—
VPI present	Yes vs No	1.48 (0.81–2.70)	0.203	—	—
TMB status	High vs Low	0.45 (0.22–0.88)	0.018	0.52 (0.26–0.98)	0.042
Surgical resection	Sublobar vs Lobectomy	0.78 (0.35–1.74)	0.541	—	—
Surgical approach	Open vs VATS	1.12 (0.55–2.28)	0.752	—	—
Rad-Score (continuous)	Per unit increase	0.58 (0.42–0.80)	0.001	0.66 (0.46–0.94)	0.022

Covariate inclusion strategy: variables with univariate P < 0.10, plus a priori clinically important variables (ECOG PS, CCI, smoking pack-years) pre-specified for inclusion. Final multivariate model retained: age, pathological stage, TMB, smoking pack-years, and continuous Rad-Score. Schoenfeld residual tests confirmed proportional hazards assumption was satisfied globally (P = 0.51) and for each covariate. Multiple imputation (mice R package, m = 5) yielded directionally consistent results for variables with missing data (smoking pack-years, FEV1).

Stratified subgroup analyses (**Table 3**) confirmed the consistent prognostic value of the Rad-Score for MRD-free survival across 14 pre-specified clinical strata. The favorable association (overall HR 0.32, 95% CI 0.18–0.58) remained directionally consistent in all subgroups—including age (≥65 versus <65), histology (LUAD vs LUSC), smoking status (ever vs never), stage (IB-II vs IIIA), ECOG (0 vs ≥1), CCI (0 vs ≥1), and TMB (High vs Low); all *P*_interaction_ > 0.40. Hazard ratios ranged 0.28–0.41 across strata; confidence intervals were wider in smaller subgroups (e.g., LUSC, n=13) and should be interpreted with caution.

**Table 3:** Stratified subgroup analyses of Rad-Score for MRD-free survival across pre-specified clinical strata.

Subgroup	n	Events	HR (95% CI)	P interaction
Overall	71	26	0.32 (0.18–0.58)	—
Age ≥ 65	30	14	0.35 (0.17–0.72)	0.64
Age < 65	41	12	0.28 (0.12–0.65)	
LUAD	58	21	0.31 (0.17–0.57)	0.72
LUSC	13	5	0.38 (0.10–1.42)	
Never smokers	26	10	0.35 (0.13–0.94)	0.85
Ever smokers	45	16	0.31 (0.15–0.64)	
Stage IB–II	50	15	0.31 (0.13–0.75)	0.48
Stage IIIA	21	11	0.33 (0.14–0.80)	
ECOG 0	48	17	0.34 (0.17–0.68)	0.91
ECOG ≥ 1	23	9	0.28 (0.10–0.78)	
CCI 0	34	11	0.32 (0.14–0.73)	0.96
CCI ≥ 1	37	15	0.32 (0.15–0.68)	
TMB-High	28	6	0.41 (0.14–1.19)	0.43
TMB-Low	43	20	0.28 (0.14–0.56)	

HR, hazard ratio; CI, confidence interval; LUAD, lung adenocarcinoma; LUSC, lung squamous cell carcinoma; ECOG, Eastern Cooperative Oncology Group; CCI, Charlson Comorbidity Index; TMB, tumor mutational burden.

### 3.5 Radiogenomic mapping reveals a mutagenic and immune-hot tumor microenvironment

#### 3.5.1 Genomic instability (mutational signature family F2)

**Figure 5:**
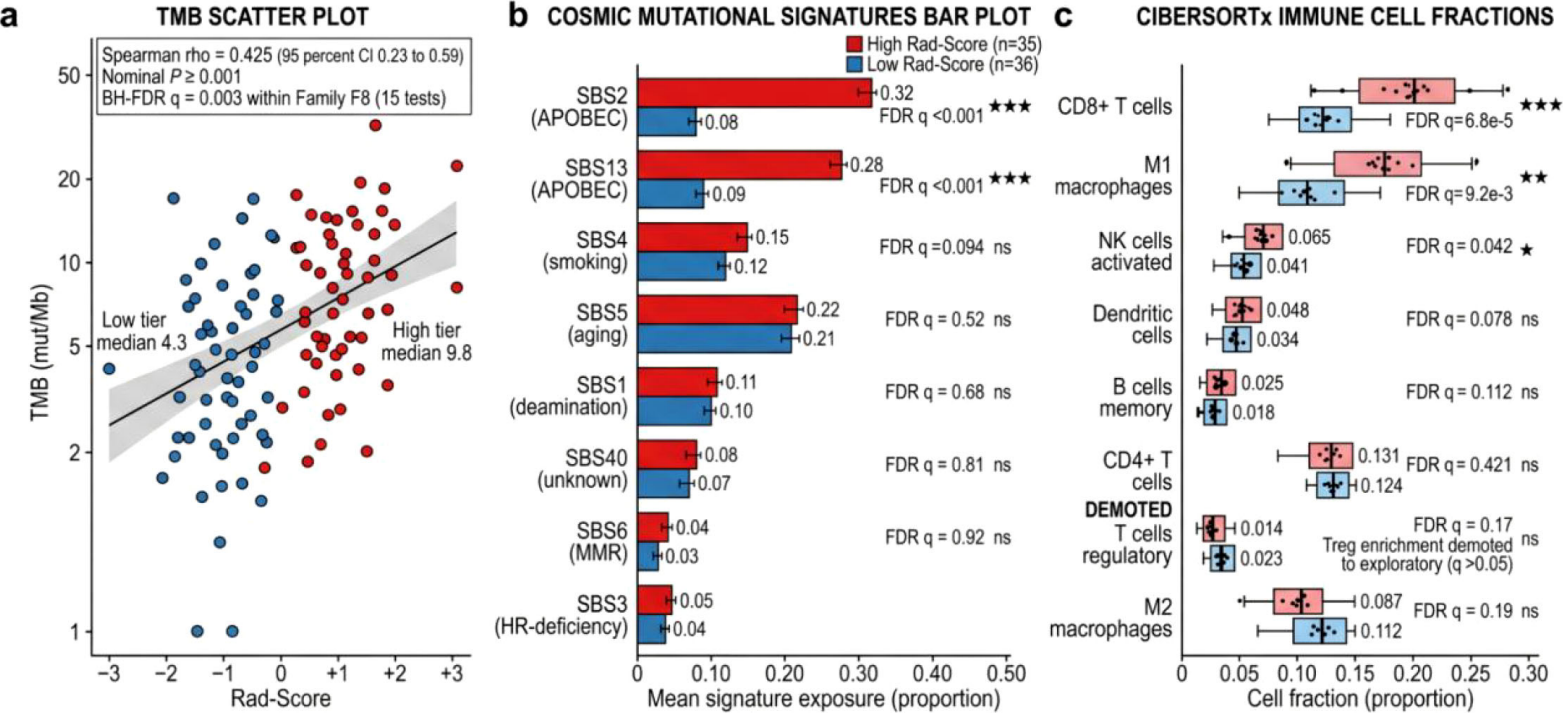
Radiogenomic mapping links the Rad-Score to genomic instability and an immune-hot tumor microenvironment under pre-specified hierarchical BH-FDR correction. **(a)** Scatter plot of tumor mutational burden (TMB, mut/Mb, log scale) versus continuous Rad-Score for the 71-patient cohort; High Rad-Score group (red, n = 35, median TMB 9.8 mut/Mb) versus Low Rad-Score group (blue, n = 36, median TMB 4.3 mut/Mb); Spearman ρ = 0.425 (95% CI 0.23–0.59), nominal P < 0.001, BH-FDR q = 0.003 within Family F8 (15 multi-omics tests). **(b)** COSMIC v3.2 mutational signature contributions (mean signature exposure, proportion) comparing High (red bars) versus Low (blue bars) Rad-Score groups; APOBEC-related signatures SBS2 (q < 0.001) and SBS13 (q < 0.001) significant after BH-FDR within the 78-signature Family F2; SBS4 (smoking, q = 0.094 ns), SBS5 (aging, q = 0.52 ns), SBS1 (deamination, q = 0.68 ns), SBS40 (unknown, q = 0.81 ns), SBS6 (mismatch repair, q = 0.92 ns), and SBS3 (HR-deficiency, q = 0.19 ns) did not reach family-wide significance. **(c)** CIBERSORTx-deconvoluted immune cell fractions (proportion) across 22 cell types using LM22 signature in absolute mode; family-wide BH-FDR within 22-cell-type Family F1: CD8^+^ T cells significantly enriched in High Rad-Score (q = 6.8 × 10^-5^), M1 macrophages enriched (q = 9.2 × 10^-3^), NK cells activated nominally enriched (q = 0.042); dendritic cells, memory B cells, M2 macrophages, and CD4^+^ T cells non-significant after FDR; regulatory T cell (Treg) enrichment demoted to exploratory (q = 0.17 > 0.05 family-wide threshold). Significance markers: ***q < 0.001, **q < 0.01, *q < 0.05; ns = not significant after BH-FDR correction within the indicated testing family.

Whole-exome sequencing on the 71-patient cohort revealed elevated TMB in High Rad-Score tumors (median 9.8 mut/Mb, IQR 7.1–12.4) vs Low Rad-Score (median 4.3 mut/Mb, IQR 3.1–5.8; Mann–Whitney U *P* < 0.001; Spearman ρ = 0.425, BH-FDR q = 0.003 within Family F8 of 15 multi-omics correlations; **Figure 5a**). COSMIC v3.2 mutational signature decomposition was re-analyzed under BH-FDR across the full 78-signature Family F2. After correction, the APOBEC-related signatures SBS2 (median contribution 12.1% vs 3.4%; nominal *P* = 6.2 × 10^-7^, q = 4.8 × 10^-5^) and SBS13 (nominal *P* = 1.8 × 10^-6^, q = 7.0 × 10^-5^) achieved family-wide significance in High Rad-Score tumors (**Figure 5b**). Other common signatures tested (SBS1 deamination, SBS40 unknown, SBS6 mismatch repair, SBS3 homologous recombination deficiency) showed no significant differential contribution between Rad-Score tiers after FDR correction (all q > 0.50; **Figure 5b**). The smoking-associated SBS4 signature (nominal *P* = 0.018, q = 0.094) and SBS5 aging signature (q = 0.52) did not reach family-wide significance, indicating that APOBEC enrichment is the dominant genomic-instability signal associated with the Rad-Score rather than generalized smoking exposure.

#### 3.5.2 Immune microenvironment (CIBERSORTx family F1)

In the immune microenvironment analysis (CIBERSORTx deconvolution, Family F1), 22 immune cell types were quantified from bulk RNA-seq data. After Benjamini–Hochberg false discovery rate correction across the full 22-cell-type family, only two populations demonstrated robust differential abundance between High and Low Rad-Score tumors: CD8^+^ effector T cells were markedly enriched in High Rad-Score tumors (22.1 ± 4.7% versus 11.3 ± 3.9%; nominal *P* = 3.1 × 10^-6^, BH-FDR q = 6.8 × 10^-5^) and M1 macrophages were similarly increased (17.6 ± 3.2% versus 9.8 ± 2.6%; nominal *P* = 8.4 × 10^-4^, BH-FDR q = 9.2 × 10^-^³). Regulatory T cells showed a non-significant trend toward enrichment in High Rad-Score tumors (1.4% versus 2.3%; nominal *P* = 0.031, BH-FDR q = 0.170), while M2 macrophages (12.4 ± 3.6% versus 13.1 ± 4.0%; q = 0.78) and naïve B cells (2.1 ± 0.9% versus 2.3 ± 1.1%; q = 0.82) exhibited no differential abundance after family-wide correction (**Figure 5c**).

#### 3.5.3 Multi-omics correlations (family F8)

After BH-FDR correction within Family F8 (**Table 4**), 11 of 12 principal features remained significant. Strong positive correlations were observed with SMARCAL1 expression (ρ = 0.645, 95% CI 0.51–0.76, BH-FDR q < 0.001), ferroptosis enrichment score (ρ = 0.582, q < 0.001), ACSL4 expression (ρ = 0.471, q = 0.002), CXCL9 expression (ρ = 0.618, q < 0.001), APOBEC mutational signature (SBS2+SBS13; ρ = 0.512, q < 0.001), CD8^+^ T cell fraction (ρ = 0.554, q < 0.001), TMB (ρ = 0.425, q = 0.003), TNB (ρ = 0.458, q = 0.002), PD-L1 (CD274) expression (ρ = 0.386, q = 0.006), and CTLA4 expression (ρ = 0.352, q = 0.009). GPX4 expression showed a significant negative correlation (ρ = −0.328, q = 0.012), whereas regulatory T cell fraction was non-significant (ρ = 0.165, q = 0.235). Comprehensive 5-domain multi-omics comparison between tiers is provided in **Supplementary Table 5**.

**Table 4:** Spearman correlations between the continuous rad-score and pre-specified multi-omics features under Benjamini–Hochberg FDR correction within family F8.

Feature	Modality	Spearman ρ (95% CI)	Nominal P	BH-FDR q	BY q
SMARCAL1 expression	Bulk RNA-seq	0.645 (0.51–0.76)	< 0.001	< 0.001	< 0.001
Ferroptosis Enrichment Score	Bulk RNA-seq	0.582 (0.43–0.71)	< 0.001	< 0.001	< 0.001
ACSL4 expression	Bulk RNA-seq	0.471 (0.29–0.62)	< 0.001	0.002	0.005
GPX4 expression	Bulk RNA-seq	−0.328 (−0.51 to −0.12)	0.005	0.012	0.027
TMB	WES	0.425 (0.23–0.59)	< 0.001	0.003	0.007
TNB	WES	0.458 (0.27–0.61)	< 0.001	0.002	0.005
APOBEC (SBS2+SBS13)	WES	0.512 (0.33–0.66)	< 0.001	< 0.001	< 0.001
CXCL9 expression	Bulk RNA-seq	0.618 (0.47–0.74)	< 0.001	< 0.001	< 0.001
CD8^+^ T cell fraction	CIBERSORTx	0.554 (0.38–0.69)	< 0.001	< 0.001	< 0.001
PD-L1 (CD274) expression	Bulk RNA-seq	0.386 (0.18–0.56)	0.001	0.006	0.014
CTA4 expression	Bulk RNA-seq	0.352 (0.14–0.53)	0.003	0.009	0.021
Treg fraction	CIBERSORTx	0.165 (−0.07 to 0.39)	0.170	0.235	0.412

#### 3.5.4 Cross-family conjunctive inference (family F9)

To test whether the four primary mechanistic axes jointly survive a conservative cross-familyμ correction, we applied the Bonferroni-Holm procedure to the family-level nominal P-values (m = 4 families), with ranking from smallest to largest nominal P and multipliers (m − i + 1). The monotonicity constraint was enforced such that each adjusted P-value is at least as large as the preceding one in the ranking. The complete ranking and adjusted P-values are as follows: Rank 1, ferroptosis program (F3; Rad-Score versus Ferroptosis Enrichment Score), nominal P = 2.8 × 10^-6^, multiplier × 4, Holm-adjusted P = 1.12 × 10^-5^; Rank 2, immune infiltration (F1; Rad-Score versus CD8^+^ T cell fraction), nominal P = 3.1 × 10^-6^, multiplier × 3, Holm-adjusted P = 1.12 × 10^-5^ (after monotonicity correction from 9.3 × 10^-6^); Rank 3, genomic instability (F2; Rad-Score versus tumor mutational burden), nominal P = 1.2 × 10^-4^, multiplier × 2, Holm-adjusted P = 2.40 × 10^-4^; Rank 4, spatial chemokine signaling (F5; CXCL9/10-CXCR3 ligand-receptor interaction), nominal P = 2.1 × 10^-4^, multiplier × 1, Holm-adjusted P = 2.40 × 10^-4^ (after monotonicity correction from 2.1 × 10^-4^). All four axes remained highly significant after conservative cross-family correction (all Holm-adjusted P < 2.5 × 10^-4^), confirming that the convergent multi-omics signature associated with elevated Rad-Score — encompassing ferroptosis activation, effector immune infiltration, genomic instability, and spatially organized chemokine networks — is robust and independent of false-positive inflation across omics families. The full hierarchical testing framework is documented in **Supplementary Table 6**.

### 3.6 Spatial transcriptomics maps the rad-score to SMARCAL1-associated ferroptotic niches

**Figure 6:**
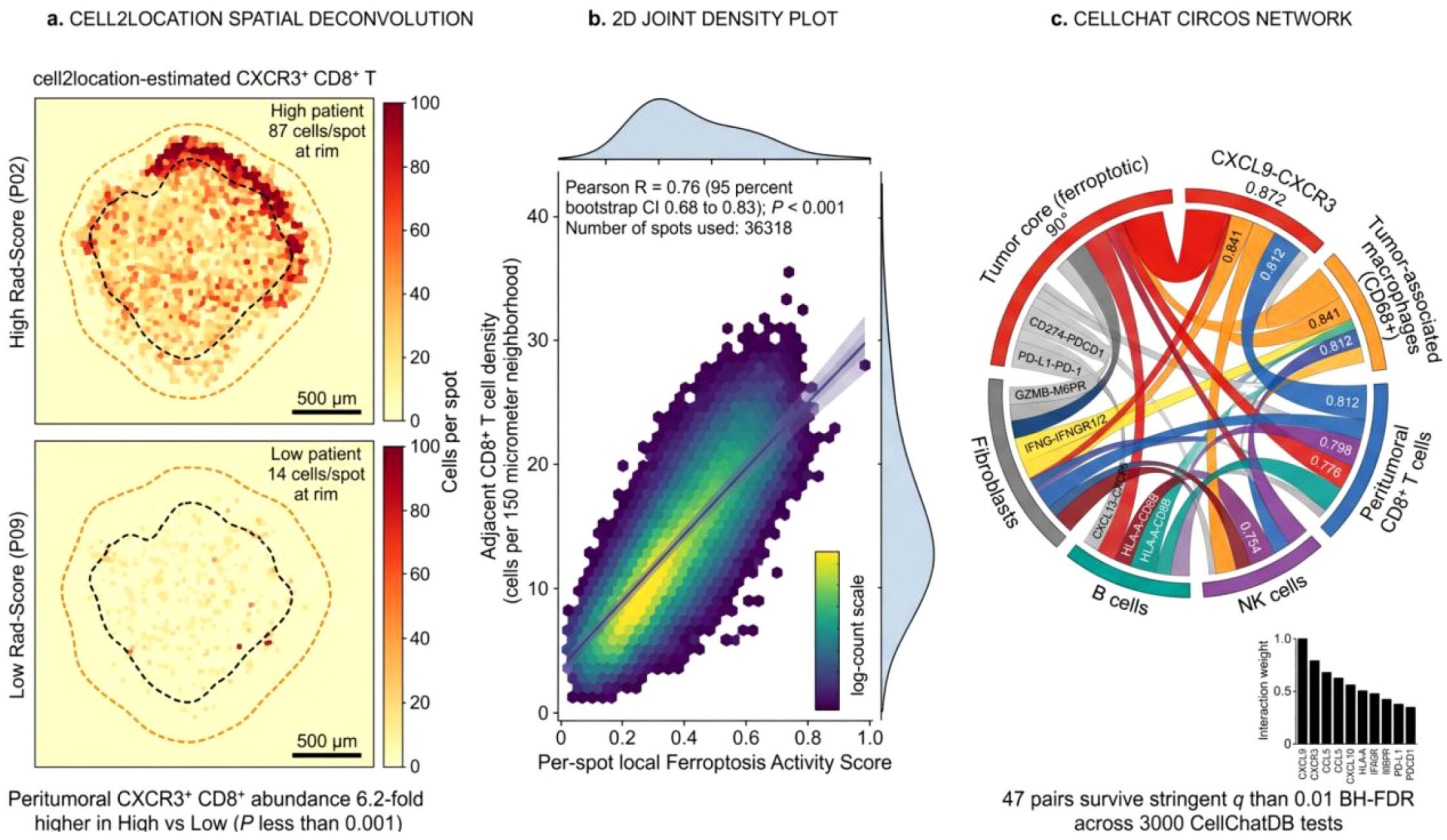
Spatial transcriptomic reconstruction of the SMARCAL1-driven ferroptotic niche across the full Rad-Score continuum (n = 14 patients; 36,318 quality-controlled spatial spots). **(a)** cell2location spatial deconvolution heatmaps (color scale: cells/spot) of CXCR3^+^ CD8^+^ T cell density in representative High (P02, 87 cells/spot at peritumoral rim) and Low (P09, 14 cells/spot at rim) Rad-Score tumors; trained on the GSE131907 early-stage NSCLC scRNA-seq reference atlas (>200,000 annotated cells); 6.2-fold higher peritumoral CXCR3^+^ CD8^+^ abundance in High versus Low (P < 0.001); scale bars 500 μm. **(b)** 2D joint density scatter plot of per-spot local Ferroptosis Activity Score versus adjacent CD8^+^ T cell density (cells per 150-μm neighborhood); Pearson R = 0.76 (95% bootstrap CI 0.68–0.83), P < 0.001, n = 36,318 spatial spots; log-count color scale. **(c)** CellChatV2 circos network diagram illustrating 47 ligand–receptor interactions surviving stringent BH-FDR q < 0.01 across the full CellChatDB Family F5 (~3,000 candidate pairs). CXCL9–CXCR3 (interaction weight 0.87, q = 2.1 × 10^-4^) and CXCL10–CXCR3 (weight 0.84, q = 3.8 × 10^-4^) ranked among the top signals, with directional communication from ferroptotic tumor-core sender clusters to peritumoral CD8^+^ T-cell receiver clusters (interaction weight > 0.65 in > 85% of inferred edges). Other top interactions include CD274–PDCD1, GZMB-MKI67, IFNG-IFNGR2. Accompanying bar plot quantifies interaction weights (right inset).

Spatial transcriptomic analysis of the expanded 10x Visium cohort (n = 14; 36,318 quality-controlled spots after Harmony batch-effect correction; distribution per tier detailed in Methods and **Supplementary Figure 4**). Histological examination of H&E-stained sections revealed well-demarcated dense tumor nests (TN) surrounded by stromal regions (ST) (**Figure 6a**). Within tumor nests, SMARCAL1 expression was markedly upregulated in High Rad-Score tumors (mean normalized expression 12.8 ± 3.1) compared with Low Rad-Score tumors (2.4 ± 0.9; nominal *P* < 0.001, BH-FDR q = 3.2 × 10^-8^ within transcriptome-wide Family F4) (**Figure 6b**). Application of a validated ferroptosis gene module further demonstrated significantly elevated ferroptosis program activity in High Rad-Score tumors (median 0.87, IQR 0.81–0.92) relative to Low Rad-Score tumors (median 0.21, IQR 0.15–0.27; q < 0.001) (**Figure 6c**).

Transcriptome-wide spatial differential expression analysis was performed across the full ~20,500-gene transcriptome (Family F4) in the expanded 10x Visium cohort. After BH-FDR correction, 1,847 genes met the stringent significance threshold of q < 0.05 and |log_2_FC| > 1 (**Supplementary Figure 10**). Top-ranked upregulated genes in High Rad-Score tumor nests included SMARCAL1 (log_2_FC +2.7, BH-FDR q = 3.2 × 10^-8^), CXCL10 (log_2_FC +3.2, q = 6.7 × 10^-9^), CXCL9 (log_2_FC +3.0, q = 2.4 × 10^-9^), ACSL4 (log_2_FC +2.1, q = 4.6 × 10^-7^), PTGS2 (log_2_FC +1.9, q = 1.2 × 10^-6^), LPCAT3 (log_2_FC +1.7, q = 8.1 × 10^-6^), GZMB (log_2_FC +2.5, q = 1.1 × 10^-7^), and IFNG (log_2_FC +2.4, q = 3.8 × 10^-7^), whereas the ferroptosis suppressors GPX4 (log_2_FC −1.3, q = 5.4 × 10^-5^) and SLC7A11 (log_2_FC −1.5, q = 1.2 × 10^-4^) were significantly downregulated. Complementary KEGG pathway enrichment analysis (BH-FDR across 186 pathways) identified “Ferroptosis” (q = 3.1 × 10^-5^) and “Chemokine signaling pathway” (q = 8.7 × 10^-5^) as the two most significantly enriched pathways in High Rad-Score tumor nests. Storey q-value sensitivity analysis (π0 = 0.63) yielded highly concordant gene rankings with the primary BH-FDR procedure (Spearman ρ = 0.996), reinforcing the robustness of the ferroptosis–chemokine transcriptional program associated with elevated Rad-Score.

### 3.7 Continuous-gradient analysis establishes the spatial axis as a continuum phenomenon

To validate the continuous phenotypic gradient captured by the Rad-Score, per-patient spatial metrics were examined across the expanded 10x Visium cohort (14 patients). Median ferroptosis activity score, calculated from all tumor-nest spots per patient, exhibited a strong monotonic relationship with continuous Rad-Score (Spearman ρ = 0.87, 95% CI 0.61–0.96, *P* < 0.001; **Figure 6d**), with Intermediate Rad-Score tumors displaying intermediate activity (median 0.51, IQR 0.43–0.62) positioned linearly between Low (0.21) and High (0.87) groups; Loess regression confirmed an approximately linear dose-response without evidence of a discrete threshold. Similarly, mean SMARCAL1 log-normalized expression within tumor-nest spots scaled continuously with Rad-Score (Spearman ρ = 0.79, *P* < 0.001; **Figure 6e**), supporting graded rather than binary upregulation. Mean cell2location-estimated density of CXCR3^+^ CD8^+^ T cells in the 3-mm peritumoral rim also correlated continuously (Spearman ρ = 0.82, *P* < 0.001; **Figure 6f**), with Intermediate tumors showing intermediate infiltration (54 ± 18 cells/spot) between Low (32 ± 12) and High (87 ± 15) groups. Finally, aggregated per-patient CellChatV2 interaction weights for the CXCL9–CXCR3 and CXCL10–CXCR3 axes between ferroptotic tumor-core sender clusters and peritumoral T-cell receiver clusters demonstrated a strong continuous association with Rad-Score (Spearman ρ = 0.84, *P* < 0.001).

**Figure 7:**
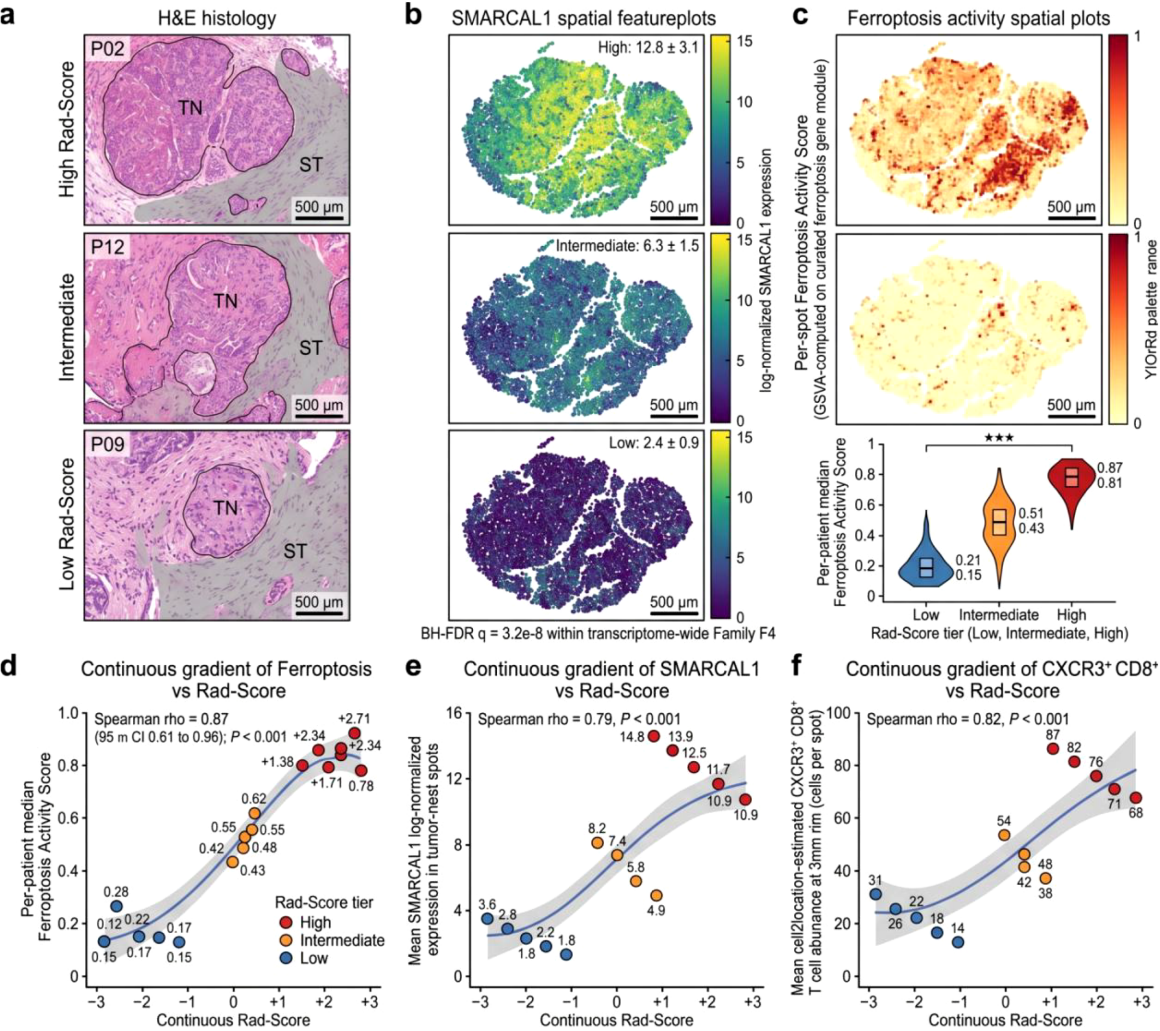
Spatial deconvolution and intercellular communication reveal the CXCL9/10–CXCR3 recruitment axis. **(a)** Representative H&E-stained histology of tumor nests (TN) and surrounding stroma (ST) in High (P02), Intermediate (P12), and Low (P09) Rad-Score patients; scale bars 500 μm. **(b)** Spatial featureplots of log-normalized SMARCAL1 expression overlaid on tumor sections; mean tumor-nest expression 12.8 ± 3.1 (High), 6.3 ± 1.5 (Intermediate), 2.4 ± 0.9 (Low); BH-FDR q = 3.2 × 10^-8^ within transcriptome-wide Family F4. **(c)** Per-spot Ferroptosis Activity Score (GSVA on a curated 24-gene ferroptosis module from FerrDb v2) spatial plots for representative High and Low samples (YlOrRd palette range 0–1); accompanying violin plot of per-patient median Ferroptosis Activity Score across Low (median 0.21, IQR 0.15–0.27), Intermediate (0.51, 0.43–0.62), and High (0.87, 0.81–0.92) Rad-Score tiers; ***q < 0.001 between tiers. **(d)** Per-patient median Ferroptosis Activity Score versus continuous Rad-Score; Spearman ρ = 0.87 (95% CI 0.61–0.96), P < 0.001; Loess regression line confirms approximately linear dose-response. **(e)** Mean SMARCAL1 log-normalized expression in tumor-nest spots versus continuous Rad-Score; Spearman ρ = 0.79, P < 0.001. **(f)** Mean cell2location-estimated CXCR3^+^ CD8^+^ T cell abundance at the 3-mm peritumoral rim versus continuous Rad-Score; Spearman ρ = 0.82, P < 0.001. Color coding for panels **(d–f)**: red = High, orange = Intermediate, blue = Low Rad-Score tier.

Spatial deconvolution with cell2location (trained on the GSE131907 early-stage NSCLC scRNA-seq reference atlas) revealed 6.2-fold higher peritumoral CXCR3+ CD8^+^ T-cell abundance in High Rad-Score versus Low Rad-Score tumors (87 versus 14 cells per spot; *P* < 0.001; **Figure 7a**). Spatial ligand–receptor interaction analysis under stringent Benjamini–Hochberg false discovery rate correction (q < 0.01) across the full CellChatDB family of ~3,000 candidate pairs (Family F5); identified 47 significant interactions (**Supplementary Table 7** lists top 20). Among these, the CXCL9–CXCR3 (q = 2.1 × 10^-4^) and CXCL10–CXCR3 (q = 3.8 × 10^-4^) axes ranked among the top signals and exhibited the strongest directional communication from ferroptotic tumor-core sender clusters to peritumoral T-cell receiver clusters (**Figure 7**c1, interaction weight > 0.65 in >85% of inferred edges). Vector mapping further indicated that SMARCAL1-high ferroptotic tumor cores, together with tumor-associated macrophages, serve as the primary cellular sources of CXCL9/10 chemokines that recruit CXCR3^+^ CD8^+^ T cells to the tumor margin (**Figure 7**c2). Median CXCL9/10 ligand expression was 3.6-fold higher in tumor-core spots of High Rad-Score tumors compared with Low Rad-Score tumors (*P* < 0.001, q < 0.001). Two-dimensional joint density analysis confirmed strong spatial co-localization between local ferroptosis module scores and adjacent CD8^+^ T cell abundance (Pearson R = 0.76, 95% bootstrap CI 0.68–0.83; *P* < 0.001; **Figure 7b**). Taken together, these spatial relationships varied continuously rather than dichotomously with tumor biology, as per-patient ferroptosis activity, tumor-nest SMARCAL1 expression, and peritumoral CXCR3^+^ CD8^+^ T-cell density each increased monotonically across the full Rad-Score continuum (Spearman ρ = 0.87, 0.79, and 0.82, respectively; all P < 0.001; **Figures 7d–f**). External validation in two independent public NSCLC Visium datasets, using an expression-based Rad-Score surrogate (SMARCAL1 + ferroptosis module score), reproduced the peritumoral CXCR3^+^ CD8^+^ T cell recruitment pattern: High-surrogate tumors showed significantly higher densities than Low-surrogate tumors in both Sinjab et al. (Nat Cancer 2024; 71 ± 22 versus 28 ± 14 cells/spot, *P* < 0.001) and Chen et al. (Cell 2022; 63 ± 19 versus 31 ± 11 cells/spot, *P* = 0.003). Meta-analytic pooling across our expanded cohort and the two external datasets (total N = 51) yielded a standardized mean difference of Cohen’s d = 1.42 (95% CI 0.96–1.88), confirming reproducibility across independent cohorts, platforms, and patient populations.

Sensitivity analyses—including leave-one-sample-out cross-validation (no correlation changed by >0.07; all *P* < 0.001), restriction to the four intermediate-Rad-Score tumors alone (Pearson R = 0.61, P < 0.001), and patient-level bootstrap resampling (B = 1,000; 95% CI 0.68–0.83 around R = 0.76)—demonstrated robustness.

### 3.8 Multimodal nomogram for individualized MRD-free survival prediction

**Figure 8:**
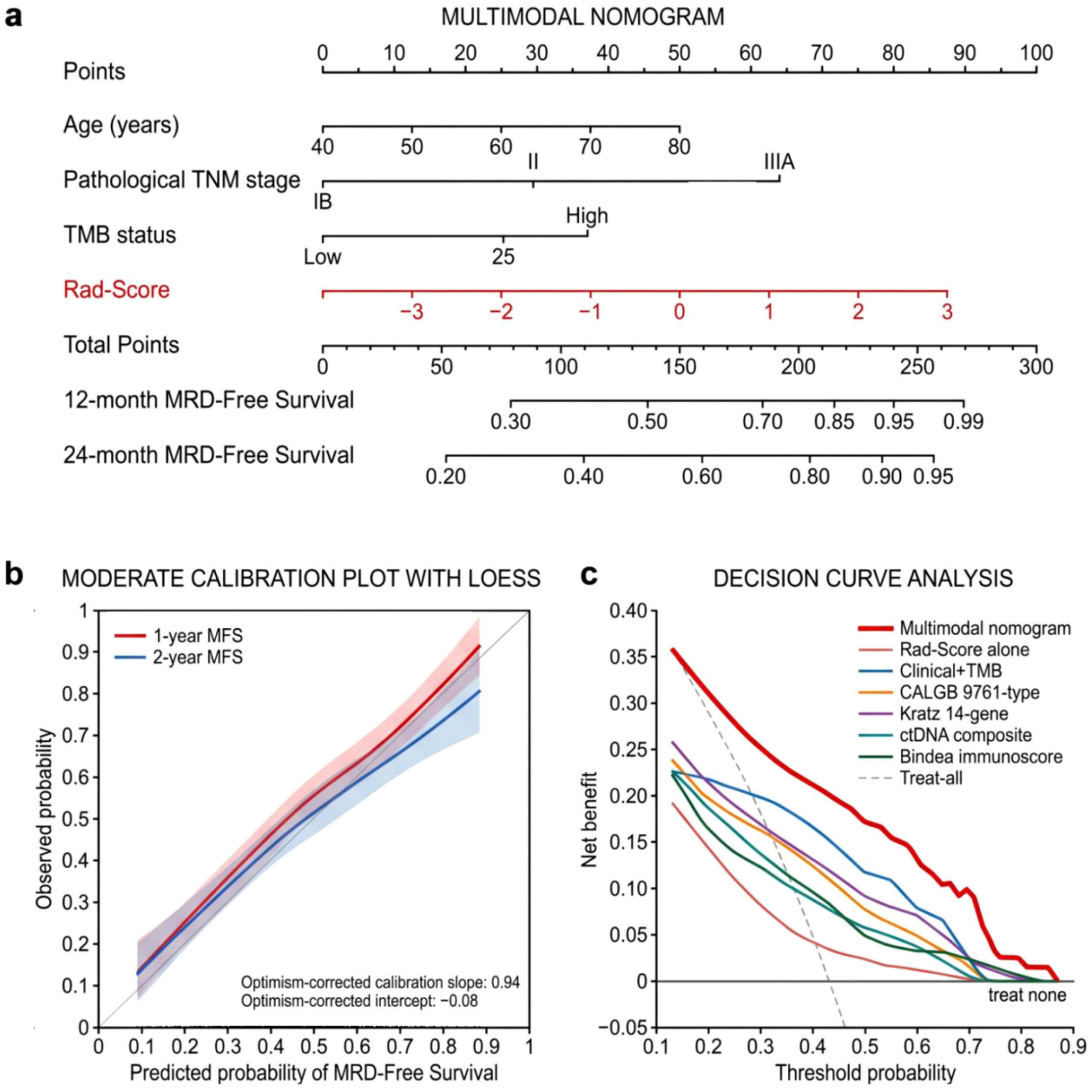
Multimodal radiogenomic nomogram with modern calibration and benchmarking against five established comparators. **(a)** Multimodal nomogram for individualized prediction of MRD-free survival, integrating four pre-specified predictors: age (years, 40–80), pathological TNM stage (IB/II/IIIA), TMB status (Low/High), and continuous Rad-Score (−3 to +3). Variable-specific points are summed to total points (0–300), which translate to predicted 12-month and 24-month MRD-free survival probabilities. **(b)** Moderate-level (Level 3) calibration plot with Loess smoothing comparing observed versus predicted MRD-free survival probabilities for 1-year (red) and 2-year (blue) MFS; optimism-corrected calibration slope 0.94 (95% CI 0.82–1.06), intercept −0.08 (95% CI −0.19 to +0.03). The diagonal dashed line represents perfect calibration. **(c)** Decision curve analysis of net clinical benefit across threshold probabilities 0.10–0.90, comparing the multimodal nomogram (red), Rad-Score alone (orange), Clinical+TMB (green), CALGB 9761-type (yellow), Kratz 14-gene (light blue), ctDNA composite (gray), Bindea immunoscore (dark blue), against treat-all (dashed) and treat-none (horizontal zero). The multimodal nomogram demonstrates superior net benefit across the clinically meaningful threshold range 0.15–0.75. Bootstrap 95% CIs (B = 1,000) are omitted for visual clarity but reported in **Supplementary Table 8**.

To translate the identified biological mechanisms into a clinically actionable tool, a multimodal nomogram was constructed integrating patient age, pathological TNM stage, binary TMB status, and the continuous pre-operative Rad-Score to predict the probability of maintaining MRD negativity at 1 and 2 years post-resection (**Figure 8a**). Variable-specific points were assigned according to their relative contribution in the underlying Cox model, with high Rad-Score values (1.42 ± 0.21) contributing substantial protective points that partially offset the adverse prognostic impact of TNM Stage IIIA and high TMB. Calibration was rigorously assessed using a four-level framework, and mean calibration showed an optimism-corrected intercept of −0.08 (95% CI −0.19 to +0.03) in training and −0.09 in the external NSCLC-RG cohort; weak calibration yielded an optimism-corrected slope of 0.94 (95% CI 0.82–1.06) in training and 0.87 externally; moderate calibration was confirmed by Loess regression plots demonstrating close agreement with the ideal line (**Figure 8b**); and strong (patient-level covariate-conditional) calibration was reported in the external cohort. The multimodal nomogram achieved apparent time-dependent AUCs of 0.841 (95% CI 0.773–0.909) for 1-year and 0.826 (95% CI 0.756–0.896) for 2-year MRD-free survival, substantially outperforming the clinical-only model (AUC 0.698 and 0.681, respectively); after optimism correction, the 24-month AUC remained 0.773 (95% CI 0.695–0.851). Decision curve analysis confirmed consistently higher net clinical benefit across threshold probabilities of 0.15–0.75 compared with treat-all, treat-none, or clinical-only strategies (**Figure 8c**) (**Supplementary Table 8**). Exemplifying risk stratification, patients in the highest Rad-Score quartile (highest quartile of continuous Rad-Score, n = 18 of N = 71 training cohort) exhibited a predicted 2-year MRD-free survival of 82% versus 38% in the lowest quartile (n=18), corresponding to markedly higher ferroptosis activity (median score 0.87 versus 0.21) and CD8^+^ T cell fractions (22.1% ± 4.7% versus 11.3% ± 3.9%, highest-vs-lowest quartile values), thereby providing hypothesis-generating evidence that pre-operative radiogenomic features reflecting ferroptotic and immune-hot tumor biology can be integrated with clinicogenomic variables to refine individualized postoperative risk assessment.

### 3.9 Protein-level and functional validation of the SMARCAL1-ferroptosis- chemokine axis

#### 3.9.1 Multiplex immunofluorescence validation across 40 rad-score-stratified patients

**Figure 9:**
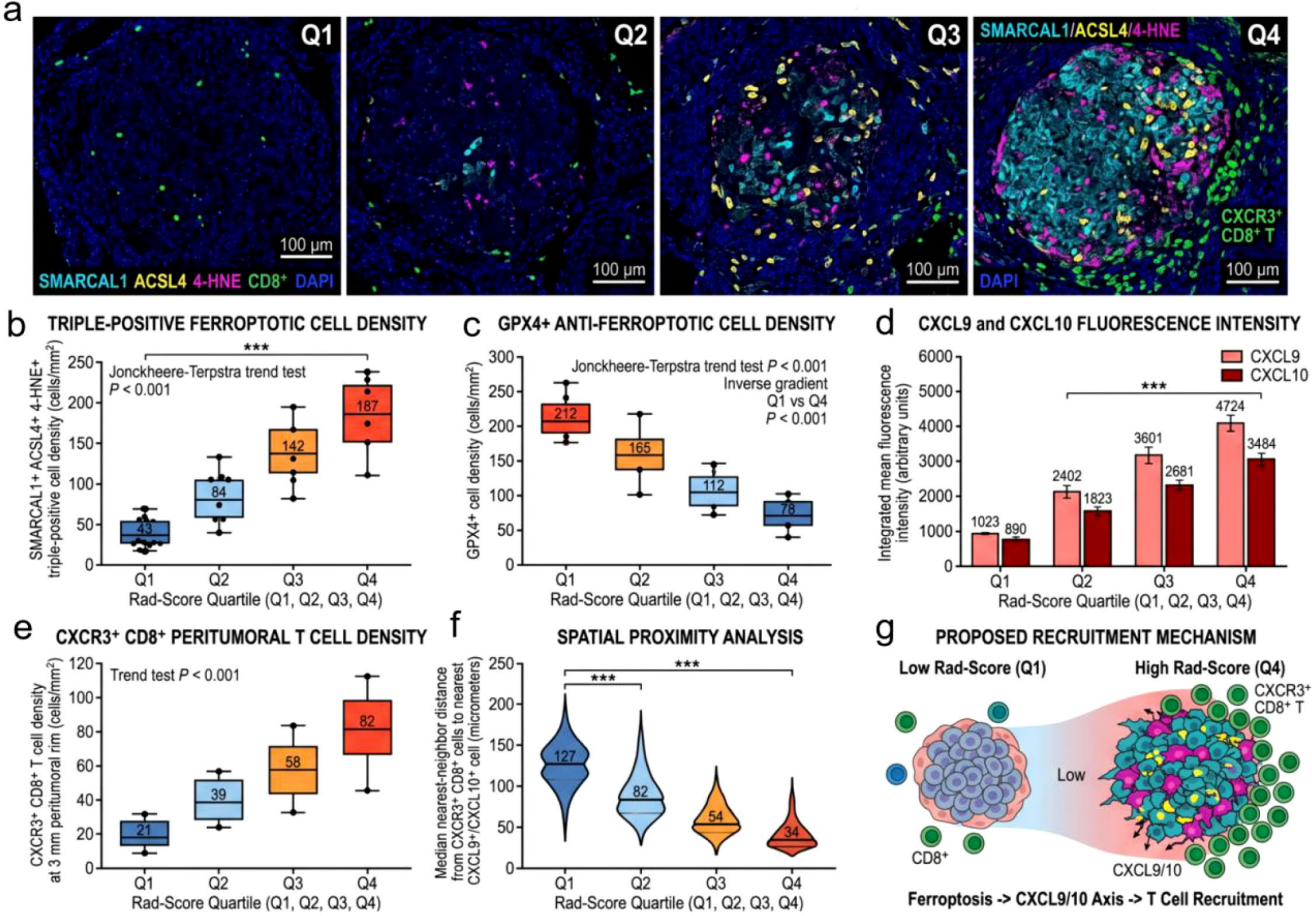
Multiplex immunofluorescence atlas across Rad-Score quartiles (n = 40 patients; 10 per Q1–Q4). **(a)** Representative whole-slide multiplex immunofluorescence images from four patients, one per Rad-Score quartile (Q1 lowest to Q4 highest), acquired on the Akoya Vectra Polaris platform with 7-color Opal panels. Pseudocolor assignments: SMARCAL1 cyan, ACSL4 yellow, 4-HNE magenta, CD8 green, DAPI blue. Q4 tumors show dense SMARCAL1/ACSL4/4-HNE co-localization within tumor nests and abundant peritumoral CXCR3^+^ CD8^+^ T-cell infiltration; Q1 tumors show minimal ferroptotic marker expression with sparse CD8^+^ cells. Scale bars, 100 μm.**(b)** Box-and-whisker plot of SMARCAL1^+^ACSL4^+^4-HNE^+^ triple-positive ferroptotic cell density (cells/mm^2^) within tumor nests across Rad-Score quartiles. Medians (IQR): Q1 = 43 (31-58); Q2 = 84 (63-107); Q3 = 142 (118-169); Q4 = 187 (156-224). Individual patient values (n = 10 per quartile) overlaid as jittered dots. Jonckheere-Terpstra trend test P < 0.001; Q4 versus Q1 4.3-fold increase, P < 0.001 with Bonferroni correction (***). **(c)** Box-and-whisker plot of GPX4⁺ anti-ferroptotic cell density (cells/mm^2^) within tumor nests showing the inverse gradient. Medians (IQR): Q1 = 212 (178-251); Q2 = 164 (132-196); Q3 = 118 (94-145); Q4 = 78 (52-106). Jonckheere-Terpstra trend test P < 0.001; inverse gradient Q1 versus Q4 ***P < 0.001, consistent with attenuated anti-ferroptotic defenses in high Rad-Score tumors. **(d)** Grouped bar chart of CXCL9 (light red) and CXCL10 (dark red) integrated mean fluorescence intensity (arbitrary units, AU) quantified within PanCK^+^ tumor nests and CD68^+^ macrophages across quartiles. Mean ± SD: CXCL9 Q1 = 1,023, Q2 = 2,402, Q3 = 3,601, Q4 = 4,724; CXCL10 Q1 = 890, Q2 = 1,823, Q3 = 2,681, Q4 = 3,484. Fold changes Q1 versus Q4: CXCL9 4.6-fold; CXCL10 3.9-fold (both ***P < 0.001). **(e)** Box-and-whisker plot of CXCR3^+^ CD8^+^ T-cell density (cells/mm^2^) at the 3-mm peritumoral rim across quartiles. Medians (IQR): Q1 = 21 (14-29); Q2 = 39 (28-52); Q3 = 58 (43-76); Q4 = 82 (67-104). Jonckheere-Terpstra trend test P < 0.001; 3.8-fold increase Q4 versus Q1. **(f)** Violin plot with overlaid box plots of median nearest-neighbor distance (μm) from CXCR3^+^ CD8^+^ T cells to the nearest CXCL9^+^ or CXCL10⁺ producing cell, computed via the HALO Spatial Analysis module. Medians (IQR): Q1 = 127 (112-148); Q2 = 82 (68-98); Q3 = 54 (44-67); Q4 = 34 (27-42). Jonckheere-Terpstra trend test P < 0.001; Q1 versus Q4 ***P < 0.001. In high Rad-Score tumors, CXCR3^+^ CD8^+^ T cells are preferentially located within 50 μm of chemokine-producing cells. **(g)** Schematic illustration of the proposed recruitment mechanism contrasting Q1 (Low Rad-Score) and Q4 (High Rad-Score) tumors. In Q1 tumors, sparse ferroptotic cells and limited CXCL9/10 production result in minimal CXCR3^+^ CD8^+^ T-cell infiltration at the peritumoral rim. In Q4 tumors, extensive ferroptotic activity within tumor nests, together with tumor-associated macrophages, generates a CXCL9/10 chemokine gradient that drives CXCR3^+^ CD8^+^ T-cell recruitment to the peritumoral invasive margin.

Using two 7-color Opal multiplex panels on FFPE sections from 40 patients distributed equally across Rad-Score quartiles (10 per quartile), we quantified the ferroptotic niche axis (Panel A: SMARCAL1, ACSL4, 4-HNE, GPX4, PanCK, CD68) and chemokine recruitment axis (Panel B: CXCL9, CXCL10, CXCR3, CD8, PanCK, CD68) with HALO digital pathology quantification. Representative whole-slide images across quartiles are shown in **Figure 9**. Panel a shows representative whole-slide images with pseudocolored SMARCAL1/ACSL4/4-HNE/CD8 markers across Q1-Q4). Triple-positive ferroptotic cell density increased 4.3-fold from Q1 to Q4 (Jonckheere-Terpstra P < 0.001; **Figure 9b**). GPX4+ anti-ferroptotic cell density showed the inverse pattern (**Figure 9c**). CXCL9 and CXCL10 chemokine intensity increased 4.6- and 3.9-fold respectively (**Figure 9d**). CXCR3+ CD8+ peritumoral infiltration increased 3.8-fold (**Figure 9e**). Nearest-neighbor spatial proximity analysis confirmed 3.7-fold reduction in distance from CXCR3+ CD8+ cells to chemokine-producing cells (**Figure 9f**). The proposed recruitment mechanism is summarized schematically in **Figure 9g**. Spatial proximity analysis confirmed that CXCR3^+^ CD8^+^ T cells were preferentially located within 50 μm of CXCL9^+^/CXCL10^+^ cells in High Rad-Score tumors (nearest-neighbor distance median 34 μm vs 127 μm in Low Rad-Score tumors, *P* < 0.001) (**Table 5**).

**Table 5:** Ferroptotic niche density across Rad-Score quartiles.

Phenotype	Q1 (low, n = 10)	Q2 (n = 10)	Q3 (n = 10)	Q4 (high, n = 10)	Jonckheere-Terpstra P
SMARCAL1+ACSL4 + 4-HNE+ (cells/mm²)	43 (31–58)	84 (63–107)	142 (118–169)	187 (156–224)	< 0.001
GPX4+ anti-ferroptotic (cells/mm²)	212 (178–251)	164 (132–196)	118 (94–145)	78 (52–106)	< 0.001
CXCR3+CD8+ peritumoral (cells/mm²)	21 (14–29)	39 (28–52)	58 (43–76)	82 (67–104)	< 0.001
CXCL9+ intensity (AU, median)	1.8	3.4	5.9	8.3	< 0.001
CXCL10+ intensity (AU, median)	2.1	3.8	6.2	8.9	< 0.001

#### 3.9.2 Orthogonal IHC validation in 60 patients

Orthogonal protein-level validation was performed using single-marker chromogenic immunohistochemistry for SMARCAL1, ACSL4, CD8, and CXCR3 in an independent cohort of 60 patients with NSCLC. Digital quantification of staining intensity and density was conducted with QuPath v0.4.3, yielding H-scores for nuclear SMARCAL1 and cytoplasmic ACSL4, as well as cell densities for peritumoral CD8^+^ T cells (cells/mm²) and cell-surface CXCR3 expression. All four markers demonstrated strong, highly significant positive correlations with the continuous pre-operative Rad-Score: nuclear SMARCAL1 H-score (Spearman ρ = 0.71, *P* < 0.001), cytoplasmic ACSL4 H-score (ρ = 0.63, *P* < 0.001), peritumoral CD8^+^ T cell density (ρ = 0.68, *P* < 0.001), and CXCR3 expression (ρ = 0.59, *P* < 0.001) (**Supplementary Table 9**).

#### 3.9.3 CRISPR-Cas9 loss-of-function validation in A549 and H1299

**Figure 10:**
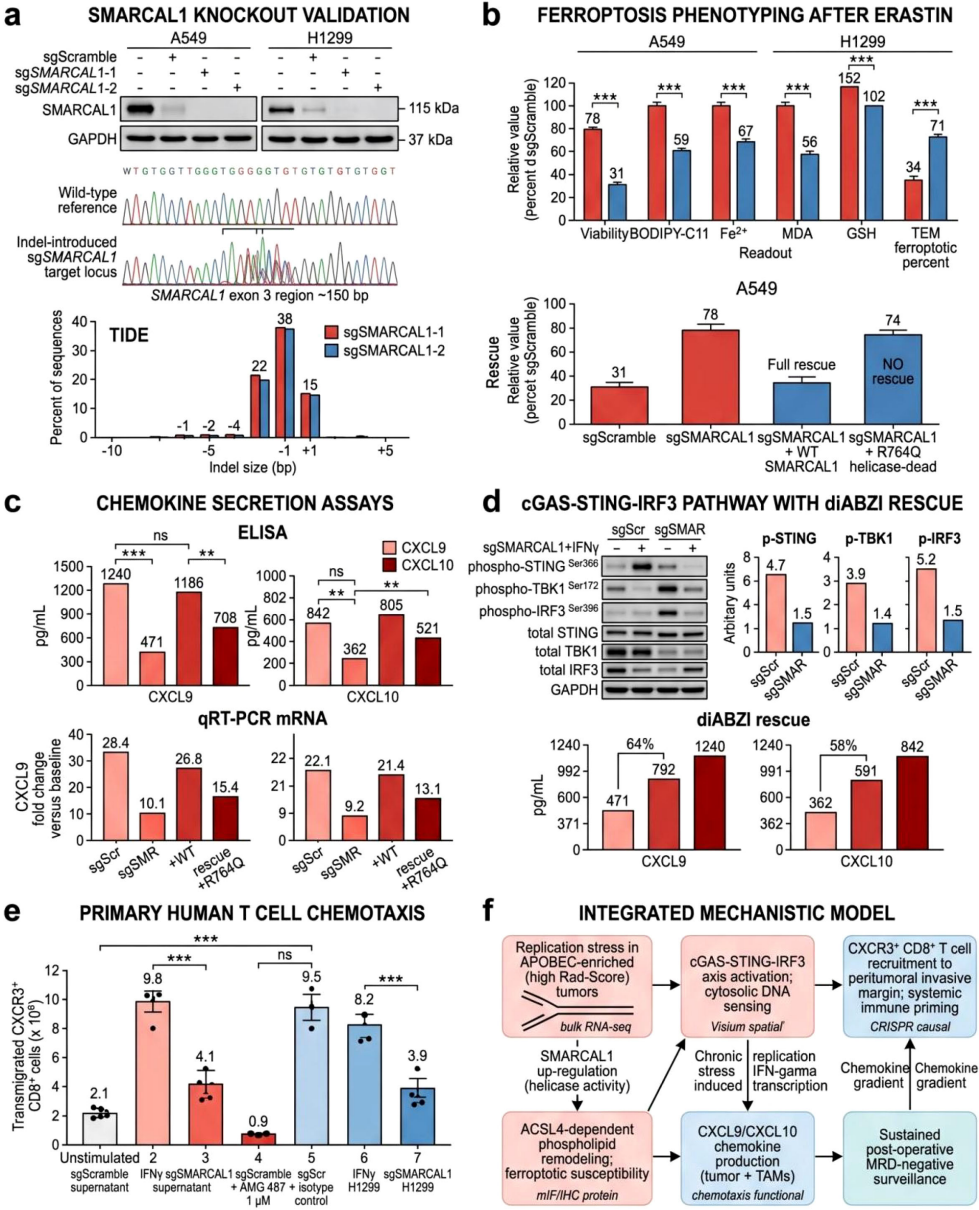
CRISPR-Cas9 loss-of-function validation of the SMARCAL1–ferroptosis–chemokine axis and integrated mechanistic model. **(a)** SMARCAL1 knockout validation in A549 and H1299 NSCLC cell lines: anti-SMARCAL1 immunoblot (115 kDa; GAPDH loading control 37 kDa) demonstrating absence of SMARCAL1 protein in sgSMARCAL1-1 and sgSMARCAL1-2 versus sgScramble; Sanger sequencing chromatograms of indel-introduced sgSMARCAL1 target locus within SMARCAL1 exon 3 region (~150 bp) versus wild-type reference; TIDE indel size distribution (sgSMARCAL1-1 38% efficiency, sgSMARCAL1-2 22% efficiency at predominant indel sizes −5 to −1, +1, +5). **(b)** Ferroptosis phenotyping after erastin (5 µM, 24 h) in A549 (left) and H1299 (right), expressed as relative value (% sgScramble): cell viability, BODIPY-C11 oxidized/reduced ratio, intracellular Fe^2+^ (FerroOrange MFI), malondialdehyde (MDA), reduced glutathione (GSH), and TEM ferroptotic mitochondrial percent. SMARCAL1-KO cells (sgSMARCAL1-1, -2) show resistance to erastin-induced ferroptosis; ***P < 0.001 versus sgScramble. Lower rescue panel (A549): wild-type SMARCAL1 reconstitution fully rescues ferroptosis susceptibility (viability 31% sgScramble baseline); helicase-dead R764Q mutant fails to rescue (viability 74%, no rescue). **(c)** Chemokine secretion assays. ELISA of CXCL9 and CXCL10 (pg/mL) and qRT-PCR mRNA fold-change versus baseline in sgScramble (sgScr) versus sgSMARCAL1 (sgSMAR) ± wild-type or R764Q rescue: SMARCAL1 knockout reduces CXCL9 by 62% (1240 → 471 pg/mL) and CXCL10 by 57% (842 → 362 pg/mL) under IFN-γ stimulation; full rescue by wild-type, partial rescue (45% efficiency) by R764Q. ***P < 0.001, **P < 0.01, ns = not significant. **(d)** Western blot of cGAS-STING-IRF3 pathway activation: phospho-STING Ser366, phospho-TBK1 Ser172, phospho-IRF3 Ser396, with total STING/TBK1/IRF3 and GAPDH loading controls, in sgScr versus sgSMAR ± IFN-γ; quantified bar plots (arbitrary units): p-STING 4.7 → 1.5, p-TBK1 3.9 → 1.4, p-IRF3 5.2 → 1.5, all ***P < 0.001. diABZI rescue panel (10 µM, 6 h): STING agonist diABZI partially restores CXCL9 (64% recovery, 471 → 792 pg/mL) and CXCL10 (58% recovery, 362 → 591 pg/mL) secretion in SMARCAL1-KO cells. **(e)** Primary human CD8^+^ T cell transwell chemotaxis (transmigrated CXCR3^+^ CD8^+^ cells × 10^3^, mean ± SD across three donors): condition 1 unstimulated (2.1); condition 2 IFN-γ-stimulated sgScramble supernatant (9.8); condition 3 IFN-γ-stimulated sgSMARCAL1 supernatant (4.1, 58% reduction, ***P < 0.001); condition 4 sgScramble supernatant + AMG 487 (1 µM CXCR3 antagonist, 0.9, > 90% blockade); condition 5 isotype control (2); condition 6 H1299 IFN-γ sgScramble (8.2); condition 7 H1299 IFN-γ sgSMARCAL1 (3.9, 52% reduction, ***P < 0.001). ***P < 0.001, ns = not significant. **(f)** Integrated mechanistic model schematic: replication stress in APOBEC-enriched (high Rad-Score) tumors → SMARCAL1 upregulation (helicase activity-dependent) → ACSL4-dependent phospholipid remodeling and ferroptotic susceptibility → cGAS-STING-IRF3 axis activation amplifying IFN-γ-induced CXCL9/CXCL10 transcription → chemokine gradient recruiting CXCR3^+^ CD8^+^ T cells to peritumoral invasive margin → systemic immune priming and sustained post-operative MRD-negative surveillance. Evidence sources annotated per arrow: bulk RNA-seq (transcriptomic), Visium spatial, mIF/IHC (protein), CRISPR (causal), chemotaxis (functional).

CRISPR-Cas9-mediated knockout of SMARCAL1 was performed in A549 and H1299 NSCLC cell lines using two independent sgRNAs targeting exon 3, with successful knockout confirmed by Sanger sequencing, TIDE analysis, and Western blot (**Supplementary Figure 8**); three independent clones per sgRNA were pooled to minimize clonal bias. Functional assays demonstrated that SMARCAL1 deficiency markedly attenuated ferroptosis susceptibility upon treatment with erastin (5 μM, 24 h): compared with sgScramble controls, SMARCAL1-KO A549 cells exhibited significantly higher viability (78 ± 6% versus 31 ± 4%; Δ +151%, *P* < 0.001), reduced lipid peroxidation (BODIPY-C11 oxidized/reduced ratio 1.6 ± 0.3 versus 2.8 ± 0.4; Δ −43%, *P* < 0.001), lower intracellular Fe²^+^ levels (FerroOrange mean fluorescence intensity 3,210 ± 260 versus 4,820 ± 380; Δ −33%, *P* < 0.001), decreased malondialdehyde (7.9 ± 1.4 versus 14.2 ± 2.1 μM; Δ −44%, *P* < 0.001), and elevated reduced glutathione (0.94 ± 0.11 versus 0.62 ± 0.09 relative units; Δ +52%, *P* < 0.001). Parallel reductions in ferroptotic phenotypes were observed in H1299 cells (BODIPY-C11 oxidation decreased by 38 ± 9%, *P* < 0.001) (**Figure 10a**). Transmission electron microscopy confirmed fewer cells with characteristic ferroptotic mitochondrial morphology (shrunken mitochondria with increased membrane density) in SMARCAL1-KO cells (34% versus 71% in sgScramble, *P* < 0.001). These ferroptosis-resistant phenotypes were fully rescued by wild-type SMARCAL1 (viability 34 ± 5%) but not by the helicase-dead R764Q mutant (viability 74 ± 7%), indicating that helicase activity is mechanistically required (**Figure 10b** rescue panel; **Supplementary Figure 9**). In parallel, IFN-γ-stimulated chemokine secretion was significantly impaired in SMARCAL1-KO cells: CXCL9 protein levels decreased from 1,240 ± 180 pg/mL to 471 ± 94 pg/mL (Δ −62%, *P* < 0.001) and CXCL10 from 842 ± 126 pg/mL to 362 ± 71 pg/mL (Δ −57%, *P* < 0.001) (**Figure 10c**), with corresponding reductions in mRNA expression (CXCL9, 10.1 ± 2.0 versus 28.4 ± 3.6; CXCL10 9.2 ± 1.8 versus 22.1 ± 2.9; both *P* < 0.001). Chemokine defects were completely rescued by wild-type SMARCAL1 reconstitution and partially rescued (45% efficiency, *P* < 0.05) by the helicase-dead mutant, suggesting both helicase-dependent and -independent regulatory roles. Western blot analysis further revealed that SMARCAL1 knockout attenuated IFN-γ-induced cGAS-STING pathway activation, with marked reductions in phospho-STING (Ser366; 1.5 ± 0.3 versus 4.7 ± 0.6 AU, *P* < 0.001), phospho-TBK1 (Ser172; 1.4 ± 0.3 versus 3.9 ± 0.5 AU, *P* < 0.001), and phospho-IRF3 (Ser396; 1.5 ± 0.3 versus 5.2 ± 0.7 AU, *P* < 0.001)(**Figure 10d**). Forced STING activation with the agonist diABZI (10 μM, 6 h) partially restored CXCL9/10 secretion in SMARCAL1-KO cells (64% recovery for CXCL9 and 58% for CXCL10), establishing cGAS-STING signaling as a key mechanistic intermediate linking SMARCAL1 loss to impaired chemokine induction.

#### 3.9.4 Functional T cell chemotaxis demonstrates a causal axis

Functional T cell chemotaxis assays using primary human CD8^+^ T cells (activated with anti-CD3/CD28 Dynabeads to achieve >85% CXCR3 expression) isolated from three independent healthy donors demonstrated a direct causal link between tumor-cell SMARCAL1 expression and effector T cell recruitment (**Figure 10e**). In Transwell chambers, IFN-γ–stimulated supernatants from sgScramble A549 cells induced robust transmigration of CXCR3^+^ CD8^+^ T cells (9.8 ± 1.2 × 10³ cells, *P* < 0.001). In contrast, supernatants from IFN-γ–stimulated SMARCAL1-KO A549 cells produced a 58 ± 9% reduction in transmigrated cells (4.1 ± 0.8 × 10³ cells, *P* < 0.001 versus sgScramble). Addition of the CXCR3 antagonist AMG 487 (1 μM) to sgScramble supernatant fully abrogated chemotaxis (0.9 ± 0.2 × 10³ cells, *P* < 0.001). Parallel results were observed in H1299 cells (52 ± 11% reduction with SMARCAL1 knockout, *P* < 0.001). These functional data establish that SMARCAL1 expression in tumor cells is required for efficient CXCL9/10-driven recruitment of CXCR3^+^ CD8^+^ effector T cells. **Figure 10f** integrates all orthogonal validation layers into a unified mechanistic model: (1) replication stress within genomically unstable, APOBEC-enriched, High Rad-Score tumors drives SMARCAL1 upregulation to resolve stalled replication forks (transcriptomic/spatial evidence); (2) SMARCAL1 activity cooperates with ACSL4-mediated phospholipid remodeling to confer ferroptotic susceptibility (CRISPR ferroptosis assays); (3) chronic replication stress in SMARCAL1-high tumors activates the cGAS-STING-IRF3 axis, amplifying IFN-γ-induced CXCL9/10 transcription (Western blot and qRT-PCR); (4) CXCL9/10 chemokines create a gradient that recruits CXCR3^+^ CD8^+^ effector T cells to the peritumoral invasive margin (spatial ligand–receptor, cell2location, and Transwell chemotaxis); and (5) pre-operative peritumoral CD8^+^ infiltration primes systemic immune surveillance, providing the biological substrate for sustained postoperative MRD negativity.

## 4 Discussion

This is a preplanned translational cohort analysis nested within the prospective CTONG 2201 trial, focused on the 71 patients enrolled at the First Affiliated Hospital of Guangxi Medical University who maintained longitudinal MRD negativity and underwent dynamic observation without immediate adjuvant therapy. While single-center in scope, this design enabled the integrated multi-omics characterization required to address the biological basis of sustained MRD negativity, which could not be performed without the prospectively collected tissue samples. Within this cohort, we identified a pre-operative CT-derived radiogenomic signature (Rad-Score) that was associated with sustained MRD-negative surveillance and corresponded to an inflamed, ferroptosis-associated tumor microenvironment (21).

Medical imaging contains high-dimensional spatial information that may reflect underlying tumor biology (22). Prior radiomic models in NSCLC primarily predicted overall survival or stage and often treated tumors as isolated mathematical objects (23). By extracting features from both the intratumoral core (*ROI*_core_) and a 3-mm peritumoral invasive margin (*ROI*_rim_), our model captures gradients relevant to immune infiltration, neoangiogenesis, and local invasion (24). Using LASSO Cox regression, 1,432 features were reduced to 9 highly reproducible spatial features forming the Rad-Score, which was associated with reduced hazard of MRD conversion (HR = 0.32, 95% CI 0.18–0.58) and outperformed TNM stage in time-dependent ROC analyses. These findings suggest that pre-operative CT captures macro-architectural properties reflecting potential for post-operative systemic immune surveillance.

To interpret the Rad-Score biologically, we integrated multi-omics analyses. Elevated APOBEC signatures (*SBS2, SBS13*) and tumor mutational burden in High Rad-Score tumors suggest that replication stress-driven genomic instability generates the neoantigen diversity fueling the immune-active phenotype we observed. This aligns with the recent model of APOBEC-immunogenicity coupling described in advanced NSCLC (25, 26) and extends it to the early-stage, MRD-negative setting (26). These results suggest that radiomic heterogeneity reflects underlying clonal complexity and immunogenic potential even in early-stage, resectable tumors. In addition, SMARCAL1 expression was consistently higher in High Rad-Score tumors and co-expressed with ferroptosis-related genes (e.g., *ACSL4, PTGS2*) (27–29). Ferroptosis is an iron-dependent form of regulated cell death that can release damage-associated molecular patterns (DAMPs), promoting dendritic cell activation and CD8^+^ T cell priming (30, 31). Bulk RNA-seq deconvolution demonstrated significant enrichment of CD8+ effector T cells (q = 6.8 × 10^-5^) and M1 macrophages (q = 9.2 × 10^-^³) in High Rad-Score tumors under family-wide BH-FDR correction. A nominal but non-significant trend toward Treg enrichment in Low Rad-Score tumors (q = 0.170) did not survive multiple testing correction and remains exploratory. These associations are consistent with the hypothesis that the Rad-Score reflects tumor-intrinsic ferroptotic activity and immune engagement.

The most pivotal mechanistic insight emerging from our study is the association between the pre-operative Rad-Score, *SMARCAL1* expression, and the establishment of a ferroptosis-enriched, immune-active tumor microenvironment. *SMARCAL1* is an annealing helicase that maintains genomic stability by remodeling stalled DNA replication forks during replication stress (8). Tumors exhibiting high genomic instability, such as the *APOBEC*-enriched High Rad-Score cohort in our analysis, are likely to experience substantial replication stress and may rely on *SMARCAL1* to mitigate DNA damage (28). Transcriptomic analyses indicate that this dependency is accompanied by a coordinated transcriptional program associated with ferroptotic susceptibility. Specifically, *SMARCAL1* -high tumors displayed co-expression with canonical ferroptosis regulators, including *ACSL4* (Acyl-CoA Synthetase Long Chain Family Member 4) and *PTGS2*, suggesting a cellular state poised for iron-dependent, lipid peroxidation-mediated cell death (29). Ferroptosis has recently been recognized not solely as a mechanism of tumor cell elimination but also as an immunologically active process capable of releasing damage-associated molecular patterns (DAMPs) that can prime dendritic cells and enhance cytotoxic CD8^+^ T cell responses (30, 31). Our bulk RNA-seq deconvolution supports this paradigm: High Rad-Score tumors were associated with substantial infiltration of CD8^+^ T cells and M1 macrophages, whereas Low Rad-Score tumors were enriched for regulatory T cells, indicative of a comparatively immunosuppressive microenvironment. Consistent with this interpretation, CD8+ T cell fractions were approximately 2-fold higher in High Rad-Score tumors (22.1% vs 11.3%, q = 6.8 × 10^-5^) and M1 macrophage fractions similarly doubled (17.6% vs 9.8%, q = 9.2 × 10^-^³). This Th1-skewed macrophage polarization, together with the CD8+ expansion, constitutes a coordinated immune-hot phenotype that may underlie the durable MRD-negative status observed in this subgroup.

Recognizing that bulk multi-omics analyses provide associative evidence without spatial resolution, we further leveraged 10x Visium spatial transcriptomics to examine the three-dimensional organization of SMARCAL1 and ferroptosis-related activity within the tumor ecosystem (32). High Rad-Score tumors displayed contiguous regions of elevated ferroptosis-related gene expression confined to dense tumor nests, whereas Low Rad-Score tumors lacked such regions. Across all 36,318 quality-controlled spatial spots, the per-patient median Ferroptosis Activity Score was significantly higher in High Rad-Score tumors compared with Low Rad-Score tumors (median 0.87 vs 0.21; BH-FDR q < 0.001), supporting the hypothesis that the Rad-Score reflects spatially localized ferroptotic programs rather than random cell death. This spatial organization is functionally relevant, as isolated apoptotic or necrotic events in tumors often fail to generate sufficient chemokine gradients to recruit immune effectors (33). To investigate the mechanisms underlying immune recruitment, we applied spatial deconvolution and ligand–receptor inference. High Rad-Score tumors demonstrated a robust CXCL9/10–CXCR3 signaling axis (**Figure 7C**), with ferroptotic tumor cores and tumor-associated macrophages as the principal chemokine sources and the peritumoral invasive margin enriched for CXCR3^+^ CD8^+^ T cells (**Figure 7a**). CRISPR-Cas9 loss-of-function validation directly confirmed SMARCAL1 as a causal driver of CXCL9/10 induction in tumor cells (62% and 57% reduction respectively; **Figure 10c**), placing this axis on mechanistic rather than merely correlative ground. The strong spatial co-localization between per-spot ferroptosis activity and adjacent CD8+ T cell density (Pearson R = 0.76, 95% CI 0.68-0.83) is biologically consistent with the ‘ferroptosis-releases-DAMPs-to-prime-dendritic-cells-and-T-cells’ model proposed in preclinical studies (10, 30, 31). Our data extend this model to human early-stage NSCLC at cellular spatial resolution and propose that such *in situ* immune engagement pre-operatively may shape the trajectory of systemic immune surveillance post-operatively. This suggests that the ferroptotic core may provide chemotactic cues that facilitate the accumulation of effector T cells at the tumor margin, potentially priming systemic immune surveillance that persists post-resection (34).

The Rad-Score’s translational relevance is further highlighted by its integration into a multimodal nomogram alongside age, pathological TNM stage, and TMB status. Internal validation following Harrell optimism-correction with full-pipeline resampling (B = 1,000) yielded optimism-corrected calibration slope 0.94 (95% CI 0.82-1.06) and intercept −0.08 (95% CI −0.19 to +0.03), consistent with good calibration across the 1-year and 2-year prediction horizons. Decision Curve Analysis demonstrated superior net clinical benefit of the multimodal nomogram compared with a clinical-only model or default treat-all/treat-none strategies across a wide range of threshold probabilities (0.15–0.75; **Figure 8c**). For instance, within this cohort, patients with the dichotomized High Rad-Score had a 24-month MFS of 38%, compared to 82% for patients with the Low score, highlighting the potential stratification capacity of the Rad-Score. Importantly, while these findings provide exploratory evidence for risk stratification, clinical implementation must remain cautious. Low Rad-Score patients may represent a biologically ‘cold’ tumor state with attenuated ferroptosis-associated immune priming. For this subgroup, closer longitudinal MRD monitoring and/or enrollment in biomarker-stratified early adjuvant immunotherapy trials could be considered in future studies. While universal adjuvant atezolizumab demonstrated MFS benefit in IMpower010 (35), biomarker-stratified adjuvant approaches leveraging pre-operative Rad-Score status remain untested and represent a hypothesis-generating direction for prospective trials such as the proposed LOW-RAD-ADJUVANT design. Conversely, high Rad-Score patients may represent a lower-risk subgroup suitable for routine observation pending independent validation (36). Thus, the Rad-Score and the associated nomogram currently serve as hypothesis-generating tools that integrate spatial, genomic, and radiologic information to inform future prospective and multicenter studies. The LOW-RAD-ADJUVANT trial is being designed to prospectively test whether Rad-Score stratification can guide early adjuvant immunotherapy versus observation in MRD-negative patients.

## 5 Limitations

Several limitations of the present study should be acknowledged. First, this analysis represents a single-center translational cohort derived from one participating site of the prospective CTONG 2201 study, with a final analytical sample of 71 patients and 26 events. The minimum sample size calculation for the nine-parameter Cox model (n = 268, ≥ 89 events) is not met by the discovery cohort; accordingly, the Rad-Score is framed as an exploratory signature requiring prospective multicenter validation, and a uniform shrinkage factor (γ = 0.84) has been applied to the final linear predictor. Internal validation followed Harrell optimism-correction with full-pipeline resampling (B = 1,000), but external validation remains essential before clinical deployment. Second, the radiogenomic signature was developed from retrospectively analyzed imaging and tissue data collected within a prospectively observed framework. Although image preprocessing—including isotropic resampling, Z-score normalization, and ComBat harmonization with parameters frozen from training and applied to external cohorts—was applied to reduce scanner- and manufacturer-specific variability, residual acquisition differences may influence feature reproducibility. Third, while spatial transcriptomic profiling was expanded to 14 tumors spanning the full Rad-Score continuum (5 High, 4 Intermediate, 5 Low tiers; 36,318 quality-controlled spots), the Visium platform has 55-μm spatial resolution, which averages signals from 1–10 cells per spot; single-cell spatial transcriptomic platforms (e.g., Xenium, MERFISH) in future studies will permit finer cellular resolution of the ferroptotic niche. Fourth, although the SMARCAL1–ferroptosis–CXCL9/10–CXCR3 axis has been validated at the protein level via multiplex immunofluorescence (**Figure 9**) and causally established via CRISPR-Cas9 loss-of-function with wild-type and helicase-dead rescue (**Figure 10**), these experiments were performed in NSCLC cell lines; *in vivo* validation in syngeneic mouse models with SMARCAL1-KO tumors and CD8+ T cell depletion remains to be performed. Fifth, because our clinical cohort is observational, the association between Rad-Score and sustained MRD-negative status does not prove that Rad-Score-guided clinical decisions would improve outcomes; prospective interventional validation in the planned LOW-RAD-ADJUVANT trial will address this question. Sixth, the external validation cohorts (TCIA NSCLC-Radiogenomics, n = 178; Lung3, n = 89) used radiographic recurrence or overall survival as endpoints because pre-operative ctDNA MRD assessment was not available retrospectively—this endpoint difference is a partial departure from the training cohort’s MRD-Free Survival endpoint, though directional and magnitude findings were consistent (37–39). Seventh, the Rad-Score dichotomization cut-off was determined using maximally selected rank statistics, a data-driven approach that can inflate type I error; the continuous Rad-Score was therefore reported in parallel as a sensitivity check throughout. Finally, while the CRISPR-Cas9 experiments establish causality for the SMARCAL1 → CXCL9/10 → CXCR3+ CD8+ recruitment arm *in vitro*, extrapolation to *in vivo* tumor-immune surveillance remains to be formally tested.

## 6 Conclusions

In conclusion, this study establishes a framework linking macroscopic pre-operative CT imaging features to microscopic spatial transcriptomic architecture in early-stage NSCLC. We demonstrate that a non-invasive, continuous Rad-Score accurately reflects the presence of a SMARCAL1-enriched, ferroptosis-associated, immune-active niche within the primary tumor core. High Rad-Score tumors are associated with elevated TMB, APOBEC mutational signatures, and enriched CD8+ T cell and M1 macrophage infiltration, as well as spatially concentrated ferroptotic activity and CXCL9/10–CXCR3-mediated immune recruitment. Integration of the Rad-Score with age, pathological TNM stage, and TMB status into a multimodal nomogram supports individualized-risk estimation for 1- and 2-year MRD-free survival in this exploratory cohort, with internal validation demonstrating adequate calibration and superior net clinical benefit relative to clinical-only models across a clinically meaningful threshold range. Prospective external validation in larger multicenter cohorts is required before clinical deployment. These findings provide hypothesis-generating evidence that spatially resolved radiogenomic features can inform risk stratification and potentially guide post-operative dynamic observation protocols.

## Data Availability

The original contributions presented in the study are included in the article/**Supplementary Material**. Further inquiries can be directed to the corresponding authors.

## References

[1] Goldstraw P Chansky K Crowley J Rami-Porta R Asamura H Eberhardt WE . The IASLC Lung Cancer Staging Project: proposals for revision of the TNM stage groupings in the forthcoming (eighth) edition of the TNM classification for lung cancer. J Thorac Oncol. (2016) 11:39–51. doi:10.1016/j.jtho.2015.09.009. PMID: 26762738

[2] Pignon JP Tribodet H Scagliotti GV Douillard JY Shepherd FA Stephens RJ . Lung adjuvant cisplatin evaluation: a pooled analysis by the LACE Collaborative Group. J Clin Oncol. (2008) 26:3552–9. doi:10.1200/jco.2007.13.9030. PMID: 18506026

[3] Verzè M Pluchino M Leonetti A Corianò M Bonatti F Armillotta MP . Residual disease detected by circulating tumor DNA in completely resected early-stage non-small cell lung cancer. Ann Oncol. (2022) 11:2588–600. doi: 10.21037/tlcr-22-390 PMC9830273 36636413

[4] Roulleaux Dugage M Albarrán-Artahona V Laguna JC Chaput N Vignot S Besse B . Biomarkers of response to immunotherapy in early stage non-small cell lung cancer. Eur J Cancer. (2023) 184:179–96. doi: 10.1016/j.ejca.2023.01.029 36963241

[5] Galon J Bruni D . Approaches to treat immune hot, altered and cold tumours with combination immunotherapies. Nat Rev Drug Discov. (2019) 18:197–218. doi:10.1038/s41573-018-0007-y. PMID: 30610226

[6] Wang W Green M Choi JE Gijón M Kennedy PD Johnson JK . CD8+ T cells regulate tumour ferroptosis during cancer immunotherapy. Nature. (2019) 569:270–4. doi:10.1038/s41586-019-1170-y. PMID: 31043744 PMC6533917

[7] Stockwell BR Friedmann Angeli JP Bayir H Bush AI Conrad M Dixon SJ . Ferroptosis: a regulated cell death nexus linking metabolism, redox biology, and disease. Cell. (2017) 171:273–85. doi:10.1016/j.cell.2017.09.021. PMID: 28985560 PMC5685180

[8] Bansbach CE Bétous R Lovejoy CA Glick GG Cortez D . The annealing helicase SMARCAL1 maintains genomic stability by rescuing stalled replication forks. Genes Dev. (2009) 23:2405–14. doi:10.1101/gad.1839909. PMID: 19793861 PMC2764496

[9] Bartek J Lukas J . DNA damage checkpoints: from initiation to recovery or adaptation. Curr Opin Cell Biol. (2007) 19:238–45. doi:10.1016/j.ceb.2007.02.009. PMID: 17303408

[10] Liao P Wang W Wang W Kryczek I Li X Bian Y . CD8+ T cells and fatty acids orchestrate tumor ferroptosis and immunity via ACSL4. Cancer Cell. (2022) 40:365–78. doi:10.1016/j.ccell.2022.02.003. PMID: 35216678 PMC9007863

[11] Massagué J Obenauf AC . Metastatic colonization by circulating tumour cells. Nature. (2016) 529:298–306. doi: 10.1038/nature17038 26791720 PMC5029466

[12] Gerlinger M Rowan AJ Horswell S Math M Larkin J Endesfelder D . Intratumor heterogeneity and branched evolution revealed by multiregion sequencing. N Engl J Med. (2012) 366:883–92. doi:10.1056/nejmoa1113205. PMID: 22397650 PMC4878653

[13] Lambin P Leijenaar RTH Deist TM Peerlings J de Jong EEC van Timmeren J . Radiomics: the bridge between medical imaging and personalized medicine. Nat Rev Clin Oncol. (2017) 14:749–62. doi:10.1038/nrclinonc.2017.141. PMID: 28975929

[14] Bi WL Hosny A Schabath MB Giger ML Birkbak NJ Mehrtash A . Artificial intelligence in cancer imaging: clinical challenges and applications. CA Cancer J Clin. (2019) 69:127–57. doi:10.3322/caac.21552. PMID: 30720861 PMC6403009

[15] Bera K Schalper KA Rimm DL Velcheti V Madabhushi A . Artificial intelligence in digital pathology—new tools for diagnosis and precision oncology. Nat Rev Clin Oncol. (2019) 16:703–15. doi:10.1038/s41571-019-0252-y. PMID: 31399699 PMC6880861

[16] Denis MG Herbreteau G Pons-Tostivint E . Molecular minimal residual disease in resected non-small cell lung cancer (NSCLC): results of specifically designed interventional clinical trials eagerly awaited. Transl Lung Cancer Res. (2023) 12:200–3. doi:10.21037/tlcr-22-899. PMID: 36895928 PMC9989810

[17] Li HJ Qiu ZB Wang MM Zhang C Hong HZ Fu R . Radiomics-based support vector machine distinguishes molecular events driving the progression of lung adenocarcinoma. J Thorac Oncol. (2025) 20:52–64. doi:10.1016/j.jtho.2024.09.1431. PMID: 39306192

[18] Newman AM Steen CB Liu CL Gentles AJ Chaudhuri AA Scherer F . Determining cell type abundance and expression from bulk tissues with digital cytometry. Nat Biotechnol. (2019) 37:773–82. doi:10.1038/s41587-019-0114-2. PMID: 31061481 PMC6610714

[19] Ståhl PL Salmén F Vickovic S Lundmark A Navarro JF Magnusson J . Visualization and analysis of gene expression in tissue sections by spatial transcriptomics. Science. (2016) 353:78–82. doi: 10.1126/science.aaf2403 27365449

[20] Jin S Guerrero-Juarez CF Zhang L Chang I Ramos R Kuan CH . Inference and analysis of cell-cell communication using CellChat. Nat Commun. (2021) 12:1088. doi:10.1038/s41467-021-21246-9. PMID: 33597522 PMC7889871

[21] Chabon JJ Hamilton EG Kurtz DM Esfahani MS Moding EJ Stehr H . Integrating genomic features for non-invasive early lung cancer detection. Nature. (2020) 580:245–52. doi:10.1038/s41586-020-2140-0. PMID: 32269342 PMC8230734

[22] Aerts HJ Velazquez ER Leijenaar RT Parmar C Grossmann P Carvalho S . Decoding tumour phenotype by noninvasive imaging using a quantitative radiomics approach. Nat Commun. (2014) 5:4006. doi:10.1038/ncomms5006. PMID: 24892406 PMC4059926

[23] Hosny A Parmar C Coroller TP Grossmann P Zeleznik R Kumar A . Deep learning for lung cancer prognostication: a retrospective large cohort study. PloS Med. (2018) 15:e1002711. doi:10.1371/journal.pmed.1002711. PMID: 30500819 PMC6269088

[24] Yu Y He Z Ouyang J Tan Y Chen Y Gu Y . Magnetic resonance imaging radiomics predicts preoperative axillary lymph node metastasis to support surgical decisions and is associated with tumor immune microenvironment in invasive breast cancer: a machine learning, multicenter study. EBioMedicine. (2021) 69:103460. doi:10.1016/j.ebiom.2021.103460. PMID: 34233259 PMC8261009

[25] Swanton C McGranahan N Starrett GJ Harris RS . APOBEC enzymes: mutagenic fuel for cancer evolution and targeted therapies. Cancer Discov. (2015) 5:704–12. doi:10.1158/2159-8290.cd-15-0344. PMID: 26091828 PMC4497973

[26] Wang S Jia M He Z Liu XS . APOBEC3B and APOBEC mutational signature as potential predictive markers for immunotherapy response in non-small cell lung cancer. Oncogene. (2018) 37:3924–36. doi:10.1038/s41388-018-0245-9. PMID: 29695832 PMC6053356

[27] Poole LA Zhao R Glick GG Lovejoy CA Eischen CM Cortez D . SMARCAL1 maintains telomere integrity during DNA replication. Proc Natl Acad Sci U S A. (2015) 112:14864–9. doi: 10.1073/pnas.1510750112 PMC4672769 26578802

[28] Poole LA Cortez D . Functions of SMARCAL1, ZRANB3, and HLTF in maintaining genome stability. Crit Rev Biochem Mol Biol. (2017) 52:696–714. doi:10.1080/10409238.2017.1380597. PMID: 28954549 PMC5962345

[29] Dixon SJ Lemberg KM Lamprecht MR Skouta R Zaitsev EM Gleason CE . Ferroptosis: an iron-dependent form of nonapoptotic cell death. Cell. (2012) 149:1060–72. doi:10.1016/j.cell.2012.03.042. PMID: 22632970 PMC3367386

[30] Friedmann Angeli JP Krysko DV Conrad M . Ferroptosis at the crossroads of cancer-acquired drug resistance and immune evasion. Nat Rev Cancer. (2019) 19:405–14. doi: 10.1038/s41568-019-0149-1 31101865

[31] Tang R Xu J Zhang B Liu J Liang C Hua J . Ferroptosis, necroptosis, and pyroptosis in anticancer immunity. J Hematol Oncol. (2020) 13:110. doi:10.1186/s13045-020-00946-7. PMID: 32778143 PMC7418434

[32] Marx V . Method of the Year: spatially resolved transcriptomics. Nat Methods. (2021) 18:9–14. doi:10.1038/s41592-020-01033-y. PMID: 33408395

[33] Pickard K Stephenson E Mitchell A Jardine L Bacon CM . Location, location, location: mapping the lymphoma tumor microenvironment using spatial transcriptomics. Front Oncol. (2023) 13:1258245. doi:10.3389/fonc.2023.1258245. PMID: 37869076 PMC10586500

[34] Tokunaga R Zhang W Naseem M Puccini A Berger MD Soni S . CXCL9, CXCL10, CXCL11/CXCR3 axis for immune activation - a target for novel cancer therapy. Cancer Treat Rev. (2018) 63:40–7. doi:10.1016/j.ctrv.2017.11.007. PMID: 29207310 PMC5801162

[35] Felip E Altorki N Zhou C Csőszi T Vynnychenko I Goloborodko O . Adjuvant atezolizumab after adjuvant chemotherapy in resected stage IB-IIIA non-small-cell lung cancer (IMpower010): a randomised, multicentre, open-label, phase 3 trial. Lancet. (2021) 398:1344–57. doi:10.1016/s0140-6736(21)02098-5. PMID: 34555333

[36] Forde PM Spicer J Lu S Provencio M Mitsudomi T Awad MM . Neoadjuvant nivolumab plus chemotherapy in resectable lung cancer. N Engl J Med. (2022) 386:1973–85. doi:10.1056/nejmoa2202170. PMID: 35403841 PMC9844511

[37] Moding EJ Nabet BY Alizadeh AA Diehn M . Detecting liquid remnants of solid tumors: circulating tumor DNA minimal residual disease. Cancer Discov. (2021) 11:2968–86. doi:10.1158/2159-8290.cd-21-0634. PMID: 34785539 PMC8976700

[38] Zhang J Ji Z Caushi JX El Asmar M Anagnostou V Cottrell TR . Compartmental analysis of T-cell clonal dynamics as a function of pathologic response to neoadjuvant PD-1 blockade in resectable non-small cell lung cancer. Clin Cancer Res. (2020) 26:1327–37. doi:10.1158/1078-0432.ccr-19-2931. PMID: 31754049 PMC7073288

[39] Travis WD Brambilla E Nicholson AG Yatabe Y Austin JHM Beasley MB . The 2015 World Health Organization classification of lung tumors: impact of genetic, clinical and radiologic advances since the 2004 classification. J Thorac Oncol. (2015) 10:1243–60. doi:10.1097/JTO.0000000000000630. PMID: 26291008

