# Supplementary material for: Non-invasive radiogenomic mapping of the SMARCAL1-driven ferroptotic niche is associated with longitudinal MRD-negative surveillance in early-stage NSCLC
Source: Front Immunol. 2026 May 25;17:1835413. doi: 10.3389/fimmu.2026.1835413 (PMC13243389; doi:10.3389/fimmu.2026.1835413)

figure s1

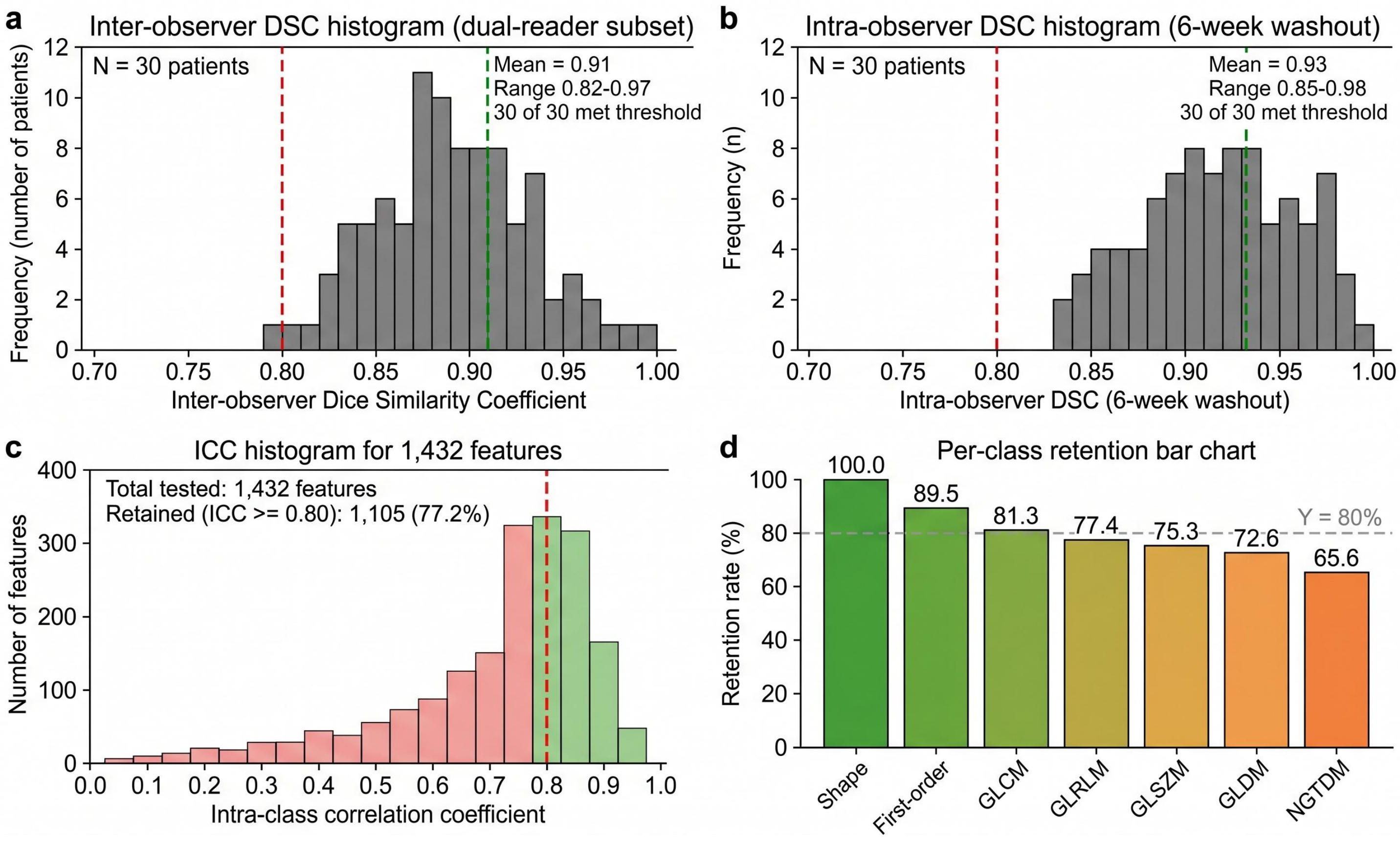
a
Inter-observer DSC histogram (dual-reader subset)
N = 30 patients
Mean = 0.91
Range 0.82-0.97
30 of 30 met threshold
Frequency (number of patients)
Inter-observer Dice Similarity Coefficient
0.70 0.75 0.80 0.85 0.90 0.95 1.00
b
Intra-observer DSC histogram (6-week washout)
N = 30 patients
Mean = 0.93
Range 0.85-0.98
30 of 30 met threshold
Frequency (n)
Intra-observer DSC (6-week washout)
c
ICC histogram for 1,432 features
Total tested: 1,432 features
Retained (ICC >= 0.80): 1,105 (77.2%)
Number of features
Intra-class correlation coefficient
d
Per-class retention bar chart
100.0
89.5
81.3
77.4
75.3
72.6
65.6
Y = 80%
Retention rate (%)
Shape
First-order
GLCM
GLRLM
GLSZM
GLDM
NGTDM

figure s2

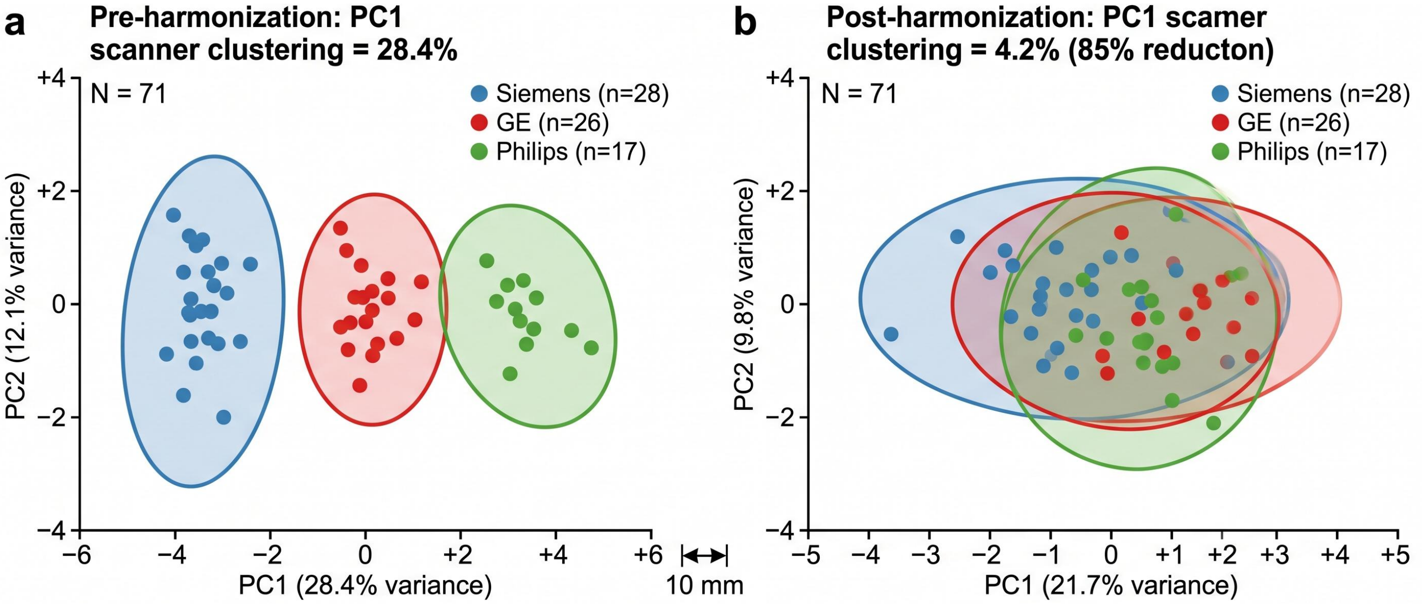
a
Pre-harmonization: PC1 scanner clustering = 28.4%
N = 71
Siemens (n=28)
GE (n=26)
Philips (n=17)
PC2 (12.1% variance)
PC1 (28.4% variance)
+4
+2
0
−2
−4
−6
−4
−2
0
+2
+4
+6
10 mm
b
Post-harmonization: PC1 scamer clustering = 4.2% (85% reducton)
N = 71
Siemens (n=28)
GE (n=26)
Philips (n=17)
PC2 (9.8% variance)
PC1 (21.7% variance)
−5
−4
−3
−2
−1
0
+1
+2
+3
+4
+5

figure s3

a

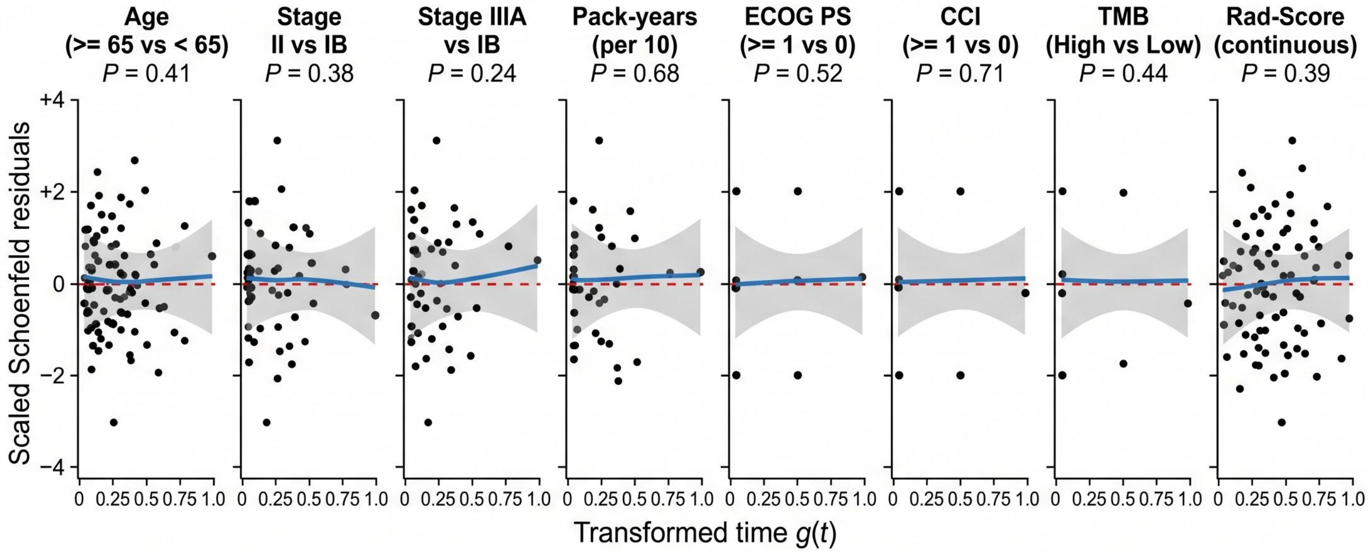

b

Covariate	Schoenfeld chi-square	*P* value	PH assumption met
Age (>= 65)	0.68	0.41	Yes
Stage II	0.77	0.38	Yes
Stage IIIA	1.38	0.24	Yes
Pack-years	0.17	0.68	Yes
ECOG PS	0.41	0.52	Yes
CCI	0.14	0.71	Yes
TMB	0.59	0.44	Yes
Rad-Score	0.74	0.39	Yes
**GLOBAL**	**6.18**	**0.51**	**Yes**

All $P > 0.10$; global $P = 0.51$; proportional hazards assumption satisfied

figure s4

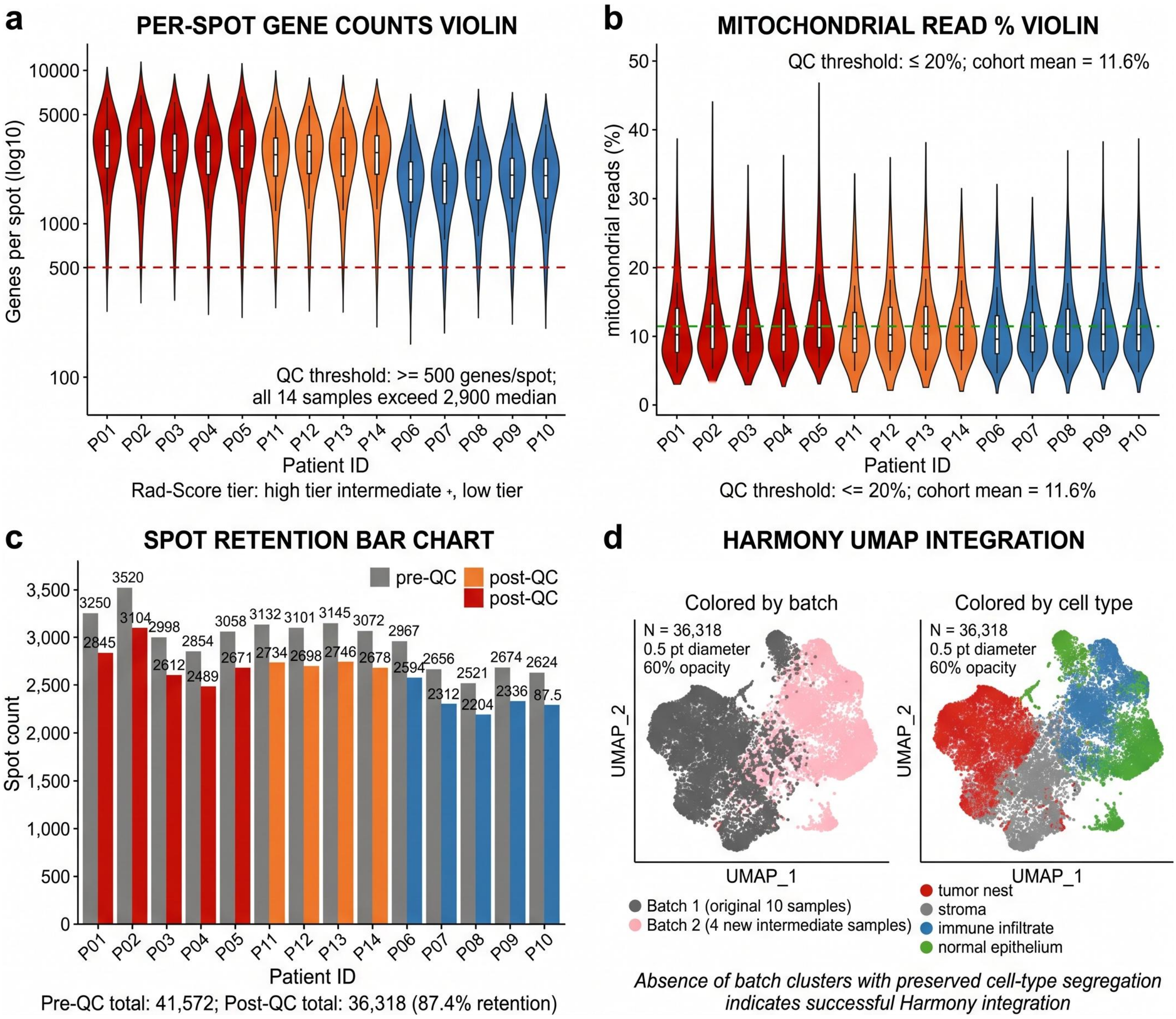

Absence of batch clusters with preserved cell-type segregation indicates successful Harmony integration

figure s5

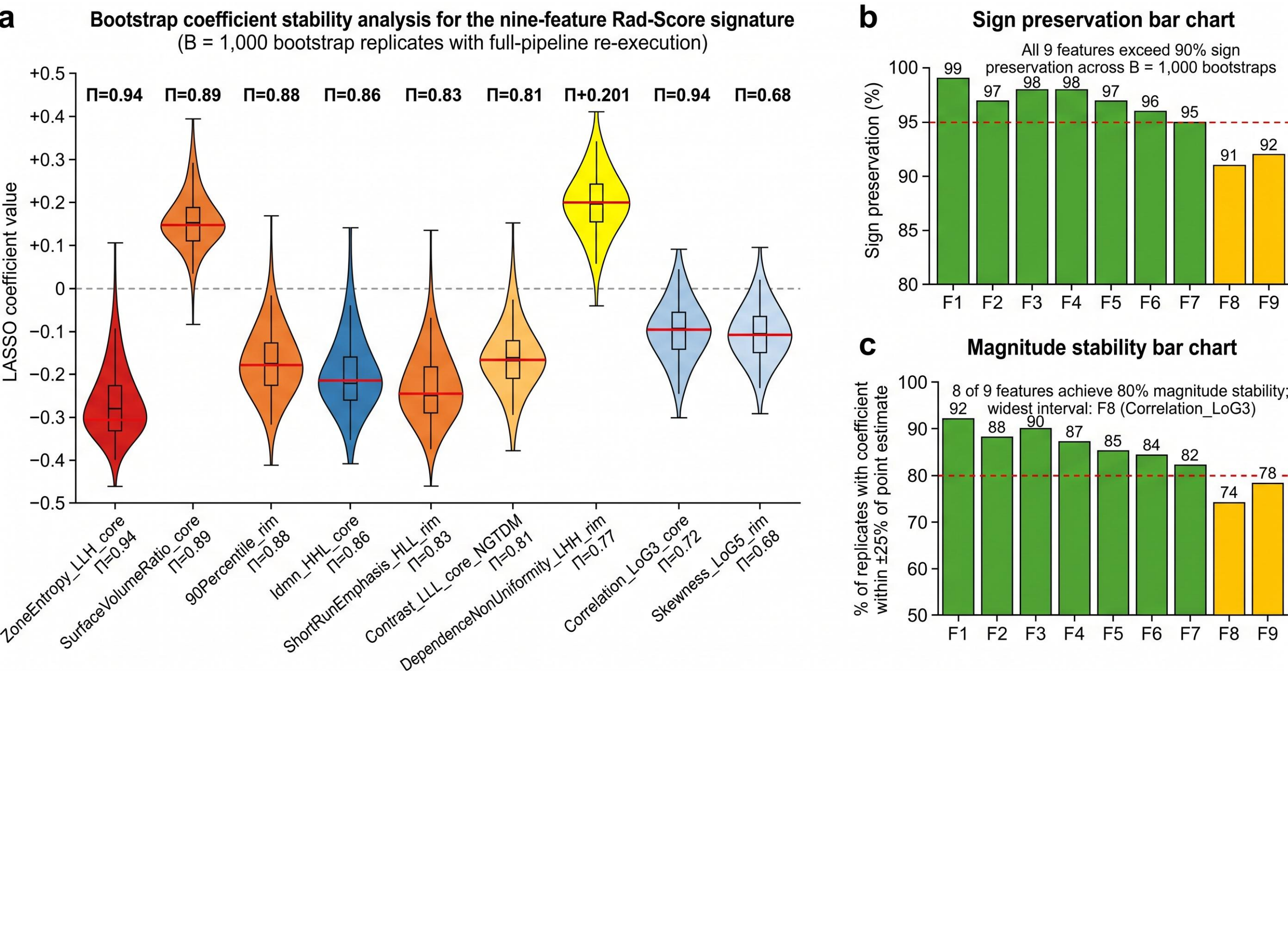
a
Bootstrap coefficient stability analysis for the nine-feature Rad-Score signature
(B = 1,000 bootstrap replicates with full-pipeline re-execution)
LASSO coefficient value
Π=0.94 Π=0.89 Π=0.88 Π=0.86 Π=0.83 Π=0.81 Π+0.201 Π=0.94 Π=0.68
ZoneEntropy_LLH_core Π=0.94
SurfaceVolumeRatio_core Π=0.89
90Percentile_rim Π=0.88
Idmn_HHL_core Π=0.86
ShortRunEmphasis_HLL_rim Π=0.83
Contrast_LLL_core_NGTDM Π=0.81
DependenceNonUniformity_LHH_rim Π=0.77
Correlation_LoG3_core Π=0.72
Skewness_LoG5_rim Π=0.68
b
Sign preservation bar chart
All 9 features exceed 90% sign preservation across B = 1,000 bootstraps
Sign preservation (%)
99 97 98 98 97 96 95 91 92
F1 F2 F3 F4 F5 F6 F7 F8 F9
c
Magnitude stability bar chart
8 of 9 features achieve 80% magnitude stability; widest interval: F8 (Correlation_LoG3)
% of replicates with coefficient within ±25% of point estimate
92 88 90 87 85 84 82 74 78
F1 F2 F3 F4 F5 F6 F7 F8 F9

figure s6

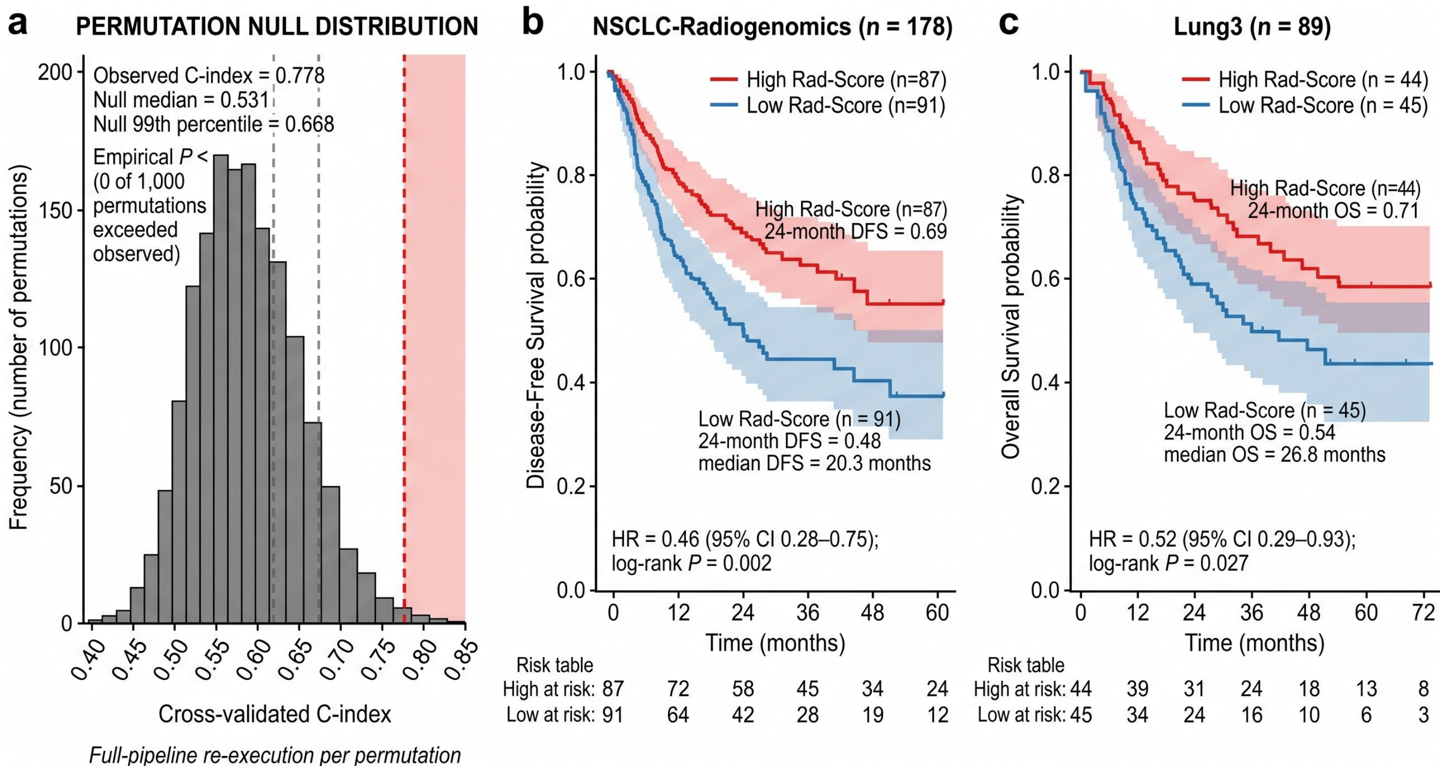
a
PERMUTATION NULL DISTRIBUTION
Observed C-index = 0.778
Null median = 0.531
Null 99th percentile = 0.668
Empirical P <
(0 of 1,000
permutations
exceeded
observed)
Frequency (number of permutations)
Cross-validated C-index
Full-pipeline re-execution per permutation
b
NSCLC-Radiogenomics (n = 178)
High Rad-Score (n=87)
Low Rad-Score (n=91)
High Rad-Score (n=87)
24-month DFS = 0.69
Low Rad-Score (n = 91)
24-month DFS = 0.48
median DFS = 20.3 months
HR = 0.46 (95% CI 0.28–0.75);
log-rank P = 0.002
Disease-Free Survival probability
Time (months)
Risk table
High at risk: 87 72 58 45 34 24
Low at risk: 91 64 42 28 19 12
c
Lung3 (n = 89)
High Rad-Score (n = 44)
Low Rad-Score (n = 45)
High Rad-Score (n=44)
24-month OS = 0.71
Low Rad-Score (n = 45)
24-month OS = 0.54
median OS = 26.8 months
HR = 0.52 (95% CI 0.29–0.93);
log-rank P = 0.027
Overall Survival probability
Time (months)
Risk table
High at risk: 44 39 31 24 18 13 8
Low at risk: 45 34 24 16 10 6 3

figure s7

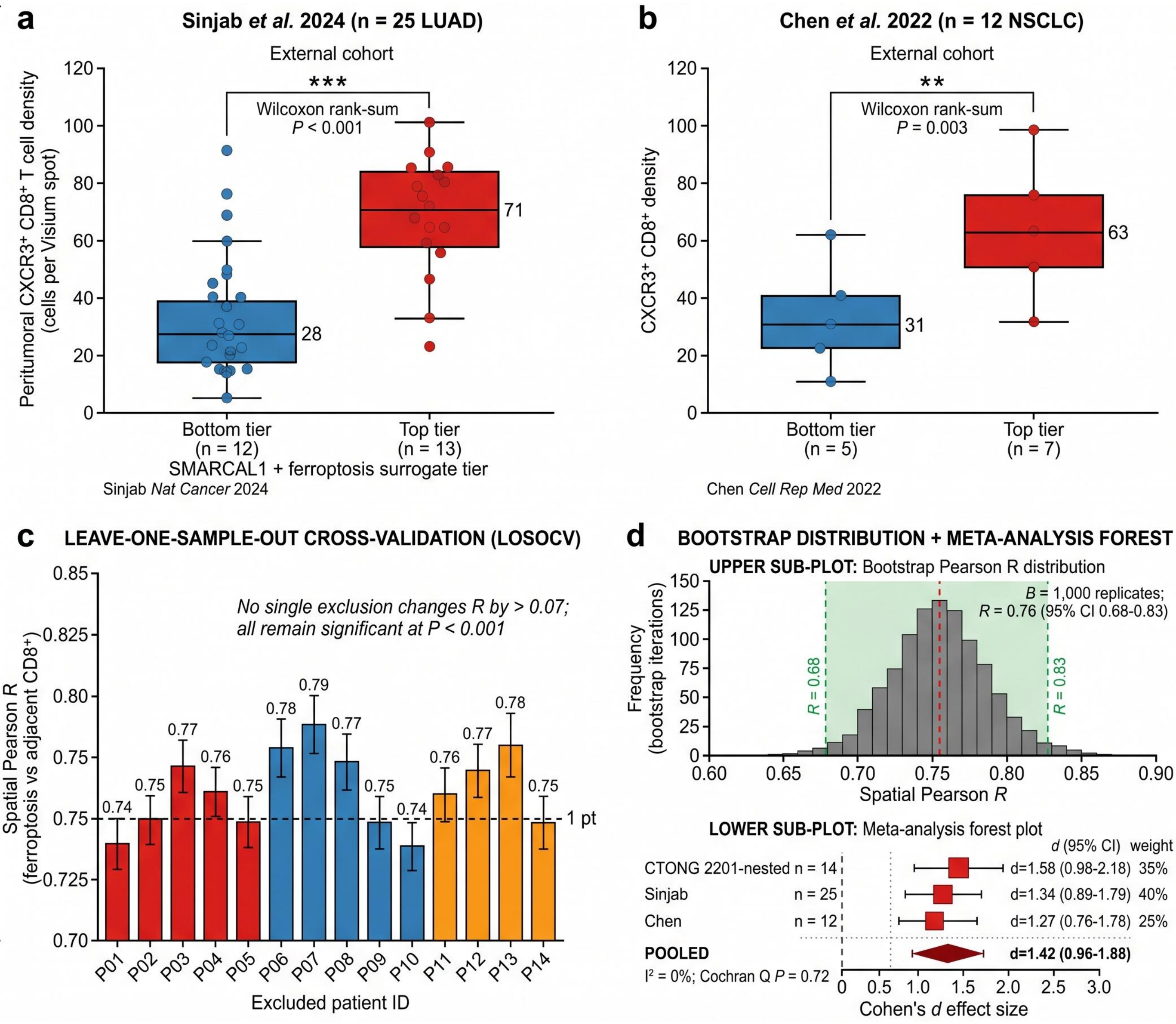

a
Sinjab et al. 2024 (n = 25 LUAD)
External cohort
Wilcoxon rank-sum
P < 0.001
Peritumoral CXCR3+ CD8+ T cell density (cells per Visium spot)
Bottom tier (n = 12)
Top tier (n = 13)
SMARCAL1 + ferroptosis surrogate tier
Sinjab Nat Cancer 2024
b
Chen et al. 2022 (n = 12 NSCLC)
External cohort
Wilcoxon rank-sum
P = 0.003
CXCR3+ CD8+ density
Bottom tier (n = 5)
Top tier (n = 7)
Chen Cell Rep Med 2022
c
LEAVE-ONE-SAMPLE-OUT CROSS-VALIDATION (LOSOCV)
No single exclusion changes R by > 0.07; all remain significant at P < 0.001
Spatial Pearson R (ferroptosis vs adjacent CD8+)
1 pt
Excluded patient ID
d
BOOTSTRAP DISTRIBUTION + META-ANALYSIS FOREST
UPPER SUB-PLOT: Bootstrap Pearson R distribution
B = 1,000 replicates; R = 0.76 (95% CI 0.68-0.83)
R = 0.68
R = 0.83
Frequency (bootstrap iterations)
Spatial Pearson R
LOWER SUB-PLOT: Meta-analysis forest plot
d (95% CI) weight
CTONG 2201-nested n = 14 d=1.58 (0.98-2.18) 35%
Sinjab n = 25 d=1.34 (0.89-1.79) 40%
Chen n = 12 d=1.27 (0.76-1.78) 25%
POOLED d=1.42 (0.96-1.88)
I² = 0%; Cochran Q P = 0.72
Cohen's d effect size

figure s8

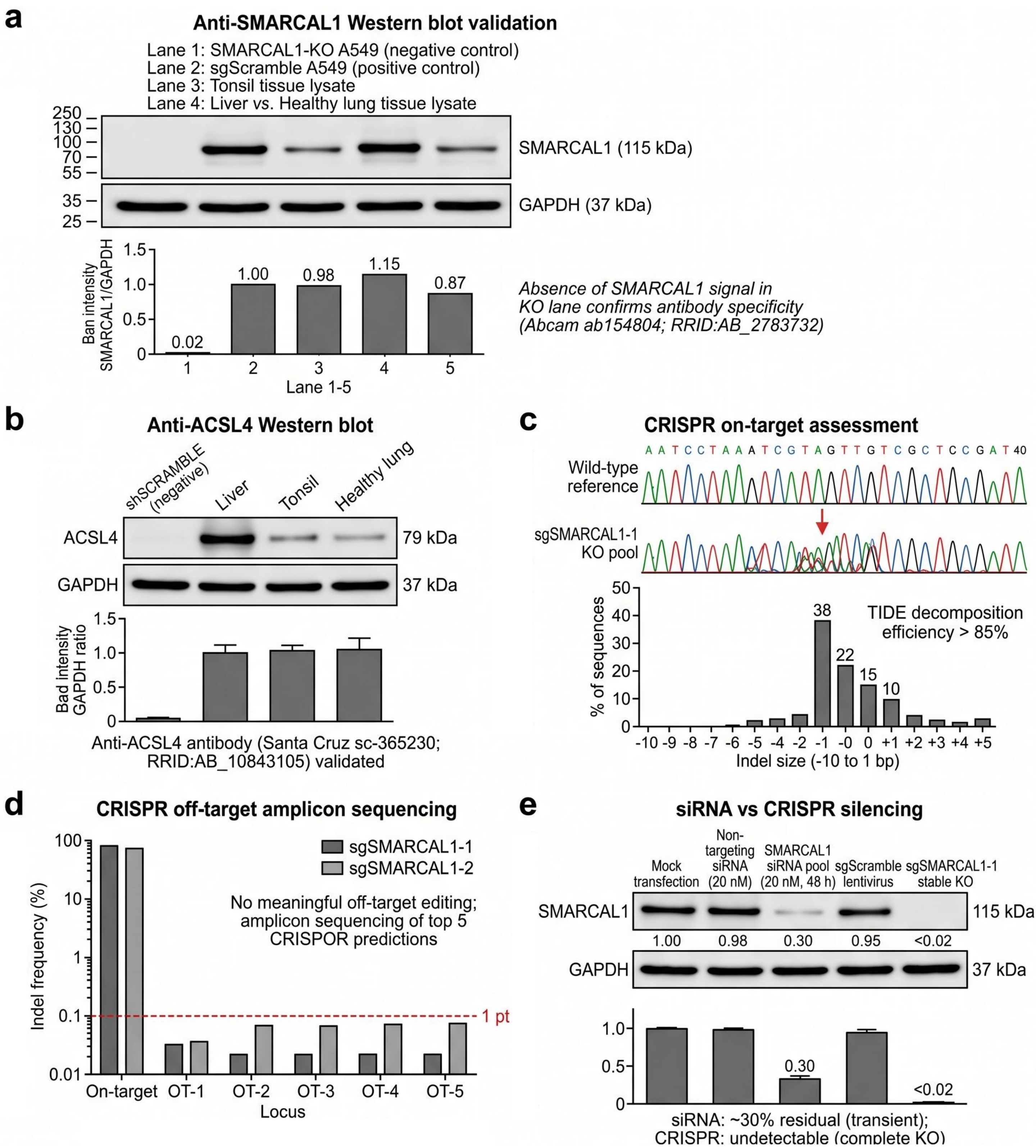

a
Anti-SMARCAL1 Western blot validation
Lane 1: SMARCAL1-KO A549 (negative control)
Lane 2: sgScramble A549 (positive control)
Lane 3: Tonsil tissue lysate
Lane 4: Liver vs. Healthy lung tissue lysate
250
130
100
70
55
35
25
SMARCAL1 (115 kDa)
GAPDH (37 kDa)
Ban intensity SMARCAL1/GAPDH
1.5
1.0
0.5
0
0.02
1.00
0.98
1.15
0.87
1
2
3
4
5
Lane 1-5
Absence of SMARCAL1 signal in KO lane confirms antibody specificity (Abcam ab154804; RRID:AB_2783732)
b
Anti-ACSL4 Western blot
shSCRAMBLE (negative)
Liver
Tonsil
Healthy lung
ACSL4
79 kDa
GAPDH
37 kDa
Bad intensity GAPDH ratio
1.5
1.0
0.5
0
Anti-ACSL4 antibody (Santa Cruz sc-365230; RRID:AB_10843105) validated
c
CRISPR on-target assessment
A A T C C T A A A T C G T A G T T G T C G C T C C G A T 40
Wild-type reference
sgSMARCAL1-1 KO pool
% of sequences
50
40
30
20
10
0
38
22
15
10
TIDE decomposition efficiency > 85%
-10 -9 -8 -7 -6 -5 -4 -2 -1 -0 0 +1 +2 +3 +4 +5
Indel size (-10 to 1 bp)
d
CRISPR off-target amplicon sequencing
Indel frequency (%)
100
10
1
0.1
0.01
sgSMARCAL1-1
sgSMARCAL1-2
No meaningful off-target editing; amplicon sequencing of top 5 CRISPOR predictions
1 pt
On-target
OT-1
OT-2
OT-3
OT-4
OT-5
Locus
e
siRNA vs CRISPR silencing
Mock transfection
Non-targeting siRNA (20 nM)
SMARCAL1 siRNA pool (20 nM, 48 h)
sgScramble lentivirus
sgSMARCAL1-1 stable KO
SMARCAL1
115 kDa
1.00
0.98
0.30
0.95
<0.02
GAPDH
37 kDa
1.0
0.5
0
0.30
<0.02
siRNA: ~30% residual (transient);
CRISPR: undetectable (complete KO)

figure s9

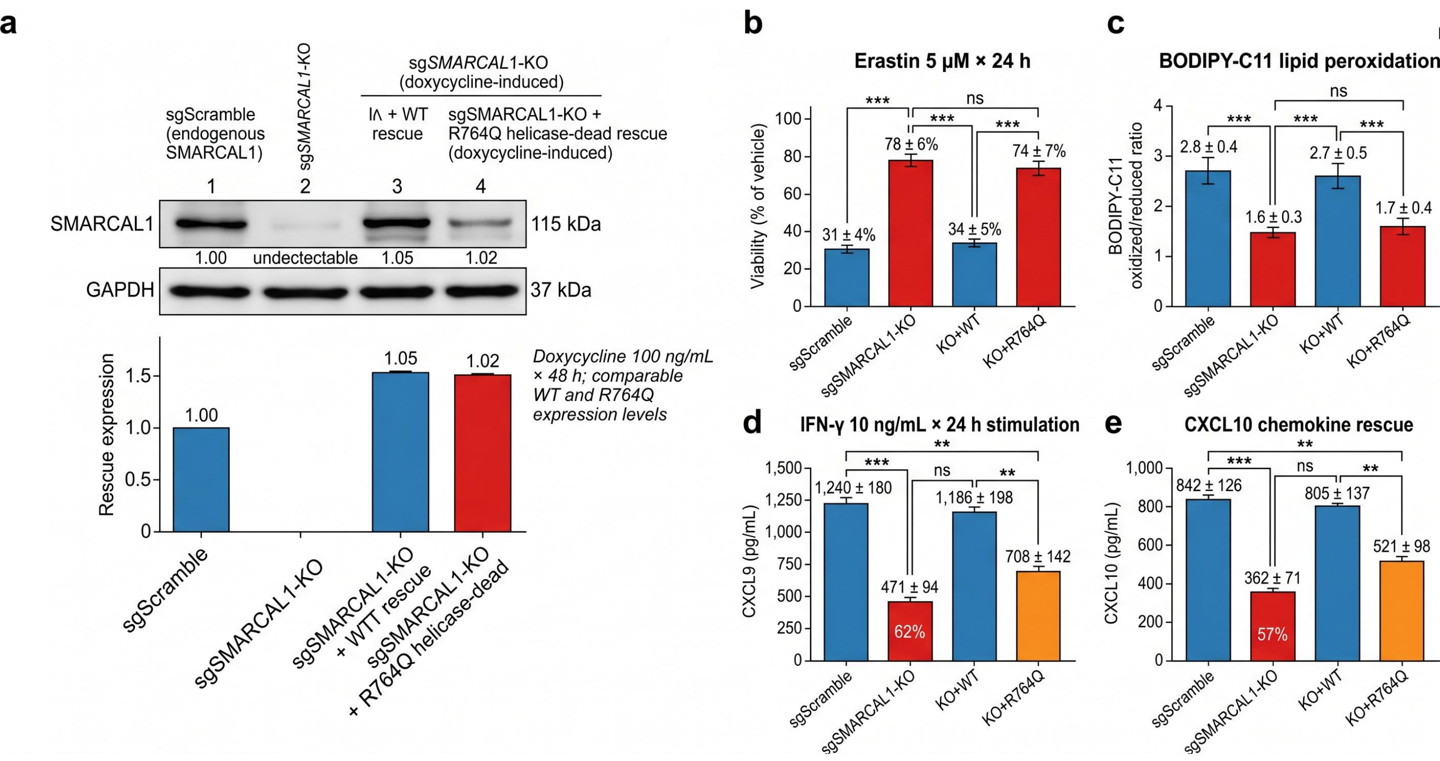
a
sgScramble (endogenous SMARCAL1)
sgSMARCAL1-KO
sgSMARCAL1-KO (doxycycline-induced)
I∧ + WT rescue
sgSMARCAL1-KO + R764Q helicase-dead rescue (doxycycline-induced)
1
2
3
4
SMARCAL1
115 kDa
1.00
undectectable
1.05
1.02
GAPDH
37 kDa
Rescue expression
Doxycycline 100 ng/mL × 48 h; comparable WT and R764Q expression levels
sgScramble
sgSMARCAL1-KO
sgSMARCAL1-KO + WT rescue
sgSMARCAL1-KO + R764Q helicase-dead
b
Erastin 5 μM × 24 h
Viability (% of vehicle)
31 ± 4%
78 ± 6%
34 ± 5%
74 ± 7%
c
BODIPY-C11 lipid peroxidation
BODIPY-C11 oxidized/reduced ratio
2.8 ± 0.4
1.6 ± 0.3
2.7 ± 0.5
1.7 ± 0.4
d
IFN-γ 10 ng/mL × 24 h stimulation
CXCL9 (pg/mL)
1,240 ± 180
471 ± 94
62%
1,186 ± 198
708 ± 142
e
CXCL10 chemokine rescue
CXCL10 (pg/mL)
842 ± 126
362 ± 71
57%
805 ± 137
521 ± 98
sgScramble
sgSMARCAL1-KO
KO+WT
KO+R764Q

figure s10

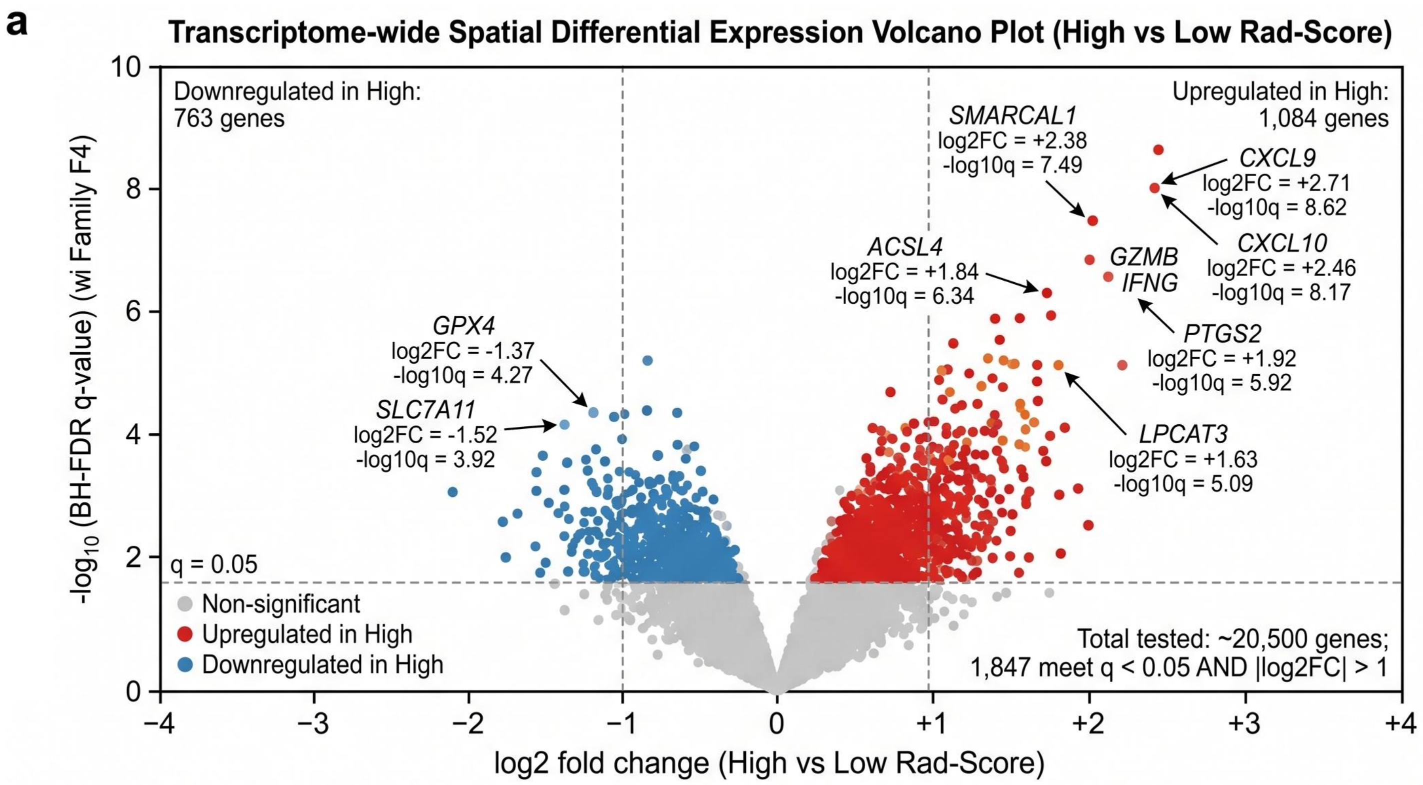

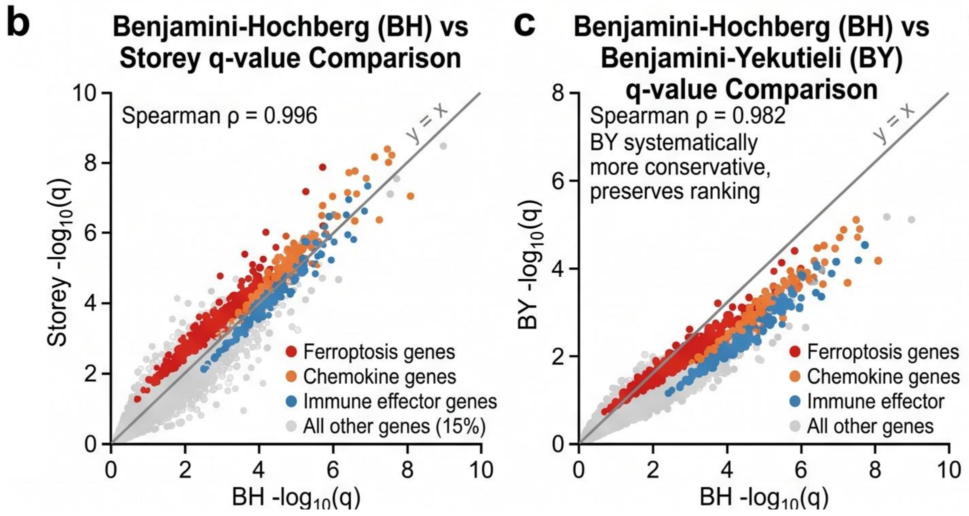

d **Summary of Significant Differential Expression by Multiple Testing Correction Method**

Method	Threshold	# significant	% of tested
BH	q < 0.05	1,847	8.9%
Storey	q < 0.05	2,104	10.2%
BY	q < 0.05	1,512	7.3%
BH conservative	q < 0.01	946	4.6%

All principal mechanistic findings remain significant under conservative BY correction

figure s11

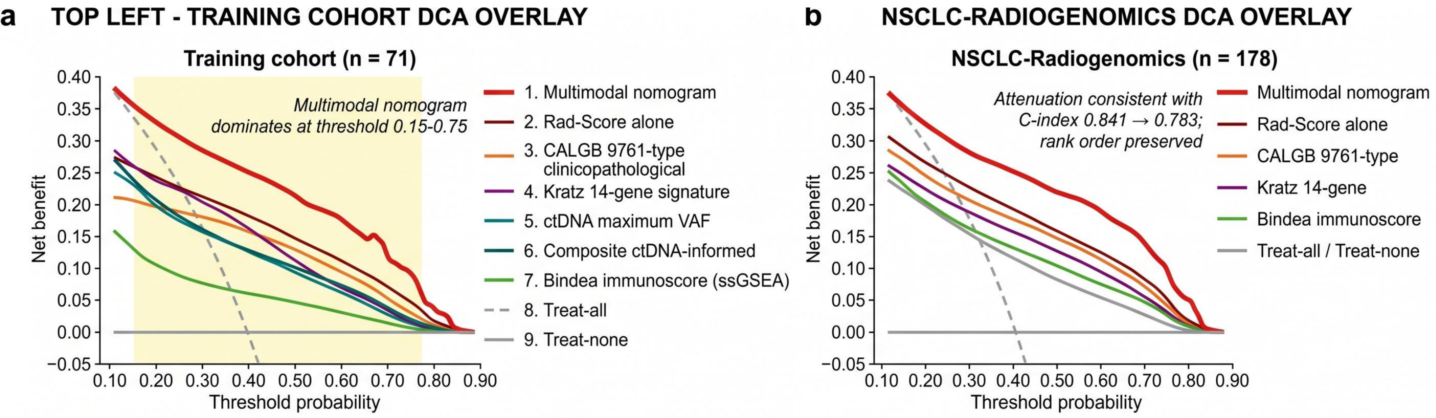

c **FOREST PLOT OF JOINT MODELS**

Joint model	Rad-Score adj. HR HR (95% CI)	*P*	Forest	Combined C-index
**REFERENCE:** Unadjusted Rad-Score	0.32 (0.18-0.58)	< 0.001		0.812
Joint with CALGB 9761-type	0.70 (0.48-1.02)	0.064		0.818
Joint with Clinical+TMB	0.66 (0.46-0.94)	0.022		0.841
Joint with Kratz 14-gene	0.74 (0.53-1.03)	0.075		0.824
Joint with ctDNA max VAF	0.72 (0.51-1.02)	0.065		0.834
Joint with ctDNA composite	0.68 (0.48-0.98)	0.039		0.847
Joint with Bindea	0.78 (0.56-1.09)	0.142		0.836

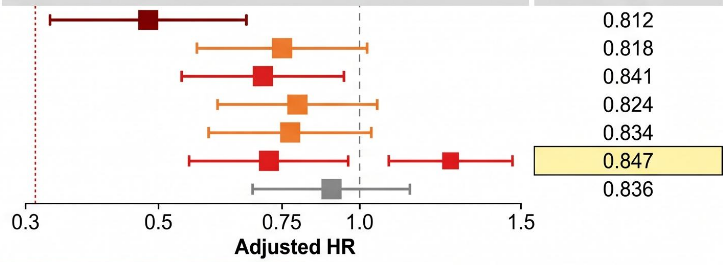

Supplement: Supplementary file 1 [file DataSheet1.pdf]
