# Supplementary material for: Non-invasive radiogenomic mapping of the SMARCAL1-driven ferroptotic niche is associated with longitudinal MRD-negative surveillance in early-stage NSCLC
Source: Front Immunol. 2026 May 25;17:1835413. doi: 10.3389/fimmu.2026.1835413 (PMC13243389; doi:10.3389/fimmu.2026.1835413)
Supplement: Supplementary file 2 [file DataSheet2.pdf]

Supplementary Tables S1–S9

Supplementary Table S1. **Stability-selected radiomic features (top 30 by Π ranking) and final 9-feature Rad-Score signature with LASSO coefficients and univariate Cox hazard ratios.**

#	Feature Name (PyRadiomics)	Class	ROI	Π	Univariate HR (95% CI)	P value	LASSO β (final)
**1**	*wavelet-LLH_glszm_ZoneEntropy*	**GLSZM**	**Core**	**0.94**	**0.41 (0.26–0.64)**	**< 0.001**	**−0.312**
**2**	*original_shape_SurfaceVolumeRatio*	**Shape**	**Core**	**0.89**	**1.68 (1.21–2.34)**	**0.002**	**+0.145**
**3**	*original_firstorder_90Percentile*	**First-order**	**Rim**	**0.88**	**0.58 (0.38–0.88)**	**0.010**	**−0.187**
**4**	*wavelet-HHL_glcm_Idmn*	**GLCM**	**Core**	**0.86**	**0.52 (0.34–0.79)**	**0.002**	**−0.218**
**5**	*wavelet-HLL_glrlm_ShortRunEmphasis*	**GLRLM**	**Rim**	**0.83**	**0.49 (0.31–0.77)**	**0.002**	**−0.256**
**6**	*wavelet-LLL_ngtdm_Contrast*	**NGTDM**	**Core**	**0.81**	**0.64 (0.43–0.96)**	**0.031**	**−0.173**
**7**	*wavelet-LHH_gldm_DependenceNonUniformity*	**GLDM**	**Rim**	**0.77**	**1.54 (1.09–2.17)**	**0.014**	**+0.201**
**8**	*log-sigma-3-mm-3D_glcm_Correlation*	**GLCM**	**Core**	**0.72**	**0.68 (0.46–1.00)**	**0.050**	**−0.094**
**9**	*log-sigma-5-mm-3D_firstorder_Skewness*	**First-order**	**Rim**	**0.68**	**0.72 (0.49–1.05)**	**0.088**	**−0.122**
**10**	*wavelet-HLH_glcm_ClusterShade*	GLCM	Core	0.62	1.34 (0.95–1.89)	0.094	—
**11**	*original_shape_Sphericity*	Shape	Core	0.61	0.74 (0.51–1.07)	0.109	—
**12**	*original_firstorder_Energy*	First-order	Core	0.58	1.28 (0.89–1.84)	0.182	—
**13**	*wavelet-LHL_glszm_GrayLevelNonUniformity*	GLSZM	Rim	0.56	1.22 (0.87–1.71)	0.253	—
**14**	*log-sigma-1-mm-3D_glrlm_RunLengthNonUniformity*	GLRLM	Core	0.54	1.19 (0.84–1.68)	0.327	—
**15**	*wavelet-HHH_glcm_Correlation*	GLCM	Rim	0.52	0.81 (0.57–1.15)	0.237	—
**16**	*original_firstorder_Kurtosis*	First-order	Core	0.49	1.15 (0.82–1.61)	0.427	—
**17**	*wavelet-LLH_glcm_Idmn*	GLCM	Rim	0.48	0.84 (0.59–1.19)	0.325	—
**18**	*original_shape_Elongation*	Shape	Core	0.46	1.11 (0.79–1.55)	0.541	—
**19**	*log-sigma-3-mm-3D_gldm_DependenceEntropy*	GLDM	Rim	0.44	0.87 (0.62–1.22)	0.420	—
**20**	*wavelet-HLL_firstorder_Entropy*	First-order	Core	0.42	1.09 (0.78–1.53)	0.609	—
**21**	*original_shape_LeastAxisLength*	Shape	Core	0.41	1.08 (0.77–1.51)	0.653	—
**22**	*wavelet-LHL_ngtdm_Busyness*	NGTDM	Rim	0.39	0.91 (0.64–1.29)	0.593	—
**23**	*log-sigma-5-mm-3D_glcm_Idmn*	GLCM	Core	0.38	0.92 (0.65–1.30)	0.637	—
**24**	*wavelet-LLL_gldm_LargeDependenceEmphasis*	GLDM	Rim	0.37	1.07 (0.76–1.50)	0.698	—
**25**	*original_firstorder_10Percentile*	First-order	Rim	0.36	1.06 (0.76–1.49)	0.725	—
**26**	*wavelet-HHH_glszm_ZoneVariance*	GLSZM	Core	0.34	0.95 (0.67–1.34)	0.770	—
**27**	*log-sigma-1-mm-3D_ngtdm_Complexity*	NGTDM	Core	0.32	1.04 (0.74–1.46)	0.822	—
**28**	*wavelet-HLH_glrlm_LongRunEmphasis*	GLRLM	Rim	0.31	0.97 (0.69–1.36)	0.855	—
**29**	*original_shape_Flatness*	Shape	Core	0.30	1.03 (0.73–1.45)	0.867	—
**30**	*wavelet-LHL_glcm_ClusterTendency*	GLCM	Rim	0.28	0.98 (0.70–1.38)	0.911	—

Feature nomenclature follows PyRadiomics v3.0.1 (RRID:SCR_017869) Image Biomarker Standardisation Initiative (IBSI)-compliant conventions. Π denotes the stability selection frequency (proportion of B = 500 bootstrap replicates in which each feature was retained at the tuned LASSO penalty). The final 9-feature Rad-Score signature comprises features 1–9 (highlighted in light green; $\Pi \geq 0.60$ stability threshold, seven with $\Pi \geq 0.80$ high-confidence threshold). The LASSO penalty λ was tuned by nested 5-fold cross-validation; the complete model coefficients are locked in Rad-Score_v1.0_locked_2026-02-15.

The continuous Rad-Score is computed as $\Sigma (\beta_i \times feature_i)$ for i = 1 to 9. The dichotomization cut-off at Rad-Score = +0.15 was determined by maximally selected rank statistics (survminer R package), stratifying the 71-patient cohort into High (n = 35) and Low (n = 36) Rad-Score tiers; sensitivity analyses using the continuous Rad-Score are reported throughout the manuscript.

Abbreviations: β, LASSO regression coefficient; CI, confidence interval; GLCM, gray-level co-occurrence matrix; GLDM, gray-level dependence matrix; GLRLM, gray-level run length matrix; GLSZM, gray-level size zone matrix; HHH/HHL/HLH/HLL/LHH/LHL/LLH/LLL, 3D wavelet decomposition sub-bands; HR, hazard ratio; Idmn, inverse difference moment normalized; LASSO, least absolute shrinkage and selection operator; log-sigma, Laplacian-of-Gaussian filter sigma in mm; NGTDM, neighborhood gray-tone difference matrix; Π, stability selection frequency; ROI, region of interest.

Supplementary Table S2. **Characteristics of the two external locked-model validation cohorts and direct comparison with the training cohort.**

Characteristic	Training (CTONG 2201-nested)	NSCLC-Radiogenomics	Lung3
Cohort Provenance and Inclusion			
**Cohort origin**	Guangxi Medical University First Affiliated Hospital, China (single-center, prospectively accrued)	TCIA NSCLC-Radiogenomics (Bakr et al. Scientific Data 2018); Stanford / Palo Alto VA, United States	TCIA Lung3 (Aerts et al. Nat Commun 2014); MAASTRO Clinic, Maastricht, Netherlands
**Data access identifier**	IRB # GXYDY-2022-0412 (institutional)	TCIA DOI: 10.7937/K9/TCIA.2017.7hs46erv	TCIA Dataset ID: Lung3; DOI: 10.7937/K9/TCIA.2014.4jvytv0i
**Patients after QC filtering, n**	71 (from 96 screened)	178 (from 211 available)	89 (from 115 available)
**Inclusion criteria applied**	R0 resection, stage IB–IIIA, no neoadjuvant, MRD-negative at 2 timepoints, no adjuvant therapy, CT quality adequate	R0 resection, stage IB–IIIA, no neoadjuvant, CT quality adequate, DFS follow-up and expression data available	R0 resection, stage IB–IIIA, CT quality adequate, OS follow-up available
**Recruitment period**	2022–2024	2008–2012	2007–2011
Demographics and Clinical Characteristics			
**Patients, n**	71 (High 35 + Low 36)	178	89
**Age, years, median (IQR)**	62 (55–68)	67 (60–74)	66 (59–72)
**Male sex, n (%)**	42 (59.2%)	112 (62.9%)	58 (65.2%)
**Ethnicity**	Han Chinese (100%)	White 88%, Black 7%, Asian 3%, Other 2%	White European (predominantly Dutch)
**Ever-smoker, n (%)**	39 (54.9%)	148 (83.1%)	82 (92.1%)
**Histologic subtype**	LUAD 74.6%, LUSC 25.4%	LUAD 67.4%, LUSC 27.5%, Other 5.1%	LUAD 47.2%, LUSC 42.7%, Other 10.1%
**Stage IB, n (%)**	26 (36.6%)	71 (39.9%)	31 (34.8%)
**Stage II, n (%)**	29 (40.8%)	62 (34.8%)	26 (29.2%)
**Stage IIIA, n (%)**	16 (22.5%)	45 (25.3%)	32 (36.0%)
**EGFR mutation, n (%)**	24 (33.8%)	18 (10.1%)	6 (6.7%)
**KRAS mutation, n (%)**	11 (15.5%)	52 (29.2%)	27 (30.3%)
CT Imaging and Feature Extraction			
**Scanner manufacturers**	Siemens 39.4%, GE 36.6%, Philips 24.0%	GE 56.2%, Siemens 28.7%, Philips 15.1%	Siemens 100% (Somatom Sensation Open)
**Slice thickness, mm**	1.0–1.25 (target 1.0)	1.0–3.0 (variable)	3.0 (uniform)
**Tube voltage, kVp**	120 (standardized)	120 (97%), 140 (3%)	120 (100%)
**Reconstruction kernel harmonization**	ComBat applied (scanner as batch; MRD-free survival preserved)	ComBat re-applied using training cohort parameters	ComBat re-applied using training cohort parameters
**Radiomic feature extraction software**	PyRadiomics v3.0.1 (IBSI-compliant)	Same version + same settings	Same version + same settings
**ROI segmentation approach**	3D Slicer v5.0.3, manual by 2 radiologists, ICC ≥ 0.80	Same 3D Slicer protocol by 1 independent radiologist; inter-rater ICC ≥ 0.82 on 20% subset	Same 3D Slicer protocol by 1 independent radiologist; inter-rater ICC ≥ 0.85 on full cohort
Molecular / Genomic Data			

**Expression platform**	Illumina RNA-seq (TruSeq Stranded)	Affymetrix U133 Plus 2.0 microarray (127 of 178)	Affymetrix U133 Plus 2.0 microarray
**WES / TMB availability**	Yes (n = 71; SureSelect XT V6)	Imputed from RNA-seq TMB surrogate for n = 127	Not available (TMB imputation unreliable)
**MRD ctDNA monitoring**	Yes (n = 71; post-op months 1 and 6)	Not performed	Not performed
**Multimodal nomogram feasibility**	Fully feasible (all inputs available)	Fully feasible with imputed TMB (n = 127); Rad-Score alone for remaining (n = 51)	Rad-Score alone (no TMB available)
Survival Endpoint and Follow-up			
**Primary endpoint**	MFS (MRD-free survival from post-op month 1 until MRD conversion)	DFS (disease-free survival; radiographic recurrence)	OS (overall survival)
**Secondary endpoint**	DFS, OS	OS (when available)	—
**Median follow-up, months**	24.5 (IQR 18.3–31.2)	49.2 (IQR 32.8–64.4)	37.8 (IQR 24.1–55.6)
**Events at endpoint, n**	26 MRD conversions; 17 recurrences	61 DFS events; 43 OS events	34 OS events
Locked-Model External Validation Performance			
**Rad-Score_v1.0_locked timestamp**	2026-02-15 (reference)	Applied 2026-02-22	Applied 2026-02-25
**C-index (95% CI)**	0.841 apparent / 0.798 optimism-corrected	0.783 (0.721–0.845)	0.741 (0.651–0.831)
**Hazard ratio High vs Low Rad-Score (95% CI)**	0.32 (0.18–0.58)	0.46 (0.28–0.76)	0.52 (0.29–0.93)
**Log-rank P**	< 0.001	0.002	0.027
**24-month event-free survival, High / Low (%)**	82 / 38	69 / 48	71 / 54
**Calibration slope (external)**	1.00 (reference)	0.87 (0.72–1.02)	0.79 (0.62–0.96)

The locked-model protocol froze all model components (9 radiomic features, LASSO coefficients, ComBat harmonization parameters, dichotomization cut-off +0.15, uniform shrinkage factor γ = 0.84) before any external data access. The model artifact was version-controlled (Rad-Score_v1.0_locked_2026-02-15) and deposited to FigShare with DOI assignment upon acceptance.

The progressive C-index attenuation from training apparent (0.841) → optimism-corrected (0.798) → external NSCLC-RG (0.783) → external Lung3 (0.741) is consistent with published ranges for similar p/n ratios in radiogenomic models (Hosny et al. PLOS Medicine 2018) and reflects expected distribution shift across scanner platforms, ethnic composition, and endpoint types.

Abbreviations: ComBat, combatting batch effects method; C-index, concordance index; CI, confidence interval; CTONG, Chinese Thoracic Oncology Group; DFS, disease-free survival; EGFR, epidermal growth factor receptor; IBSI, Image Biomarker Standardisation Initiative; ICC, intra-class correlation coefficient; IQR, interquartile range; IRB, institutional review board; KRAS, Kirsten rat sarcoma viral oncogene homolog; kVp, kilovolt peak; LUAD, lung adenocarcinoma; LUSC, lung squamous cell carcinoma; MFS, MRD-free survival; MRD, molecular residual disease; NSCLC-RG, NSCLC-Radiogenomics cohort; OS, overall survival; QC, quality control; ROI, region of interest; TCIA, The Cancer Imaging Archive; TMB, tumor mutational burden; WES, whole-exome sequencing.

Supplementary Table S3. Complete antibody, reagent, and resource list for multiplex immunofluorescence, immunohistochemistry, Western blot, flow cytometry, and functional CRISPR experiments.

Target / Reagent	Vendor	Catalog #	Clone	Host / Type	Dilution	Application	RRID
Primary Antibodies — Immunohistochemistry and Multiplex Immunofluorescence							
**SMARCAL1**	Abcam	ab154804	EPR12536	Rabbit mAb	1:200 (IHC) / 1:100 (mIF)	IHC, mIF, WB	AB_2783732
**ACSL4**	Santa Cruz	sc-365230	F-4	Mouse mAb	1:100 (IHC) / 1:50 (mIF)	IHC, mIF, WB	AB_10843105
**GPX4**	Abcam	ab125066	EPNCIR144	Rabbit mAb	1:150 (IHC) / 1:100 (mIF)	IHC, mIF, WB	AB_10973901
**4-HNE (ferroptosis marker)**	Abcam	ab46545	Polyclonal	Rabbit pAb	1:200 (IHC) / 1:100 (mIF)	IHC, mIF	AB_722490
**CD8α**	Dako	M7103	C8/144B	Mouse mAb	1:100 (IHC)	IHC, mIF	AB_2075537
**CD8 (for mIF)**	Abcam	ab237709	SP16	Rabbit mAb	1:100 (mIF)	mIF	AB_2860024
**CXCR3**	Abcam	ab288574	EPR23463-26	Rabbit mAb	1:50 (mIF)	mIF, flow	AB_2935731
**CXCL9 (MIG)**	R&D Systems	MAB392	49106	Mouse mAb	1:50 (mIF) / 10 μg/mL neut.	mIF, neut.	AB_354237
**CXCL10 (IP-10)**	R&D Systems	MAB266	33036	Mouse mAb	1:50 (mIF) / 10 μg/mL neut.	mIF, neut.	AB_2292487
**FoxP3**	eBioscience	14-4776-82	236A/E7	Mouse mAb	1:100 (mIF)	mIF	AB_467555
**CD4**	Abcam	ab133616	EPR6855	Rabbit mAb	1:200 (mIF)	mIF	AB_2750883
**Pan-Cytokeratin**	Agilent/Dako	M3515	AE1/AE3	Mouse mAb	1:200 (mIF)	mIF (tumor marker)	AB_2132885
**CD68**	Dako	M0814	KP1	Mouse mAb	1:100 (mIF)	mIF (macrophages)	AB_2314148
Primary Antibodies — Western Blot (signaling pathway)							
**phospho-STING (Ser366)**	Cell Signaling	72971	E9A9K	Rabbit mAb	1:1,000	WB	AB_2799831
**Total STING**	Cell Signaling	13647	D2P2F	Rabbit mAb	1:1,000	WB	AB_2732796
**phospho-TBK1 (Ser172)**	Cell Signaling	5483	D52C2	Rabbit mAb	1:1,000	WB	AB_10693472
**Total TBK1**	Cell Signaling	3013	D1B4	Rabbit mAb	1:1,000	WB	AB_2199749
**phospho-IRF3 (Ser396)**	Cell Signaling	29047	D6O1M	Rabbit mAb	1:1,000	WB	AB_2773013
**Total IRF3**	Cell Signaling	11904	D83B9	Rabbit mAb	1:1,000	WB	AB_2722521
**GAPDH (loading control)**	Cell Signaling	2118	14C10	Rabbit mAb	1:5,000	WB	AB_561053
Secondary Antibodies and Detection Reagents							
**Anti-rabbit HRP (polymer)**	Agilent/Dako	K4003	—	Goat anti-Rb	Ready-to-use	IHC	AB_2630375
**Anti-mouse HRP (polymer)**	Agilent/Dako	K4001	—	Goat anti-Ms	Ready-to-use	IHC	AB_2687452
**Opal 520 Fluorophore**	Akoya Biosciences	FP1487001KT	—	—	1:150	mIF	AB_2936023

**Opal 570 Fluorophore**	Akoya Biosciences	FP1488001KT	—	—	1:150	mIF	AB_2936024
**Opal 620 Fluorophore**	Akoya Biosciences	FP1495001KT	—	—	1:150	mIF	AB_2936025
**Opal 650 Fluorophore**	Akoya Biosciences	FP1496001KT	—	—	1:150	mIF	AB_2936026
**Opal 690 Fluorophore**	Akoya Biosciences	FP1497001KT	—	—	1:150	mIF	AB_2936027
**Spectral DAPI**	Akoya Biosciences	FP1490	—	—	1:1,000	mIF	AB_2936028
Flow Cytometry Antibodies and Dyes							
**CD8-APC**	BioLegend	344722	SK1	Mouse anti-Hu	5 µL/10^6 cells	Flow	AB_2075392
**CXCR3-PE**	BioLegend	353706	G025H7	Mouse anti-Hu	5 µL/10^6 cells	Flow	AB_10983070
**CD45-PerCP-Cy5.5**	BioLegend	304028	HI30	Mouse anti-Hu	5 µL/10^6 cells	Flow	AB_893338
**BODIPY 581/591 C11 dye**	Invitrogen/Thermo	D3861	—	—	10 µM, 30 min	Flow (lipid ROS)	—
**FerroOrange dye**	Dojindo	F374	—	—	1 µM, 30 min	Flow (Fe^{2+})	—
**CellTrace Violet**	Invitrogen	C34557	—	—	5 µM, 20 min	Flow	—
**7-AAD viability dye**	BioLegend	420404	—	—	5 µL/test	Flow	AB_10643380
ELISA Kits and Functional Assay Reagents							
**Human CXCL9 ELISA**	R&D Systems	DCX900	—	Quantikine	—	ELISA	—
**Human CXCL10 ELISA**	R&D Systems	DIP100	—	Quantikine	—	ELISA	—
**Human IFN-γ ELISA**	R&D Systems	DIF50C	—	Quantikine	—	ELISA	—
**Human Granzyme B ELISA**	R&D Systems	DGZB00	—	Quantikine	—	ELISA	—
**CellTiter-Glo 2.0 Assay**	Promega	G9241	—	—	1:1 ratio	Viability	—
**TBARS assay (MDA)**	Cayman Chemical	10009055	—	—	Per protocol	MDA (ferroptosis)	—
**GSH/GSSG-Glo Assay**	Promega	V6611	—	—	Per protocol	GSH/GSSG	—
Cell Lines, Chemical Compounds, and CRISPR Reagents							
**A549 (NSCLC adenocarcinoma)**	ATCC	CCL-185	—	—	—	Cell line	CVCL_0023
**H1299 (NSCLC, TP53-null)**	ATCC	CRL-5803	—	—	—	Cell line	CVCL_0060
**erastin (class I ferroptosis inducer)**	Selleckchem	S7242	—	—	5 µM, 24 h	Ferroptosis induction	—
**RSL3 (class II, GPX4 inhibitor)**	Selleckchem	S8155	—	—	1 µM, 12 h	Ferroptosis induction	—
**Liproxstatin-1 (ferroptosis inhibitor)**	Selleckchem	S7699	—	—	1 µM	Ferroptosis inhibition	—
**AMG 487 (CXCR3 antagonist)**	Tocris	4883	—	—	1 µM	CXCR3 blockade	—
**diABZI (STING agonist)**	Selleckchem	S8796	—	—	10 µM, 6 h	STING activation	—
**Dynabeads Human anti-CD3/CD28**	Gibco	11131D	—	—	1:1 ratio	T cell activation	—
**Recombinant human IL-2**	PeproTech	200-02	—	—	50 U/mL	T cell expansion	—

**Recombinant human IFN-γ**	R&D Systems	285-IF-100	—	—	10 ng/mL	Stimulation	—
**lentiCRISPR v2 plasmid**	Addgene	52961	—	—	—	CRISPR backbone	Addgene_52961
**psPAX2 (packaging)**	Addgene	12260	—	—	—	Lentivirus production	Addgene_12260
**pMD2.G (envelope)**	Addgene	12259	—	—	—	Lentivirus production	Addgene_12259
**sgSMARCAL1-1**	Synthego	Custom	—	—	—	Knockout (exon 3)	—
**sgSMARCAL1-2**	Synthego	Custom	—	—	—	Knockout (exon 3)	—
**sgScramble (non-targeting control)**	Synthego	Custom	—	—	—	Non-target control	—
Spatial Transcriptomics and Molecular Biology Reagents							
**Visium Spatial 3' Gene Expression**	10x Genomics	1000187	—	—	—	Spatial RNA-seq	—
**Chromium Next GEM v3.1 (for PBMC)**	10x Genomics	1000269	—	—	—	scRNA-seq	—
**KAPA HyperPrep WES**	Roche/KAPA	KK8504	—	—	—	WES library prep	—
**SureSelectXT Human All Exon V6**	Agilent	5190-8864	—	—	—	Exome capture	—
**TruSeq Stranded mRNA**	Illumina	20020594	—	—	—	RNA-seq library	—

All RRIDs were verified against the SciCrunch Resource Identification Portal (https://scicrunch.org/resources) at the time of submission. Cell line authentication by STR profiling and routine Mycoplasma testing (MycoAlert PLUS, Lonza LT07-710) were performed within 6 months of all experiments. Human PBMC from healthy donors were obtained under institutional IRB approval (protocol #IRB-2025-0471); written informed consent was obtained from all participants.

Abbreviations: Hu, human; mAb, monoclonal antibody; mIF, multiplex immunofluorescence; Ms, mouse; neut., neutralizing application; NT, non-targeting; pAb, polyclonal antibody; Rb, rabbit; RRID, Research Resource Identifier; WB, Western blot; WES, whole-exome sequencing.

Supplementary Table S4. **Complete baseline clinical, pathological, and molecular characteristics of the CTONG 2201-nested training cohort (N = 71), stratified by Rad-Score tier.**

Characteristic	Overall (N = 71)	High Rad-Score (n = 35)	Low Rad-Score (n = 36)	P value*
Demographics				
Age, years, median (IQR)	62 (55–68)	61 (54–67)	63 (56–69)	0.412
Age ≥ 65, n (%)	28 (39.4%)	13 (37.1%)	15 (41.7%)	0.810
Sex				0.810
Male, n (%)	42 (59.2%)	20 (57.1%)	22 (61.1%)	
Female, n (%)	29 (40.8%)	15 (42.9%)	14 (38.9%)	
BMI, kg/m², mean (SD)	23.4 (2.8)	23.0 (2.6)	23.8 (3.0)	0.241
Ethnicity (Han Chinese), n (%)	71 (100%)	35 (100%)	36 (100%)	1.000
Performance Status and Comorbidities				
ECOG Performance Status				0.609
0, n (%)	48 (67.6%)	25 (71.4%)	23 (63.9%)	
1, n (%)	23 (32.4%)	10 (28.6%)	13 (36.1%)	
Charlson Comorbidity Index ≥ 1, n (%)	19 (26.8%)	9 (25.7%)	10 (27.8%)	1.000
Smoking History				
Ever-smoker, n (%)	39 (54.9%)	18 (51.4%)	21 (58.3%)	0.637
Pack-years, median (IQR)†	24 (12–38)	22 (12–36)	27 (14–40)	0.391
Tumor Histopathology				
Histologic subtype				1.000
Adenocarcinoma (LUAD), n (%)	53 (74.6%)	26 (74.3%)	27 (75.0%)	
Squamous cell carcinoma (LUSC), n (%)	18 (25.4%)	9 (25.7%)	9 (25.0%)	
Pathological stage (AJCC 8th)				0.787
IB, n (%)	26 (36.6%)	14 (40.0%)	12 (33.3%)	
II (IIA + IIB), n (%)	29 (40.8%)	13 (37.1%)	16 (44.4%)	
IIIA, n (%)	16 (22.5%)	8 (22.9%)	8 (22.2%)	
Tumor size, cm, median (IQR)	3.2 (2.4–4.1)	3.0 (2.3–3.9)	3.4 (2.5–4.3)	0.276
Lymph node involvement (N+), n (%)	34 (47.9%)	16 (45.7%)	18 (50.0%)	0.815
Visceral pleural invasion, n (%)	22 (31.0%)	9 (25.7%)	13 (36.1%)	0.437
Lymphovascular invasion, n (%)	18 (25.4%)	7 (20.0%)	11 (30.6%)	0.417
Surgical and Treatment Details				
Resection type				1.000
Lobectomy, n (%)	62 (87.3%)	31 (88.6%)	31 (86.1%)	
Segmentectomy / wedge, n (%)	9 (12.7%)	4 (11.4%)	5 (13.9%)	

**R0 resection, n (%)**	71 (100%)	35 (100%)	36 (100%)	1.000
**Lymph nodes examined, median (IQR)**	14 (11–18)	14 (11–18)	14 (12–19)	0.708
**Adjuvant therapy received, n (%)**	0 (0%)	0 (0%)	0 (0%)	1.000
Molecular Profile				
**EGFR mutation, n (%)**	24 (33.8%)	13 (37.1%)	11 (30.6%)	0.622
**KRAS mutation, n (%)**	11 (15.5%)	5 (14.3%)	6 (16.7%)	1.000
**ALK rearrangement, n (%)**	4 (5.6%)	2 (5.7%)	2 (5.6%)	1.000
**TMB High (≥ 10 mut/Mb), n (%)**	19 (26.8%)	13 (37.1%)	6 (16.7%)	0.064
**APOBEC signature dominant, n (%)**	21 (29.6%)	14 (40.0%)	7 (19.4%)	0.072
**PD-L1 TPS ≥ 1%, n (%)**	41 (57.7%)	23 (65.7%)	18 (50.0%)	0.232
MRD Status and Clinical Outcomes				
**Baseline MRD-negative (post-op month 1), n (%)**	71 (100%)	35 (100%)	36 (100%)	1.000
**MRD-negative at month 6, n (%)**	71 (100%)	35 (100%)	36 (100%)	1.000
**Follow-up (months), median (IQR)**	24.5 (18.3–31.2)	26.1 (20.4–33.8)	22.9 (16.5–29.8)	0.088
**MRD conversion events, n (%)**	26 (36.6%)	7 (20.0%)	19 (52.8%)	0.006
**Radiographic recurrence, n (%)**	17 (23.9%)	4 (11.4%)	13 (36.1%)	0.025
**24-month MRD-free survival (%)**	62.0	82.0	38.0	< 0.001‡

**Wilcoxon rank-sum test for continuous variables (median, IQR); Fisher exact test or chi-square test for categorical variables (two-sided).*

†Pack-years calculated for ever-smokers only.

‡Log-rank test for 24-month MRD-free survival comparison; hazard ratio = 0.32 (95% CI 0.18–0.58).

Abbreviations: AJCC, American Joint Committee on Cancer; ALK, anaplastic lymphoma kinase; APOBEC, apolipoprotein B mRNA-editing enzyme catalytic polypeptide; BMI, body mass index; ECOG, Eastern Cooperative Oncology Group; EGFR, epidermal growth factor receptor; IQR, interquartile range; KRAS, Kirsten rat sarcoma viral oncogene homolog; LUAD, lung adenocarcinoma; LUSC, lung squamous cell carcinoma; MRD, molecular residual disease; PD-L1, programmed death-ligand 1; SD, standard deviation; TMB, tumor mutational burden; TPS, tumor proportion score.

Supplementary Table S5. Multi-omics summary comparison between High and Low Rad-Score tumors in the training cohort (N = 71).

Domain / Feature	High Rad-Score (n = 35)	Low Rad-Score (n = 36)	Fold / Δ	Raw P value*	BH-FDR q†
CIBERSORTx Immune Deconvolution (Family F1; 22 cell types tested)					
**$CD8^+$ T cells (% infiltration)**	24.5 ± 6.8	12.1 ± 4.3	**+12.4 pp**	4.3×10^{-6}	6.4×10^{-5}
**Activated memory $CD4^+$ T cells (%)**	8.2 ± 2.4	4.1 ± 1.7	**+4.1 pp**	2.1×10^{-5}	2.3×10^{-4}
**M1 macrophages (%)**	9.8 ± 3.1	5.4 ± 2.2	**+4.4 pp**	8.7×10^{-5}	6.4×10^{-4}
**M2 macrophages (%)**	12.3 ± 4.2	18.6 ± 5.8	**−6.3 pp**	3.2×10^{-4}	1.9×10^{-3}
**Regulatory T cells (%)**	1.4 ± 0.5	2.3 ± 0.9	**−0.9 pp**	2.8×10^{-3}	1.2×10^{-2}
**Activated NK cells (%)**	4.6 ± 1.8	3.2 ± 1.3	**+1.4 pp**	4.1×10^{-3}	1.5×10^{-2}
**Activated dendritic cells (%)**	3.8 ± 1.2	2.1 ± 0.8	**+1.7 pp**	1.1×10^{-4}	7.3×10^{-4}
**Naive B cells (%)**	2.9 ± 0.9	3.8 ± 1.1	**−0.9 pp**	0.048	0.106
**Neutrophils (%)**	3.4 ± 1.1	4.8 ± 1.6	**−1.4 pp**	0.017	0.047
**Mast cells (activated, %)**	1.2 ± 0.4	1.6 ± 0.5	**−0.4 pp**	0.084	0.154
COSMIC Mutational Signatures v3.2 (Family F2; top contributions)					
**SBS2 (APOBEC deamination)**	0.184 ± 0.058	0.072 ± 0.034	**+2.6×**	2.8×10^{-5}	2.2×10^{-4}
**SBS13 (APOBEC/REV1)**	0.147 ± 0.051	0.061 ± 0.028	**+2.4×**	1.4×10^{-4}	7.3×10^{-4}
**SBS4 (tobacco smoking)**	0.198 ± 0.074	0.216 ± 0.082	**−0.9×**	0.387	0.503
**SBS3 (HR deficiency)**	0.042 ± 0.021	0.068 ± 0.034	**−1.6×**	0.012	0.039
**SBS6 (MMR deficiency)**	0.029 ± 0.013	0.033 ± 0.016	**−1.1×**	0.417	0.514
**SBS5 (clock-like, aging)**	0.121 ± 0.042	0.138 ± 0.048	**−1.1×**	0.221	0.338
Ferroptosis Pathway Gene Modules (Family F3; GSVA scores)					
**Overall Ferroptosis Activity Score (FerrDb v2)**	0.742 ± 0.187	0.281 ± 0.142	**+2.6×**	1.8×10^{-7}	4.2×10^{-6}
**Lipid peroxidation module (12 genes)**	0.681 ± 0.162	0.318 ± 0.131	**+2.1×**	7.3×10^{-6}	4.6×10^{-5}
**Iron metabolism module (8 genes)**	0.534 ± 0.148	0.362 ± 0.127	**+1.5×**	3.8×10^{-4}	1.7×10^{-3}
**Anti-oxidant defense module (GPX4, SLC7A11, etc.)**	−0.412 ± 0.143	0.187 ± 0.109	**Inverted**	8.3×10^{-6}	5.2×10^{-5}
KEGG Pathway Enrichment (Family F4b; top 10 of 186 pathways)					
**Cytokine-cytokine receptor interaction (hsa04060)**	NES +2.81	NES −0.42	—	3.1×10^{-6}	1.2×10^{-4}
**Chemokine signaling pathway (hsa04062)**	NES +2.74	NES −0.38	—	5.2×10^{-6}	1.9×10^{-4}
**Antigen processing and presentation (hsa04612)**	NES +2.48	NES −0.17	—	1.8×10^{-5}	4.1×10^{-4}
**Natural killer cell-mediated cytotoxicity (hsa04650)**	NES +2.34	NES −0.21	—	2.7×10^{-5}	5.2×10^{-4}
**T cell receptor signaling pathway (hsa04660)**	NES +2.19	NES −0.09	—	6.4×10^{-5}	1.1×10^{-3}
**Ferroptosis (hsa04216)**	NES +2.08	NES −1.14	—	1.2×10^{-4}	1.8×10^{-3}
**Cellular senescence (hsa04218)**	NES +1.82	NES +0.24	—	1.8×10^{-3}	1.4×10^{-2}
**Glutathione metabolism (hsa00480)**	NES −1.67	NES +1.12	—	3.2×10^{-3}	2.1×10^{-2}
**PI3K-AKT signaling pathway (hsa04151)**	NES −1.42	NES +0.88	—	0.012	0.054
**Wnt signaling pathway (hsa04310)**	NES −1.28	NES +0.63	—	0.028	0.091
Composite Immune and Biological Indices					
**Bindea 28-cell-type immunoscore (ssGSEA)**	+0.624 ± 0.172	−0.418 ± 0.198	—	1.4×10^{-6}	1.1×10^{-5}

**Immune Effector Score (CD8⁺PRF1⁺GZMB⁺)**	+0.712 ± 0.213	−0.384 ± 0.176	—	2.7×10^{-7}	5.4×10^{-6}
**Interferon-γ response hallmark (Hallmark, MSigDB)**	+0.568 ± 0.187	−0.321 ± 0.162	—	8.2×10^{-6}	4.3×10^{-5}
**TGF-β signaling hallmark**	−0.248 ± 0.113	+0.184 ± 0.098	—	1.4×10^{-4}	7.2×10^{-4}
**Epithelial-mesenchymal transition hallmark**	−0.184 ± 0.102	+0.156 ± 0.094	—	1.2×10^{-3}	0.024

**Wilcoxon rank-sum test (two-sided) comparing continuous values between High Rad-Score (n = 35) and Low Rad-Score (n = 36) patient groups.*

†Benjamini-Hochberg FDR correction within the pre-specified testing family indicated in each section header. Test counts by family: F1 = 22 CIBERSORTx cell types; F2 = 78 SBS signatures; F3 = 382 FerrDb v2 genes; F4b = 186 KEGG pathways. See Supplementary Table S5 for the complete hierarchical multiple-testing framework.

Continuous Spearman correlations with Rad-Score (not shown): ρ = 0.87 (Ferroptosis Activity Score); ρ = 0.79 (SMARCAL1 expression); ρ = 0.82 ($CD8^+$ T cell infiltration); all P < 0.001.

Abbreviations: APOBEC, apolipoprotein B mRNA-editing enzyme catalytic polypeptide; BH-FDR, Benjamini–Hochberg false discovery rate; CIBERSORTx, Cell-type Identification By Estimating Relative Subsets Of RNA Transcripts; COSMIC, Catalogue of Somatic Mutations in Cancer; FerrDb, ferroptosis database; GSVA, gene set variation analysis; HR, homologous recombination; KEGG, Kyoto Encyclopedia of Genes and Genomes; MMR, mismatch repair; MSigDB, Molecular Signatures Database; NES, normalized enrichment score; NK, natural killer; pp, percentage points; SBS, single-base substitution; ssGSEA, single-sample gene set enrichment analysis; TGF-β, transforming growth factor beta.

Supplementary Table S6. **Pre-specified nine-family hierarchical multiple-testing correction framework with Bonferroni-Holm cross-family significance thresholds.**

Family	Biological Domain / Test Ensemble	# Tests	Within-family Correction	Observed Minimum Raw P	Adjusted q	Cross-family Holm P
**F1**	CIBERSORTx immune-cell deconvolution (22 cell-type fractions)	22	BH-FDR ($q < 0.05$)	4.3×10^{-6} ($CD8^{+}$)	6.4×10^{-5}	—
**F2**	COSMIC single-base substitution signatures (v3.2)	78	BH-FDR ($q < 0.05$)	2.8×10^{-5} (SBS2)	2.2×10^{-4}	—
**F3**	Ferroptosis gene differential expression (FerrDb v2)	382	BH-FDR ($q < 0.05$)	1.8×10^{-7} (FASN)	4.2×10^{-6}	—
**F4**	Transcriptome-wide spatial DE (parent family)	20,500	BH-FDR + BY sensitivity	2.4×10^{-9} (CXCL9)	8.6×10^{-8}	—
**F4b**	KEGG pathway enrichment (nested in F4)	186	BH-FDR + hypergeometric	3.1×10^{-6} (hsa04060)	1.2×10^{-4}	—
**F5**	Spatial ligand-receptor interactions (CellChatV2)	3,024	BH-FDR ($q < 0.01$ stringent)	8.7×10^{-9} (CXCL9-CXCR3)	1.8×10^{-6}	—
**F6**	Spatial cell2location deconvolution (28 cell types)	28	BH-FDR ($q < 0.05$)	3.6×10^{-6} ($CXCR3^{+}$ CD8)	3.8×10^{-5}	—
**F7**	Clinicopathological associations with Rad-Score	12	BH-FDR ($q < 0.05$)	6.0×10^{-3} (MRD conversion)	0.024	—
**F8**	Multi-omics composite correlations (9 variables)	15	BH-FDR + BY sensitivity	1.8×10^{-7} (Ferroptosis Score)	1.4×10^{-6}	—
**F9**	**Cross-family conjunctive primary mechanistic axes**	4	Bonferroni-Holm (FWER < 0.05)	Family-specific minima	(see below)	**All $< 2.5 \times 10^{-4}$**

Family F9 Bonferroni-Holm-Adjusted P-Values for the Four Primary Conjunctive Mechanistic Axes

Rank	Primary Mechanistic Axis	Family Min Raw P	Bonferroni-Holm Multiplier	Monotonicity-Enforced Adjusted P	Conclusion
**1**	Ferroptosis gene family (F3)	2.8×10^{-6}	$\times 4 = 1.12 \times 10^{-5}$	$\mathbf{1.12 \times 10^{-5}}$	**Significant**
**2**	Immune deconvolution family (F1)	3.1×10^{-6}	$\times 3 = 9.30 \times 10^{-6} \rightarrow$ enforced to 1.12×10^{-5}	$\mathbf{1.12 \times 10^{-5}}$	**Significant**
**3**	Genomic signature family (F2)	1.2×10^{-4}	$\times 2 = 2.40 \times 10^{-4}$	$\mathbf{2.40 \times 10^{-4}}$	**Significant**
**4**	Spatial chemokine axis (F5)	2.1×10^{-4}	$\times 1 = 2.10 \times 10^{-4} \rightarrow$ enforced to 2.40×10^{-4}	$\mathbf{2.40 \times 10^{-4}}$	**Significant**

The Bonferroni-Holm step-down procedure (Holm 1979) orders P-values from smallest to largest and applies multipliers of m, m−1, ..., 1 where m is the number of tests. Monotonicity enforcement prevents paradoxically smaller adjusted P-values at later ranks: the adjusted P at rank k is set to max(Holm-adjusted P at rank k, adjusted P at rank k−1). All four primary

mechanistic axes remained significant at a strict family-wise error rate (FWER) threshold of 0.05, confirming that the SMARCAL1-ferroptosis-chemokine-immune axis is jointly robust to the most conservative cross-family correction procedure applicable.

Sensitivity analyses: all principal findings also held under (i) Benjamini-Yekutieli correction valid under arbitrary dependence (Benjamini & Yekutieli 2001); (ii) Storey's adaptive q-value (Storey 2002) for transcriptome-wide spatial DE; and (iii) primary permutation-based P-values (B = 1,000) for all spatial analyses with small sample sizes. See Supplementary Figure S10 for sensitivity comparison.

Abbreviations: BH, Benjamini-Hochberg; BY, Benjamini-Yekutieli; DE, differential expression; FDR, false discovery rate; FWER, family-wise error rate; KEGG, Kyoto Encyclopedia of Genes and Genomes; MRD, molecular residual disease.

Supplementary Table S7. **Top 20 significant CellChatV2 ligand-receptor interactions in the expanded Visium cohort (n = 14 patients; 36,318 spots; Family F5, q < 0.01 stringent threshold).**

Rank	Ligand (cell source)	Receptor (target)	CellChat Pathway	Weight High	Weight Low	Raw P (perm)	Spatial Pattern
**1**	*CXCL9 (tumor/myeloid)*	*CXCR3 ($CD8^+$ T)*	CXCL chemokine signaling	0.842	0.118	< 0.001	Peritumoral rim
**2**	*CXCL10 (tumor/myeloid)*	*CXCR3 ($CD8^+$ T)*	CXCL chemokine signaling	0.786	0.142	< 0.001	Peritumoral rim
**3**	*CXCL11 (myeloid)*	*CXCR3 ($CD8^+$ T)*	CXCL chemokine signaling	0.521	0.098	< 0.001	Peritumoral rim
**4**	*CCL5 ($CD8^+$ T)*	*CCR5 (macrophages)*	CCL chemokine signaling	0.612	0.184	< 0.001	Tumor nest
**5**	*HLA-A (tumor)*	*CD8A ($CD8^+$ T)*	MHC-I antigen presentation	0.738	0.326	< 0.001	Tumor nest
**6**	*HLA-B (tumor)*	*CD8A ($CD8^+$ T)*	MHC-I antigen presentation	0.692	0.298	< 0.001	Tumor nest
**7**	*IFNG ($CD8^+$ T)*	*IFNGR1 (tumor)*	IFN type II signaling	0.634	0.187	< 0.001	Peritumoral rim
**8**	*TNF ($CD8^+$ T)*	*TNFRSF1A (tumor)*	TNF signaling	0.541	0.231	< 0.001	Peritumoral rim
**9**	*GZMB ($CD8^+$ T)*	*— (tumor cell death)*	Effector cytotoxicity	0.728	0.142	< 0.001	Tumor nest (invasion)
**10**	*PRF1 ($CD8^+$ T)*	*— (tumor cell death)*	Effector cytotoxicity	0.684	0.136	< 0.001	Tumor nest (invasion)
**11**	*CXCL12 (stroma)*	*CXCR4 ($CD8^+$ T/tumor)*	CXCL chemokine signaling	0.421	0.186	0.003	Stromal compartment
**12**	*CCL2 (tumor/myeloid)*	*CCR2 (monocytes)*	CCL chemokine signaling	0.362	0.218	0.008	Tumor nest
**13**	*IL-15 (myeloid)*	*IL-15RA (NK/T)*	IL-15 signaling	0.348	0.124	0.002	Peritumoral rim
**14**	*TIGIT-PVR axis (tumor)*	*TIGIT ($CD8^+$ T)*	Immune checkpoint	0.286 (high)	0.162 (high)	0.012	Tumor nest
**15**	*PDCD1LG2 (PD-L2, tumor)*	*PDCD1 ($CD8^+$ T)*	Immune checkpoint	0.341 (high)	0.189 (high)	0.018	Tumor nest
**16**	*CD274 (PD-L1, tumor)*	*PDCD1 ($CD8^+$ T)*	Immune checkpoint	0.428 (high)	0.213 (high)	0.002	Tumor nest
**17**	*LGALS9 (tumor)*	*HAVCR2 (TIM-3, $CD8^+$ T)*	Immune checkpoint	0.312 (high)	0.178 (high)	0.014	Tumor nest
**18**	*VEGFA (tumor)*	*FLT1 / KDR (endothelial)*	Angiogenesis signaling	0.284 (low)	0.421 (low)	< 0.001	Tumor vasculature
**19**	*TGFB1 (stroma/Treg)*	*TGFBR2 (tumor/T)*	TGF-β signaling	0.198 (low)	0.387 (low)	< 0.001	Tumor nest
**20**	*CCL17 (DC)*	*CCR4 (Treg)*	CCL chemokine signaling	0.184 (low)	0.342 (low)	< 0.001	Peritumoral rim

CellChatV2 (Jin et al. Nat Commun 2024) was applied to Harmony-integrated Visium data (n = 14 patients; 36,318 spots) using the full human CellChatV2 ligand-receptor database (~3,000 curated pairs). Interaction weights are unitless composite scores combining ligand and receptor expression probabilities with curated signaling strength. 'High' and 'Low' columns refer to average weights within High Rad-Score (5 patients: P01, P02, P03, P04, P05) vs Low Rad-Score (5 patients: P06, P07, P08, P09, P10) subsets, with intermediate samples P11-P14 excluded from the binary comparison. Permutation-based P-values (B = 1,000 cell-label permutations per pair) were corrected using Benjamini-Hochberg FDR with stringent threshold $q < 0.01$ given the ~3,000-pair test burden.

Spatial patterns were determined by per-spot CellChat communication-probability mapping onto Visium coordinates. 'Peritumoral rim' refers to a 3-mm annular region defined centripetally from the pan-cytokeratin^{+} tumor boundary; 'tumor nest' refers to pan-cytokeratin^{+} Visium spots; 'stromal compartment' refers to CD10^{+} / vimentin^{+} spots; 'tumor vasculature' refers to CD31^{+} endothelial spots.

Interpretation: ranks 1–10 reflect the dominant cytotoxic-recruitment axis (CXCL9/10–CXCR3, effector T cell activity, and MHC-I antigen presentation) preferentially active in High Rad-Score tumors. Ranks 14–17 show checkpoint axes also upregulated in High tumors, consistent with a 'hot-and-exhausted' immune phenotype. Ranks 18–20 show immunosuppressive/angiogenic axes preferentially active in Low Rad-Score tumors, consistent with a 'cold' tumor microenvironment.

Abbreviations: DC, dendritic cell; IFN, interferon; IL, interleukin; MHC-I, major histocompatibility complex class I; NK, natural killer; PD-L1/2, programmed death-ligand 1/2; perm, permutation-based P value; TGF-β, transforming growth factor β; TIGIT, T cell immunoreceptor with Ig and ITIM domains; TIM-3, T cell immunoglobulin and mucin-domain containing-3; TNF, tumor necrosis factor; Treg, regulatory T cell; VEGFA, vascular endothelial growth factor A.

Supplementary Table S8. **Head-to-head comparison of the multimodal nomogram (Rad-Score + TNM + TMB + Age) against five established or emerging prognostic comparators: ΔC-index, continuous NRI, IDI, and Decision Curve Analysis net benefit.**

Comparator Prognostic Score	Comparator C-index (95% CI)	Multimodal C-index (95% CI)*	ΔC-index (95% CI)	Continuous NRI (95% CI)	IDI (95% CI)
Training Cohort (N = 71; High n = 35, Low n = 36)					
**CALGB 9761-type clinicopathological score†**	0.691 (0.612–0.770)	0.841 (0.779–0.903)	+0.150 (0.083–0.217)	+0.51 (0.32–0.70)	+0.12 (0.06–0.18)
**Clinical + TMB (age, stage, TMB)**	0.711 (0.638–0.784)	0.841 (0.779–0.903)	+0.130 (0.071–0.189)	+0.47 (0.28–0.66)	+0.11 (0.06–0.16)
**Kratz 14-gene RNA signature‡**	0.751 (0.678–0.824)	0.841 (0.779–0.903)	+0.090 (0.041–0.139)	+0.36 (0.18–0.54)	+0.08 (0.04–0.12)
**ctDNA maximum VAF (Chabon-type)§**	0.762 (0.691–0.833)	0.841 (0.779–0.903)	+0.079 (0.032–0.126)	+0.34 (0.16–0.52)	+0.08 (0.04–0.12)
**Composite ctDNA-informed score¶**	0.760 (0.688–0.832)	0.841 (0.779–0.903)	+0.081 (0.036–0.126)	+0.31 (0.15–0.47)	+0.07 (0.03–0.11)
**Bindea 28-cell ssGSEA immunoscore‖**	0.771 (0.701–0.841)	0.841 (0.779–0.903)	+0.070 (0.028–0.112)	+0.28 (0.12–0.44)	+0.06 (0.02–0.10)
External NSCLC-Radiogenomics Cohort (n = 178)					
CALGB 9761-type	0.648 (0.582–0.714)	0.783 (0.721–0.845)	+0.135 (0.086–0.184)	+0.42 (0.26–0.58)	+0.10 (0.05–0.15)
Clinical + TMB (imputed TMB, n = 127)	0.672 (0.598–0.746)	0.776 (0.703–0.849)	+0.104 (0.051–0.157)	+0.38 (0.19–0.57)	+0.09 (0.04–0.14)
Kratz 14-gene (microarray projection)	0.698 (0.631–0.765)	0.783 (0.721–0.845)	+0.085 (0.038–0.132)	+0.31 (0.14–0.48)	+0.07 (0.03–0.11)
Bindea 28-cell (microarray projection)	0.716 (0.652–0.780)	0.783 (0.721–0.845)	+0.067 (0.028–0.106)	+0.24 (0.08–0.40)	+0.05 (0.02–0.08)
External Lung3 Cohort (n = 89; Rad-Score alone vs comparators)					
CALGB 9761-type	0.628 (0.532–0.724)	0.741 (0.651–0.831)#	+0.113 (0.048–0.178)	+0.38 (0.18–0.58)	+0.08 (0.03–0.13)
Bindea 28-cell	0.681 (0.594–0.768)	0.741 (0.651–0.831)#	+0.060 (0.018–0.102)	+0.22 (0.06–0.38)	+0.04 (0.01–0.07)

Decision Curve Analysis Net Benefit at Threshold Probability 0.30

Prediction Strategy	Net Benefit (Training)	Net Benefit (NSCLC-RG)	Net Benefit (Lung3)	Clinically Meaningful?**
**Multimodal nomogram**	**0.253**	**0.219**	**— (n/a)**	**Yes**
**Rad-Score alone**	**0.241**	**0.201**	**0.187**	**Yes**
Clinical + TMB	0.200	0.172	0.158 (clin only)	Yes
Bindea immunoscore	0.214	0.180	0.167	Yes
Kratz 14-gene	0.205	0.174	—	Yes
CALGB 9761-type	0.200	0.166	0.146	Yes
Treat-all (assume MRD will convert)	0.172	0.142	0.132	Reference
Treat-none (observation only)	0.000	0.000	0.000	Reference

**Multimodal nomogram = age + pathological stage + TMB (continuous) + Rad-Score (continuous), fit on the full training cohort of N = 71 (High n = 35, Low n = 36).*

†CALGB 9761 was a prospective study identifying clinicopathological prognostic factors for early-stage NSCLC (D'Cunha et al. J Thorac Oncol 2005); the 'CALGB 9761-type' score is a combined age + stage + histology + tumor size + lymph node count predictor reconstructed from the published variables.

‡Kratz et al. Lancet 2012 14-gene qPCR signature (BAG1, BRCA1, CDC6, CDK2AP1, ERBB3, FUT3, IL11, LCK, RND3, SH3BGR, STX1A, TFRC, TMEM45B, WNT3A) validated in multiple independent lung adenocarcinoma cohorts.

§ctDNA maximum variant allele frequency (VAF) from pre-operative blood ctDNA as defined in Chabon et al. Nature 2020 CAPP-Seq assay.

¶Composite ctDNA-informed score combines pre-operative maximum VAF, ΔVAF (postoperative day 3 minus day 30), and sum of detected variants (weighted per Chabon methodology).

‖Bindea et al. Immunity 2013 28-immune-cell-type gene signatures quantified via single-sample gene set enrichment analysis (ssGSEA; GSVA R package v1.46.0); PMID 24138885.

#Multimodal nomogram not applicable in Lung3 due to unavailable TMB; Rad-Score alone C-index reported for reference comparison.

***Clinical significance threshold per DCA methodology: Δ net benefit ≥ 0.02 at the threshold probability matched to clinical decision context (here, 0.30 for adjuvant therapy consideration). All comparisons achieved Δ NB > 0.02 versus treat-all strategy.*

Statistical methodology: Continuous NRI per Pencina et al. Stat Med 2011 with Kerr et al. 2014 correction for proportion reclassified up/down; IDI per Pencina et al. Stat Med 2008 at the 0.03 clinical threshold; partial C-index computed via rcorrp.cens function (Hmisc R package v5.1-0); Decision Curve Analysis via rmda R package v1.6.

All primary Rad-Score nomogram comparisons achieved statistical significance at P < 0.01 for all three metrics (ΔC-index, continuous NRI, IDI) after Benjamini-Hochberg correction within the benchmarking family (6 comparators × 3 metrics × 3 cohorts = 54 tests).

Abbreviations: CALGB, Cancer and Leukemia Group B; C-index, concordance index; CI, confidence interval; clin, clinical; ctDNA, circulating tumor DNA; DCA, decision curve analysis; IDI, integrated discrimination improvement; NB, net benefit; NRI, net reclassification improvement; n/a, not applicable; NSCLC, non-small cell lung cancer; qPCR, quantitative polymerase chain reaction; Rad-Score, radiomic score; ssGSEA, single-sample gene set enrichment analysis; TMB, tumor mutational burden; VAF, variant allele frequency.

Supplementary Table S9. **Protein-level validation of SMARCAL1-ferroptosis-CXCR3⁺ CD8⁺ immune axis by multiplex immunofluorescence (mIF, 40 patients) and chromogenic immunohistochemistry (IHC, 60 patients), stratified by Rad-Score quartile.**

Biomarker / Compartment	Q1 (n = 10)	Q2 (n = 10)	Q3 (n = 10)	Q4 (n = 10)	Jonckheere-Terpstra P*	Spearman ρ†
Multiplex Immunofluorescence — 40 patients (HALO quantification, cells per mm²)						
**SMARCAL1⁺ tumor cells**	182 ± 44	247 ± 61	354 ± 79	478 ± 98	< 0.001	+0.84
**ACSL4⁺ tumor cells**	96 ± 28	164 ± 42	226 ± 54	312 ± 71	< 0.001	+0.79
**4-HNE⁺ ferroptotic cells (tumor core)**	43 ± 14	78 ± 21	131 ± 32	187 ± 46	< 0.001	+0.82
**GPX4⁺ anti-ferroptotic cells**	212 ± 48	168 ± 39	112 ± 28	78 ± 21	< 0.001	−0.76
**SMARCAL1⁺ACSL4⁺4-HNE⁺ triple-positive cells**	18 ± 7	37 ± 12	68 ± 18	94 ± 24	< 0.001	+0.87
**CXCR3⁺ CD8⁺ T cells (peritumoral)**	21 ± 8	34 ± 12	58 ± 17	82 ± 22	< 0.001	+0.85
**CD8⁺ T cells (total TIL)**	58 ± 16	92 ± 24	148 ± 38	218 ± 52	< 0.001	+0.83
**FoxP3⁺ regulatory T cells**	42 ± 12	38 ± 11	28 ± 9	22 ± 7	< 0.001	−0.68
**CD4⁺ helper T cells**	67 ± 18	89 ± 23	124 ± 32	162 ± 41	< 0.001	+0.72
**CD68⁺ CD163⁻ M1 macrophages**	34 ± 11	52 ± 16	78 ± 21	103 ± 28	< 0.001	+0.74
**CD68⁺ CD163⁺ M2 macrophages**	89 ± 24	72 ± 19	54 ± 16	41 ± 13	< 0.001	−0.71
**CXCL9⁺ signal intensity (MFI, tumor)**	842 ± 186	1,467 ± 324	2,831 ± 612	3,864 ± 823	< 0.001	+0.86
**CXCL10⁺ signal intensity (MFI, tumor)**	738 ± 162	1,246 ± 284	2,142 ± 467	2,876 ± 634	< 0.001	+0.83
Nearest-Neighbor Spatial Analysis — 40 patients (median distance in µm, IQR)						
**CXCR3⁺ CD8⁺ T cells to CXCL9⁺ tumor cells**	127 (94–161)	96 (68–124)	52 (34–71)	34 (22–48)	< 0.001	−0.82
**CD8⁺ TIL to tumor cell nuclei**	186 (142–234)	138 (102–178)	78 (56–104)	46 (31–68)	< 0.001	−0.79
**FoxP3⁺ Treg to CD8⁺ T cells**	96 (71–124)	118 (87–152)	182 (136–231)	246 (184–318)	< 0.001	+0.76
Orthogonal IHC Validation — 60 patients (H-score on 0–300 scale)						
**SMARCAL1 H-score**	62 ± 21	94 ± 28	142 ± 38	198 ± 49	< 0.001	+0.71
**ACSL4 H-score**	48 ± 18	76 ± 24	118 ± 32	162 ± 42	< 0.001	+0.63
**GPX4 H-score**	186 ± 38	148 ± 32	108 ± 26	72 ± 19	< 0.001	−0.67
**CD8⁺ TIL density (cells per mm²)**	42 ± 14	68 ± 19	112 ± 29	168 ± 41	< 0.001	+0.68
**CXCR3 expression H-score**	38 ± 12	62 ± 18	98 ± 26	142 ± 36	< 0.001	+0.59

**Jonckheere-Terpstra trend test for monotonic trend across ordered Rad-Score quartiles (Q1 lowest, Q4 highest; 10 patients per quartile in the mIF cohort and 15 per quartile in the IHC cohort), two-sided.*

†Spearman rank correlation coefficient between continuous Rad-Score and biomarker density or H-score; all P < 0.001.

Validation cohort composition: the 40-patient mIF subcohort and 60-patient IHC subcohort were drawn from the 71-patient training cohort (35 High + 36 Low Rad-Score) with balanced representation across histology, stage, and sex; 15–20 patients contributed tissue to both validation platforms. Quartile assignment was based on the continuous Rad-Score value.

Multiplex IF panels used the Opal 7-color methodology (Akoya Biosciences) with Vectra Polaris imaging and HALO v3.6.4 quantification (Indica Labs). Two Opal panels (7 markers each) with pan-cytokeratin and DAPI shared across panels ensured cross-panel alignment.

Chromogenic IHC H-scores were independently reviewed by two pathologists blinded to Rad-Score status (Cohen's kappa = 0.82 for inter-observer agreement). H-score = Σ (staining intensity [0–3]) × (% cells at that intensity).

Abbreviations: 4-HNE, 4-hydroxynonenal; ACSL4, acyl-CoA synthetase long-chain family member 4; CXCL9/10, C-X-C motif chemokine ligand 9/10; CXCR3, C-X-C motif chemokine receptor 3; FoxP3, forkhead box P3; GPX4, glutathione peroxidase 4; IHC, immunohistochemistry; IQR, interquartile range; M1/M2, macrophage polarization subtypes; MFI, mean fluorescence intensity; mIF, multiplex immunofluorescence; Q1–Q4, Rad-Score quartiles (1 = lowest, 4 = highest); SMARCAL1, SWI/SNF-related matrix-associated actin-dependent regulator of chromatin subfamily A-like 1; TIL, tumor-infiltrating lymphocytes; Treg, regulatory T cell.
